# *Thy1*-GCaMP6 Transgenic Mice for Neuronal Population Imaging *In Vivo*

**DOI:** 10.1371/journal.pone.0108697

**Published:** 2014-09-24

**Authors:** Hod Dana, Tsai-Wen Chen, Amy Hu, Brenda C. Shields, Caiying Guo, Loren L. Looger, Douglas S. Kim, Karel Svoboda

**Affiliations:** Janelia Farm Research Campus, Howard Hughes Medical Institute, Ashburn, Virginia, United States of America; Baylor College of Medicine, United States of America

## Abstract

Genetically-encoded calcium indicators (GECIs) facilitate imaging activity of genetically defined neuronal populations *in vivo*. The high intracellular GECI concentrations required for *in vivo* imaging are usually achieved by viral gene transfer using adeno-associated viruses. Transgenic expression of GECIs promises important advantages, including homogeneous, repeatable, and stable expression without the need for invasive virus injections. Here we present the generation and characterization of transgenic mice expressing the GECIs GCaMP6s or GCaMP6f under the *Thy1* promoter. We quantified GCaMP6 expression across brain regions and neurons and compared to other transgenic mice and AAV-mediated expression. We tested three mouse lines for imaging in the visual cortex *in vivo* and compared their performance to mice injected with AAV expressing GCaMP6. Furthermore, we show that GCaMP6 *Thy1* transgenic mice are useful for long-term, high-sensitivity imaging in behaving mice.

## Introduction

Optical imaging of calcium dynamics is commonly used for monitoring activity in neuronal ensembles and micro-compartments. For example, using 2-photon microscopy the activity of hundreds of cells has been measured during behavior [1], [2]. Continued development of genetically encoded calcium indicators (GECIs) has enabled a shift from synthetic indicators, such Fluo-4 and Oregon Green BAPTA-1 [3], to protein indicators [4], [5], [6], [7], [8], [9]. GECIs can be introduced to the brain using relatively noninvasive gene transfer methods such as viral infection using adeno-associated viruses (AAVs) [4], [10]. Neurons expressing GECIs can be monitored over weeks [1], [4], [9], [11]. The recently developed GCaMP6 indicators allow sensitive detection of activity, under favorable circumstances down to single action potentials (APs) [7].

AAVs can produce the high intracellular GECI concentrations (∼10–100 µM) required for *in vivo* imaging [1], [12]. However, AAVs produce different expression levels in neighboring neurons and gradients in expression levels across the infection site [4], [7]. In addition, GECI expression levels continue to rise over time until they can cause aberrant cell health [4], [12]. The time window for GECI imaging is thus typically limited to a few weeks, depending on the promoter construct, viral titer, injection volume, and other factors. Finally, AAV-mediated gene transfer requires challenging surgeries. Best-practice procedures demand tiny injection volumes (approximately 50 nl) [1], which can result in variable numbers of infected cells with variable GECI expression levels.

Transgenic methods can produce stable expression of GECIs over longer time scales [12], [13], [14], potentially over the entire lifetime of the mouse, without invasive procedures for gene transfer. Expression patterns and levels are reproducible across different individual animals [12]. Several transgenic GECI mouse lines have been developed [12], [13], [14], [15], [16], [17], [18], [19], [20], which have demonstrated the advantages of transgenic control of protein expression. Here we present the development and characterization of transgenic mouse lines expressing GCaMP6s and GCaMP6f GECIs under the *Thy1* promoter [20], [21], [22]. We characterize the brain-wide expression patterns of each line, and the performance of selected lines for cellular *in vivo* imaging.

## Materials and Methods

All surgical and experimental procedures were in accordance with protocols approved by the Janelia Farm Institutional Animal Care and Use Committee and Institutional Biosafety Committee.

### Transgenic mice

Here we report on GENIE Project (GP) lines GP4.x (where ‘x’ refers to the founder number) expressing GCaMP6s, and GP5.x expressing GCaMP6f. *Thy1*-GCaMP6-WPRE transgenic mice were generated using standard techniques [23].We included the WPRE (Woodchuck hepatitis virus post-transcriptional regulatory element), which increases mRNA stability and protein expression [24], [25]. Genotyping primers were 5′-CATCAGTGCAGCAGAGCTTC-3′ (forward, anneals to calmodulin sequence in GCaMP6) and 5′-CAGCGTATCCACATAGCGTA-3′ (reverse, anneals to WPRE sequence). Mouse lines GP4.3, 4.12, 5.5, 5.11 and 5.17 were deposited at The Jackson Laboratory (acquisition numbers provided at end).

### Expression analysis

**Figure 1:**
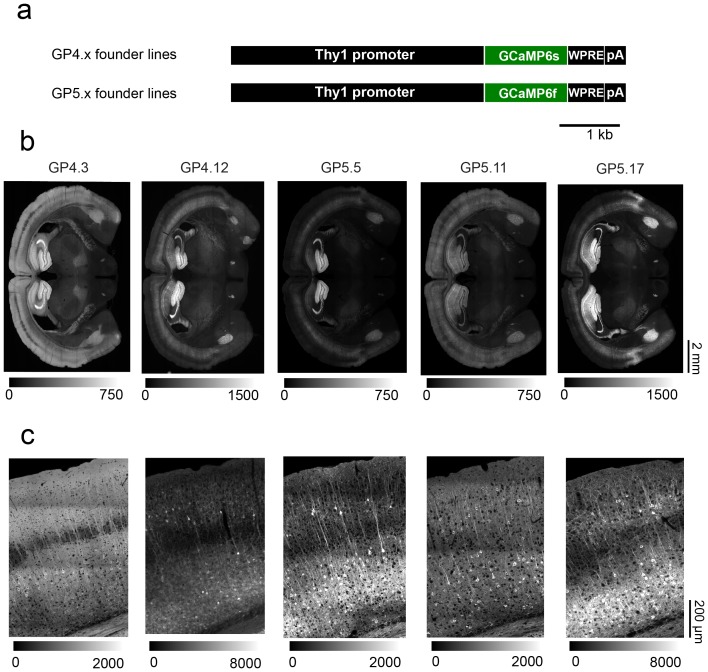
*Thy1* transgenic mice expressing GCaMP6s or GCaMP6f. **a**. Schematic of the transgene cassettes used to generate GP4.x (top) and GP5.x (bottom) lines. WPRE = Woodchuck hepatitis virus post-transcriptional regulatory element, pA = poly-adenylation tail. **b**. Wide-field images of coronal sections showing GCaMP fluorescence in various transgenic lines. **c**. Representative confocal images (tiled and stitched to show larger field of view) from the somatosensory cortex of the same lines as in **b**. All images show GCaMP6 fluorescence.

**Figure 2:**
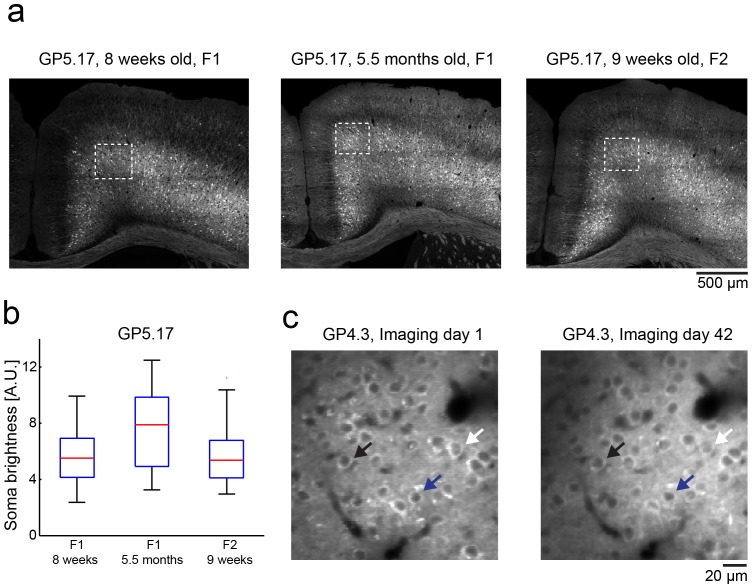
Stable GCaMP expression in GP mice over months. **a**. Confocal microscope images of fixed coronal sections from the motor cortex of GP5.17 mice at different ages (F1- first generation, F2- second generation). **b**. Somatic GCaMP6f brightness for all neurons inside the white rectangles in **a** (51–65 neurons from each animal). For each mouse, the box indicates the 25^th^ to 75^th^ percentile distribution, red line indicates the median, and whisker length is 150% of the 25^th^ to 75^th^ percentile distance, or until it touches the last sample position. Outliers are marked in red crosses. **c**. *In vivo* two-photon microscopy images of GP4.3 mice taken at different days after cranial window implantation show similar expression pattern without filled nuclei. Arrowheads point to three individual cells in both images.

Adult mice (P42–P56) were deeply anesthetized with isofluorane and transcardially perfused with 10 ml 1× Dulbecco's phosphate-buffered saline (DPBS, Life Technologies), followed by 50 ml 4% paraformaldehyde in 0.1 M phosphate buffer. After perfusion, the brains were removed and post-fixed overnight at 4°C. The brains were embedded in 5% agarose in DPBS, and cut into 50 µm thick coronal sections with a vibratome (Leica VT 1200S). Since DPBS contains a saturating concentration of calcium (0.9 mM) GCaMP brightness will be maximal. Every other section was dehydrated with DPBS and coverslipped with Vectashield mounting medium (H-1400, Vector laboratories). The coverslipped sections were imaged using a slide scanner (Nanozoomer, Hamamatsu). Confocal images (LSM 710, Zeiss) were collected for selected brain regions (Fig. 1 and 2, Fig. S1 and S3) [26], using an 20× 0.8 NA objective and standard GFP imaging filters. Individual images were tiled and stitched using commercial software (Zeiss).

For a subset of mouse lines (GP4.3, GP4.12, GP5.5, GP5.11, and GP5.17) we visualized neurons using NeuN to measure the fraction of neurons expressing GCaMP. Staining was performed on sections that were not used for quantification of expression. Sections were blocked with 2% BSA and 0.4% Triton X-100 solution for 1 hour at room temperature to prevent nonspecific antibody binding, followed by incubation overnight at 4°C with mouse anti-NeuN primary antibody (1∶500; Millipore, MAB 377) and incubation with Alexa594-conjugated goat-anti-mouse secondary antibody (1∶ 500; Life Technologies, A11032) for 4 hours at room temperature. Sections were mounted on microscope slides with Vectashield mounting medium (H-1400, Vector laboratories).

We analyzed primary motor cortex (M1), primary somatosensory cortex (S1), primary visual cortex (V1) and hippocampus (CA1, CA3, and Dentate Gyrus, DG) using confocal microscopy. For sample images in each area we identified all labeled cells, segmented their somata, and calculated the somatic GCaMP fluorescence brightness for each cell. For cortical regions, cells were grouped into layer 2/3 (L2/3) and layer 5 (L5) cells. We also counted the fraction of GCaMP labeled cells (green channel) as a fraction of the NeuN stained cells (red channel). To compensate for variations of imaging conditions across time (e.g. changes in the excitation light source intensity), images of a fluorescence standard, 3.8 µm fluorescent beads (Ultra Rainbow Fluorescent Particles, Bangs Laboratories), were acquired. The average bead brightness was used to normalize the GCaMP signal.

In addition we performed a coarse analysis of expression levels across numerous brain regions (Table 1; Data S1).

**Table 1:** GCaMP6 expression at the level of brain regions.

Line	Olfactory bulb	M1	Piriform area	Amygdala	S1	Hippo-campus	Thala-mus	Hypo-thalamus	V1	Cere-bellum	Mid- brain	Pons	Medulla
GP4.1		++	+	+	++	+++	++	+	++	+	+	+	+
GP4.2	+	−	−	−	+	++	+	−	+	+	−	+	+
GP4.3	+	++	+	+	++	+++	+	−	++	−	+	+	+
GP4.4	+	+	+	+	+	+	+	−	+	+	+	+	+
GP4.6	+	+	+	−	+	++	+	−	+	+	+	+	+
GP4.7	+	L6 ++	−	−	L6 ++	−	+	−	L6 ++	+	−	+	−
GP4.9	−	L5a ++	−	+	L5a ++	+++	−	+	L5a ++	−	−	+	−
GP4.12	++	+++	++	+++	+++	+++	+	+	++	−	+	+	−
GP4.14	−	++	−	−	++	++	−	−	+	−	−	+	
GP4.15	+	−	−	−	+	+	+	−	+	+	+	+	+
GP4.17	+	++	++	−	++	+++	+	+	+	−	−	+	−
GP5.1	++	++	++	++	++	+++	+	+	++	+	+	+	−
GP5.3	++	++	++	+	++	+++	+	−	++	+	−	++	+
GP5.5	++	++	+	++	++	+++	+	+	++	−	−	+	−
GP5.9		++	+	++	++	+++	+	+	++	−	+	+	−
GP5.10	−	+	−	−	+	+	+	−	+	−	−	+	−
GP5.11	++	++	+	+	++	++	+	+	++	+	−	+	−
GP5.12	+	+	++	+	++	++	++	+	++	+	−	+	+
GP5.14	+	+	+	+	+	+	+	+	+	+	+	+	+
GP5.15	+	+	+	+	+	+	+	+	+	−	−	+	+
GP5.17	++	+++	+	+++	+++	+++	+	+	+++	−	+	++	+
GP5.18	+	+	+	−	+	+	+	+	+	+	+	+	+
GP5.21	+	++	+	++	++	+++	+	+	++	+	−	+	+

GCaMP6 brightness was scored based on widefield microscope images of coronal sections. Note: no effort was made to separate axonal projections and somato-dendritic signal. Legend: − no signal, + weak signal, ++ moderate signal, +++ strong signal. See Data S1 for raw images.

### Mouse preparation for V1 *in vivo* imaging

For cranial window surgery mice were anesthetized using isoflurane (2.5% for induction, 1.5–2% during surgery). A circular craniotomy (2–2.5 mm diameter) was made above V1 (centered 2.7 mm left, and 0.2 mm anterior to Lambda suture) and covered with 1% agarose. A 3 mm round glass coverslip (no. 1 thickness, Warner Instruments) was cemented to the brain using black dental cement (Contemporary Ortho-Jet). A custom titanium head post was cemented to the skull. The animal was then placed under a microscope on a warm blanket (37°C) and kept anesthetized using 0.5% isoflurane and sedated with chlorprothixene (20–40 µl at 0.33 mg/ml, i.m.) [27].

### *In vivo* mouse imaging in V1

Imaging was performed with a custom-built two-photon microscope with a resonant scanner (designs available at http://research.janelia.org/svoboda/). The light source was a Mai Tai HP 100 femtosecond-pulse laser (Spectra-Physics) running at 940 nm. The objective was a 16× water immersion lens with 0.8 NA (Nikon). Images were acquired using ScanImage 4 (vidriotechnologies.com) [28]. Functional images (512×512 pixels, 250×250 µm^2^) of L2/3 cells (100–250 µm under the pia) were collected at 15 Hz. Laser power was 145 mW at the front aperture of the objective.

Visual stimuli were moving gratings generated using the Psychophysics Toolbox [29], [30] in MATLAB (Mathworks), presented using an LCD monitor (30×40 cm), placed 25 cm in front of the center of the right eye of the mouse. Each stimulus trial consisted of a 4 s blank period (uniform gray display at mean luminance) followed by a 4 s drifting sinusoidal grating (0.05 cycles/degree, 1 Hz temporal frequency, 8 different directions). The stimuli were synchronized to individual image frames using frame-start pulses provided by ScanImage 4. The monitor subtended an angle of ±38° horizontally and ±31° vertically around the eye of the mouse.

### Analysis of V1 functional imaging

All analyses were performed in MATLAB. Regions of interest (ROIs) corresponding identifiable cell bodies were selected using a semi-automated algorithm [6], [7]. Depending on the neuron's appearance, annular [4] or circular ROIs were placed over the cytosolic regions of each cell. The fluorescence time course was measured by averaging all pixels within the ROI, after correction for neuropil contamination [31]. The neuropil signal F_neuropil_(t) surrounding each cell was measured by averaging the signal of all pixels within a 20 µm circular region from the cell center (excluding all somata). The fluorescence signal of a cell body was estimated as
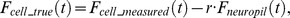
with r = 0.7 [7]. Although we used one value for r across preparations, for optimal neuropil correction r may have to be adjusted for different mouse lines and experimental conditions. Neuropil correction was applied only to cells with baseline fluorescence (F_0_) signal stronger than the surrounding neuropil signal by more than 3%; other cells (approximately 10%) were excluded from the analysis because F_0_ could not be reliably estimated. After neuropil correction, the ΔF/F_0_ of each trial was calculated as (F−F_0_)/F_0_, where F_0_ was averaged over a 2 s period for GCaMP6f experiments and 1 s for GCaMP6s experiments immediately before the start of grating stimulation. Visually responsive neurons were defined as cells with ΔF/F_0_>0.05 during at least one stimulus period, and using ANOVA across blank and eight direction periods (p<0.01) [32].

For calculating the mean response to the preferred stimulus, traces for cells with large responses (ΔF/F_0_>1) were averaged. Because each cell responded at slightly different times, depending on its receptive field structure, each trace was shifted so that their maxima align. For visual display, traces were smoothed with a 3 sample moving average kernel.

We calculated the decay time of fluorescence after the end of the preferred stimulus. For each cell we averaged responses from five trials; baseline fluorescence and standard deviation were calculated from 1 s (GCaMP6s) or 2 s (GaMP6f) before the start of the stimulus. Only responsive cells with fluorescence response 5 times the standard deviation of the baseline during the last 1 s of the stimulus were analyzed. The time required for each trace to reach half of its peak value (baseline fluorescence subtracted) was calculated by linear interpolation.

### AAV injection

Adult mice (P42–56) were anesthetized and injected with AAV-*synapsin1*-GCaMP6s (AAV-6s) or AAV-*synapsin1*-GCaMP6f (AAV-6f) into the primary visual cortex (2 injections, 25 nl each, centered 2.5 and 2.9 mm left, and 0.2 mm anterior to Lambda suture) [7]. Ai38 mice were injected with Cre-expressing AAV virus under the human *synapsin1* promoter. 3–4 weeks post-injection, mice were implanted with a cranial window and imaged on the same day. After the imaging session, mice were perfused and their brains were fixed. Confocal microscopy images of V1 were collected for expression analysis (Fig. S1); cells with filled nuclei (∼5% of the total) were excluded from analysis.

### *In vivo* imaging in anterior lateral motor cortex during behavior

A circular craniotomy (2–3 mm diameter) was made above left anterior lateral motor cortex (ALM) cortex (centered at 2.5 mm anterior and 1.5 mm lateral to Bregma) [33]. The imaging window, constructed from two layers of microscope coverglass [1], was fixed to the skull using cyanoacrylate glue and dental acrylic. Behavioral training started ∼2 weeks after window surgery [34]. Imaging was performed using a resonant scanning two-photon microscope controled by ScanImage 4 [28]. Images (512×512 pixels) covering a field of view of ∼600×600 µm were acquired at 15 Hz. The laser wavelength was 940 nm and the power used was between 70–120 mW at the front aperture of the objective.

## Results

We screened 13 lines of *Thy1*-GCaMP6s mice and 16 of *Thy1*-GCaMP6f mice. 11 *Thy1*-GCaMP6s lines expressed GCaMP6s (lines ‘GP4.x’, where ‘x’ refers to the founder) and 12 *Thy1*-GCaMP6f expressed GCaMP6f (lines ‘GP5.x’) (Fig. 1a). Because of the strong dependence of expression level and labeling pattern on transgene cassette integration site in the founder mouse genome [20], [21], significant differences were found between these lines. Lines GP4.3, GP4.12, GP5.5, GP5.11, and GP5.17 showed robust expression with some unique features and were analyzed in more depth (Fig. 1b–c; Table 1; Fig. S2, Data S1). Images of tissue sections and an analysis of expression levels across brain regions are available in the supplemental materials (Table 1; Data S1).

### Characterization of the GP lines

Expression was similar across different individual mice from the same line (Fig. 2 a, b; Fig. S2). In long-term imaging experiments in adult mice GECI concentration was stable over time (Fig. 2c). With AAV infection, long-term expression can cause accumulation of GCaMP6 in the nucleus, a correlate of cytomorbidity [4], [7], [12]. In the GP lines GCaMP6 remained excluded from the nucleus (Fig. 2c), even in 11-month old mice in all brain regions examined (data not shown).

**Figure 3:**
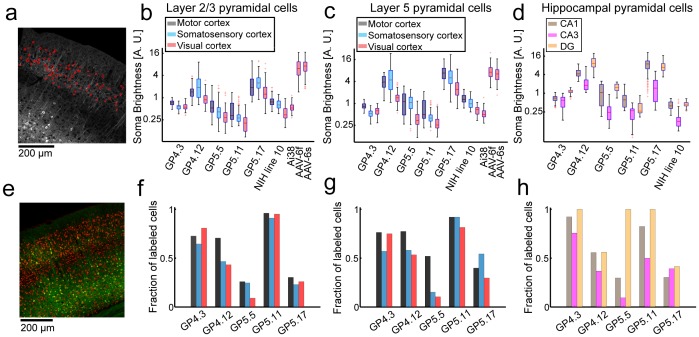
Quantification of GCaMP expression. **a**. Demonstration of the analysis method used for calculating single-neuron brightness distribution across brain regions. Confocal microscopy images of fixed brain slices were used for segmentation into cell bodies (red rings, nuclei were excluded from somata). Somatic brightness was calculated by averaging all pixels in each segmented cell. **b–d**. Neuronal (somatic) GCaMP6 brightness of labeled neurons in various transgenic and AAV infected mice. NIH line 10 is a *Thy1*-transgenic mouse line expressing GCaMP3 [38]. Ai38 is a Cre-dependent reporter mouse expressing GCaMP3 in the ROSA26 locus [12], here injected with *synapsin1*-Cre AAV. Each box indicates the 25^th^ to 75^th^ percentile distribution with different colors for each brain region, red line indicates the median, and whisker length is 150% of the 25^th^ to 75^th^ percentile distance, or until it touches the last sample position. Outliers are marked in red crosses. **b**, L2/3 pyramidal cells (86–289 cells per line; median, 181). **c**, L5 pyramidal cells (45–230 cells per line; median, 148). **d**, Hippocampal pyramidal cells (25–342 cells per line; median, 113). **e**. Confocal image of GP5.17 fixed tissue (green) counterstained with NeuN (red). **f–h**. Fraction of neurons that are GCaMP6-positive, estimated by counterstaining with NeuN, corresponding to **b–d**, respectively (54–186 cells per line; median, 102).

We quantified expression across brain regions (Table 1; Data S1) and individual neurons by quantifying somatic native fluorescence in fixed tissue (Fig. 3a). The highest expression was typically seen in the hippocampus. For cortical regions, expression in layers 5 and 6 was usually higher than for layer 2/3, whereas layer 4 cells did not express (Fig. 1b, c, Fig. 3a–d), consistent with other *Thy1* transgenic mice [20]. Expression was detected in multiple other brain regions (Table 1; Data S1).

Expression patterns varied across lines. For instance, distinct cortical regions expressed GECIs and different fractions of cells were labeled in these brain regions (Fig. 3). For some lines, *e.g.* GP4.3 and GP5.11, the majority of pyramidal cells in a particular cortical region were labeled. For other lines, *e.g.* GP5.17 and GP5.5, only a minor population of the cells was labeled (Fig. 3e–h). Expression levels were generally lower than seen with AAV infection. However, in some brain regions GP4.12 and GP5.17 lines showed expression levels comparable to AAV infection (Fig. 3b, c).

*Thy1* transgenics can exhibit transgene expression in specific subsets of cortical projection neurons [20]. In GP5.3 mice L2, but not L3 cells, were labeled over large parts of the cortex; in the neocortex of GP4.7 mice mainly L6 neurons were labeled; in GP4.9 mice mainly L5a cells were labeled; GP4.14 shows sparse labeling of cells in multiple cortical regions (Data S1).

### Imaging activity in the visual cortex *in vivo*

**Figure 4:**
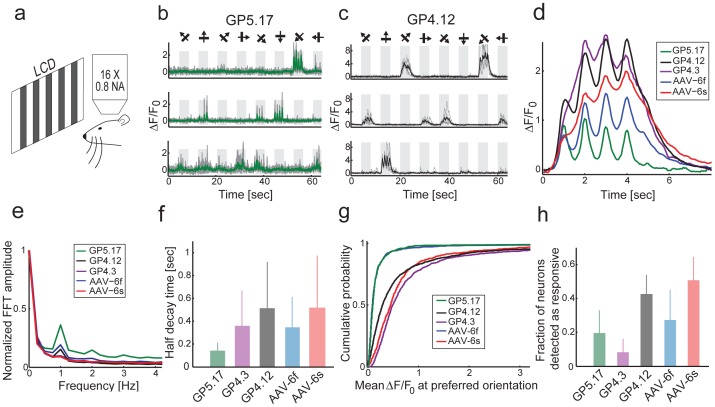
Functional imaging in the visual cortex (V1) of transgenic and AAV infected mice. **a**. Schematic of the experimental setup. **b**. Responses of three GP5.17 example cells to eight oriented grating stimuli. **c**. Responses of three GP4.12 example cells. **d**. Mean ΔF/F_0_ responses to the preferred stimulus for all cells with peak ΔF/F_0_>1. Cells were aligned according to their response maximum to one of four time points (1, 2, 3, or 4 s), and each stimulus lasts 4 s (average of 91 cells for GP5.17, 124 cells for GP4.3, 362 cells for GP4.12, 83 cells for AAV-6f, and 224 cells for AAV-6s). **e**. Fourier transform of the response to the preferred stimulus (median across cells). The 1 Hz peak corresponds to the frequency of the drifting grating. **f**. Half-decay time (mean±s.d.) after the last response peak during stimulus presentation (n = 52, 75, 446, 136, and 235 cells for GP5.17, GP4.3, GP4.12, AAV-6f, and AAV-6s respectively). **g**. Distribution of ΔF/F_0_ responses to the preferred stimulus. **h**. Fraction of statistically significant responsive cells (mean±s.d., n = 731,1130, 1325, 871, and 672 cells, for GP5.17, GP4.3, GP4.12, AAV-6f, and AAV-6s respectively).

We next performed *in vivo* functional imaging in L2/3 of the primary visual cortex of transgenic mice (Fig. 4a). Three transgenic lines were tested: GP4.3 with moderate expression levels and a majority of L2/3 pyramidal neurons labeled; GP4.12 with higher expression levels and ∼45% of L2/3 pyramidal neurons labeled; GP5.17 with the highest expression levels and ∼30% of L2/3 pyramidal neurons labeled (Fig. 3f). Anesthetized mice were presented with oriented gratings moving in eight different directions (Fig. 4a, Methods) [7].

Subsets of GCaMP6 positive cells showed tuned responses to the stimulus (Fig. 4b–d). A majority of the responsive neurons were modulated at the temporal frequency of the moving grating (1 Hz). GP5.17 showed stronger modulation at 1 Hz than GP4.3 and GP4.12, presumably due to the faster kinetics of GCaMP6f vs. GCaMP6s [7]. The transgenic lines showed stronger or similar modulation than AAV-infected mice with the same indicator (Fig. 4d, e).

We analyzed the half-decay time of fluorescence traces after the last response peak during stimulus presentation (Fig. 4f, Methods). The averaged half-decay time was faster for the GP lines than for AAV infected mice (GP5.17, 140±70 ms, n = 52; versus AAV-GCaMP6f, 350±300 ms, n = 136; mean±s.d.) (GP4.3, 360±300 ms, n = 75; GP4.12, 510±400 ms, n = 446; versus AAV-GCaMP6s, 510±460 ms, n = 235). Line GP5.17 shows the fastest responses measured, with decay times in the 150 ms range, close to the decay time expected for cytoplasmic calcium after an action potential [35]. This faster observed kinetics may be associated with lower GECI concentration, consistent with previous experiments [35], [36].

Response amplitudes of GP4.3 and GP4.12 were higher than for GP5.17 (Fig. 4b–d, g), as expected from the higher sensitivity of GCaMP6s vs. GCaMP6f [7]. The percentage of L2/3 cells detected as responding varied across the different lines. For the highly expressing GCaMP6s line the fraction was similar to AAV expression (GP4.12, 42.7±11.1%; 2 mice, 33 FOVs, 1325 cells vs. AAV-6s, 50.9±13.7%; 3 mice, 23 FOVs, 672 cells). Similarly, for the highly expressing GCaMP6f line the fraction was also comparable to AAV expression (GP5.17, 19.5±13.7%; 3 mice, 32 FOVs, 731 cells vs. AAV-6f, 27.5±17.7%; 3 mice, 29 FOVs, 871 cells). For the lower expressing GCaMP6s line the fractions were lower (GP4.3, 8.3±7.9%; 2 mice, 19 FOVs, 1130 cells), probably because of low fluorescence signal and reduced signal-to-noise ratio, SNR (Fig. 4h).

### Imaging activity during behavior

**Figure 5:**
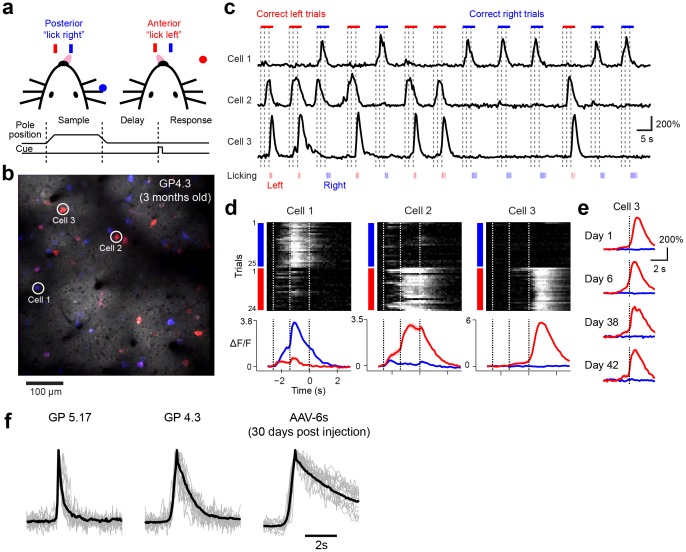
ALM functional imaging using GP4.3 during behavior. **a**. Schematic of the object localization behavior (see ref. [34] for details). **b**. An image of GCaMP6s labeled neurons in a GP4.3 mouse, 160 µm below the pia. Blue and red colors indicate cells that responded during lick-right and lick-left trials, respectively. Cue = auditory “go” signal. **c**. Fluorescent responses of three example cells indicated in **b**. **d**. Top, single-trial responses of the same neurons in **b** sorted according to trial type (blue: lick-right, red: lick-left). Bottom, trial-averaged response. **e**. The responses of cell 3 measured over 4 behavioral sessions spanning more than one month. **f**. Peak normalized fluorescent transients of GCaMP expressing neurons in GP5.17, GP4.3 and AAV-GCaMP6s injected mice (0.2±0.1 s, 0.65±0.35 s, and 1.8±1.1s mean±s.d; n = 207, 771, and 369 cells for GP5.17, GP4.3, and AAV-6s respectively).

We trained head-fixed GP4.3 mice in a whisker-based object location discrimination task [34], [37]. In each trial, a vertical pole was presented in one of two positions (anterior or posterior) during a sample epoch (1.3 s) (Fig. 5a, b). Mice learned to discriminate the location of the pole using their whiskers. During a subsequent delay epoch (1.3 s) mice prepared for the upcoming response. An auditory “go” cue (0.1 s) signaled the beginning of the response epoch, when mice reported the perceived pole position by licking one of two lickports (posterior→“lick right”, anterior→“lick left”) (Fig. 5a). Mice achieved high levels of performance (mean percent correct>70%). Imaging was performed in the anterior lateral motor cortex (ALM), which is known to be involved in planning and execution of voluntary licking [33], [34]. Consistent with histological data (Fig. 2, 3), we observed densely labeled GCaMP6s expressing neurons in L2/3 (Fig. 5b). Neurons were active during specific periods of the trial and for specific licking directions (Fig. 5c). The stable expression level allowed us to image the same cells over times of many weeks. Direction selective cells showed consistent responses (Fig. 5e). The half-decay time constant of calcium transients was 0.65±0.35 s (mean±s.d., n = 127, Fig. 5f), significantly faster than GCaMP6s expressed using AAV (1.8±1.1s, n = 84, p<10^−23^, t-test). We conclude that GP mice are suitable for long-term mapping of behavior-related neuronal dynamics across large cortical areas.

## Discussion

We generated multiple transgenic mouse lines with stable and reproducible expression of GCaMP6s and GCaMP6f under the control of the *Thy1* promoter (‘*Thy1*-GCaMP6’ lines). Each line has a unique expression pattern, a hallmark of *Thy1* transgenics [20], [22]. Expression was distributed across numerous brain regions and cell types. Selected lines showed sufficient expression levels for cellular *in vivo* imaging with good signal-to-noise ratio, obviating the need for AAV injection and the associated surgery. GCaMP6 expression was stable across many months, without signs of cytotoxicity. The sensitivity and kinetics of GCaMP6s and GCaMP6f make the *Thy1*-GCaMP6 mice a preferred choice for long-term cellular imaging of neuronal populations in the intact brain.

We characterized GCaMP expression and functional signals in the *Thy1*-GCaMP6 mice and compared them to previously published transgenic mice and AAV infected mice (Table 1, Fig. S1 and S2, Data S1). Neocortical expression levels in *Thy1*-GCaMP6 mice are 2–10 fold lower than typical conditions of AAV infection (Fig. 3). Since AAV infection produces [GCaMP] of approximately 80 µM [1], [12], we estimate that expression levels in the *Thy1* mice are on the order of 8–40 µM. The *Thy1*-GCaMP6 mice showed higher cortical expression levels than two GCaMP3 transgenic lines, Ai38 [12] and NIH line 10 [38]. Two lines, GP4.12 and GP5.17, showed comparable expression levels to AAV infected animals in selected cortical regions. No signs of cytomorbidity (*e.g.* nuclear filling of cells) was observed in any of the GP transgenic lines, even after many months of expression.

We tested *Thy1*-GCaMP6 mice for *in vivo* imaging in anesthetized and awake, behaving mice. We observed robust signals with faster kinetics compared to AAV infected mice (Fig. 4d,f, Fig. 5f). This enhanced speed may be explained by the lower concentration of GCaMP6 in the *Thy1*-GCaMP6 mice (Fig. 3b, c) and weaker calcium buffering [35], [36]. In *Thy1*-GCaMP6 mice expression was stable over time (Fig. 2). Maintaining stable expression level over time is challenging with AAV-mediated expression [1]. AP detection following visual stimulation was similar to AAV infected mice, *i.e.* superior to Oregon Green BAPTA-1 [7].

There are several drawbacks in using *Thy1*-GCaMP6 mice. First, the lower expression levels (Fig. 3) require higher laser power (∼50%–100% higher). However, we did not observe laser-induced damage in brain tissue even after multiple imaging sessions involving continuous imaging over one hour. Photobleaching was negligible. Second, *Thy1*-GCaMP6 mice show mosaic expression, unevenly distributed across different brain regions and cortical layers. Experiments performed across multiple brain areas may require different transgenic mouse lines. Third, the *Thy1* promoter drives expression mostly in projection neurons. Other types of neurons, including GABAergic neurons, are not accessible using this strategy.

### Availability

Lines GP 4.3, 4.12, 5.5, 5.11, and 5.17 are available at Jackson Laboratories (http://jaxmice.jax.org), with stock numbers 024275, 025776, 024276, 024339, and 025393, respectively. For other lines please contact the GENIE project (kimd@janelia.hhmi.org). AAV viruses are available at the University of Pennsylvania Vector Core (http://www.med.upenn.edu/gtp/vectorcore/Catalogue.shtml).

## Supporting Information

Figure S1 **AAV-mediated expression** ***vs.*** **transgenic expression.** Images of fixed tissue coronal sections of AAV mediated expression (GCaMP6s, left image) and transgenic expression (GP4.12, right image). For the AAV-injected mouse, two injections (25 nl each, *synapsin1*-AAV GCaMP6s) were made in adjacent locations (0.4 mm) in mouse V1, resulting in typical inhomogeneous expression with several nuclear-filled cells (imaged 4 weeks after the AAV injection). The transgenic GCaMP expression shows no filled cells (P56).
(TIF)

Figure S2 **Quantification of GCaMP expression for multiple GP lines.** Somatic GCaMP6 brightness of labeled neurons in various transgenic GP lines. For 5 lines (GP4.3, GP4.12, GP5.5, GP5.11, and GP5.17) more than one mouse was analyzed, and GCaMP brightness for each individual animal is presented (*i.e.* GP4.3A, GP4.3B, *etc.*). Somatic brightness distribution for GP4.x (upper row), GP5.x (lower row), layer 2/3 cells (left column) and layer 5 cells (right column) is shown. Each box indicates the 25^th^ to 75^th^ percentile distribution in different colors for each brain region, red line indicates the median, and whisker length is 150% of the 25^th^ to 75^th^ percentile distance, or until it touches the last sample position. Outliers are marked in red crosses.
(TIF)

Figure S3 **GCaMP6 expression in the olfactory bulb.** Confocal microscope images of fixed coronal sections show different expression patterns in the olfactory bulb. Mitral cells are labeled in GP4.3 and GP5.11 lines, whereas lines GP4.12, GP5.5, and GP5.17 show brighter signal in the granule layer.
(TIF)

Data S1 Widefield images of GP lines coronal sections. Widefield microscopy images were taken using a slide scanner (Nanozoomer, Hamamatsu) with a ×20 0.75 NA air objective (Olympus). Imaging conditions were kept constant across time, but note the different greyscale range used for presenting the different lines. Sections thickness was 50 µm; every second sections was mounted and used for imaging (see Methods section for details). Sections were mounted from anterior to posterior. For several lines (such as GP4.2 and GP5.18) only a subset of sections were mounted.
(DOCX)
