# Supplementary figures and images for: Thy1-GCaMP6 Transgenic Mice for Neuronal Population Imaging In Vivo
Source: PLoS One. 2014 Sep 24;9(9):e108697. doi: 10.1371/journal.pone.0108697 (PMC4177405; doi:10.1371/journal.pone.0108697)

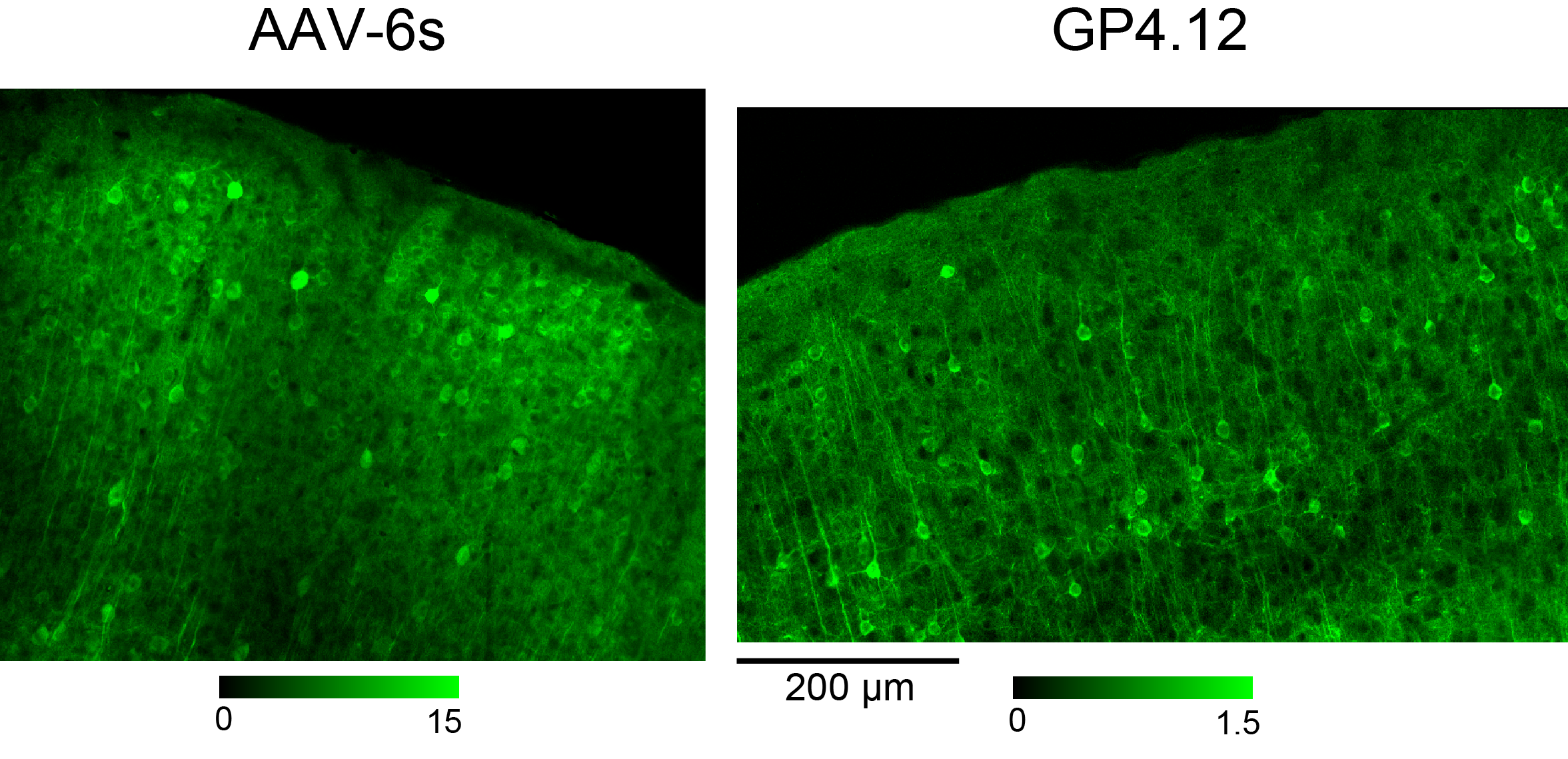

Supplement: Figure S1 — AAV-mediated expression vs. transgenic expression. Images of fixed tissue coronal sections of AAV mediated expression (GCaMP6s, left image) and transgenic expression (GP4.12, right image). For the AAV-injected mouse, two injections (25 nl each, synapsin1-AAV GCaMP6s) were made in adjacent locations (0.4 mm) in mouse V1, resulting in typical inhomogeneous expression with several nuclear-filled cells (imaged 4 weeks after the AAV injection). The transgenic GCaMP expression shows no filled cells (P56). (TIF) [file pone.0108697.s001.tif]

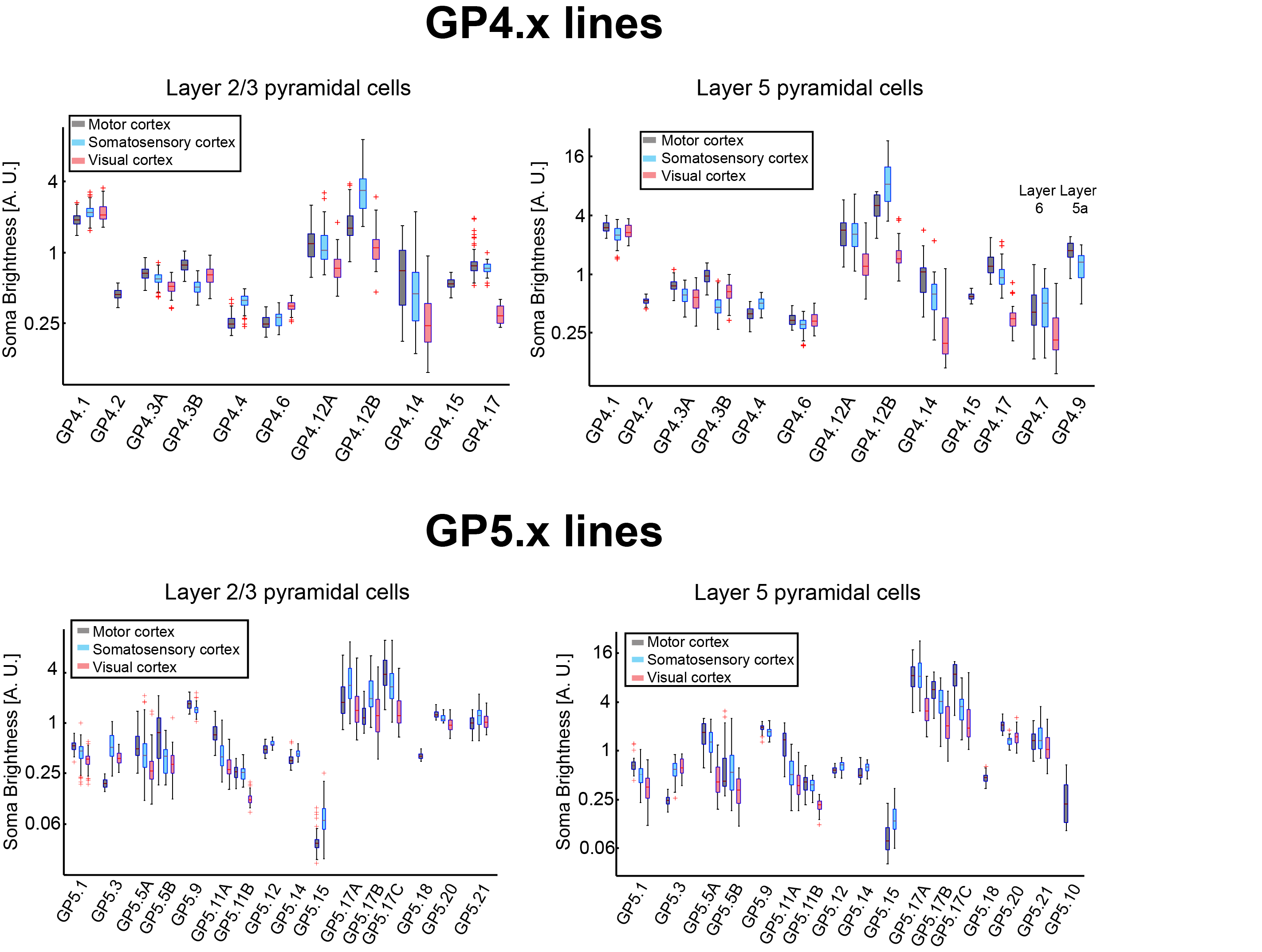

Supplement: Figure S2 — Quantification of GCaMP expression for multiple GP lines. Somatic GCaMP6 brightness of labeled neurons in various transgenic GP lines. For 5 lines (GP4.3, GP4.12, GP5.5, GP5.11, and GP5.17) more than one mouse was analyzed, and GCaMP brightness for each individual animal is presented (i.e. GP4.3A, GP4.3B, etc.). Somatic brightness distribution for GP4.x (upper row), GP5.x (lower row), layer 2/3 cells (left column) and layer 5 cells (right column) is shown. Each box indicates the 25th to 75th percentile distribution in different colors for each brain region, red line indicates the median, and whisker length is 150% of the 25th to 75th percentile distance, or until it touches the last sample position. Outliers are marked in red crosses. (TIF) [file pone.0108697.s002.tif]

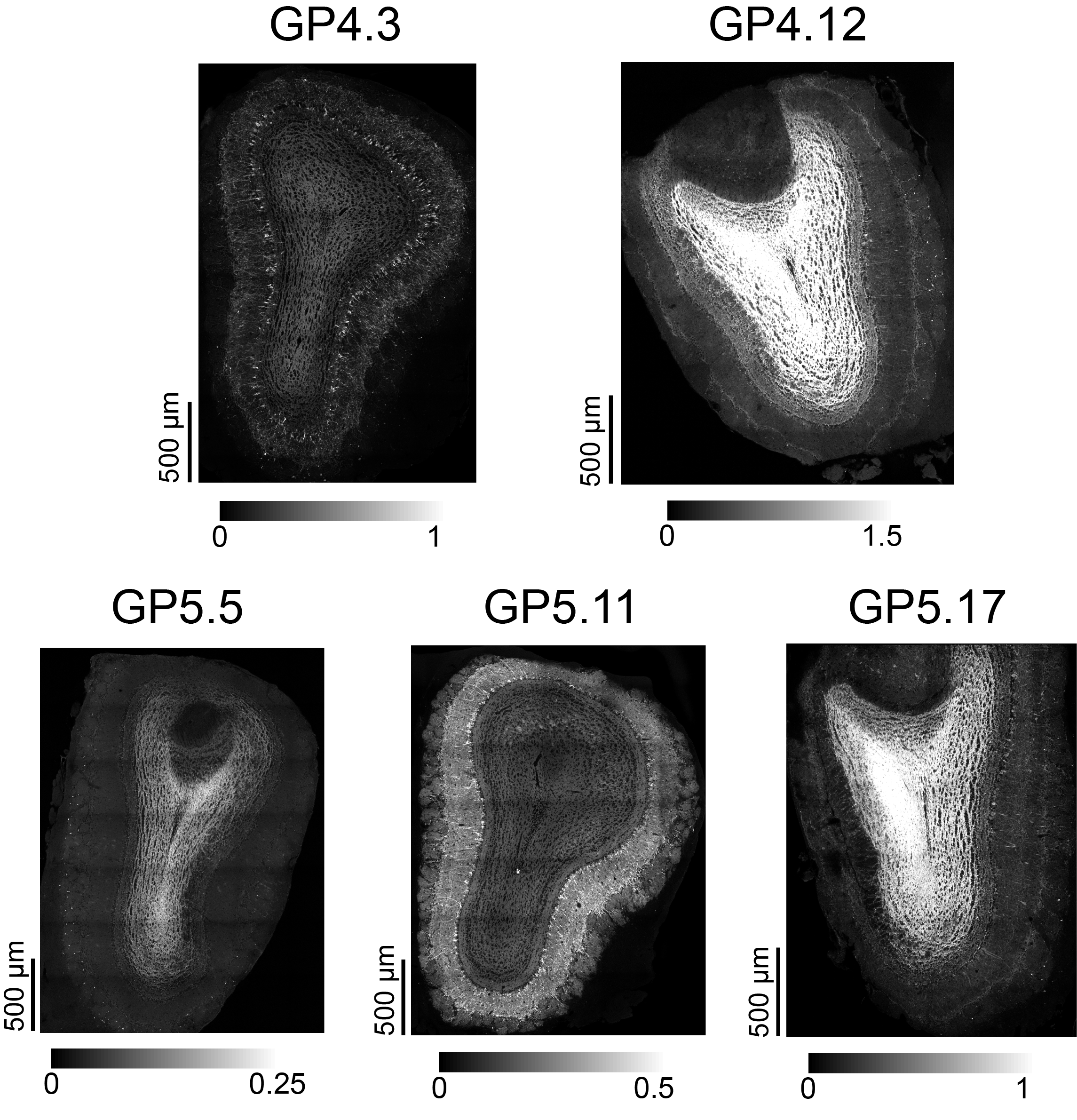

Supplement: Figure S3 — GCaMP6 expression in the olfactory bulb. Confocal microscope images of fixed coronal sections show different expression patterns in the olfactory bulb. Mitral cells are labeled in GP4.3 and GP5.11 lines, whereas lines GP4.12, GP5.5, and GP5.17 show brighter signal in the granule layer. (TIF) [file pone.0108697.s003.tif]
