# Supplementary material for: Thy1-GCaMP6 Transgenic Mice for Neuronal Population Imaging In Vivo
Source: PLoS One. 2014 Sep 24;9(9):e108697. doi: 10.1371/journal.pone.0108697 (PMC4177405; doi:10.1371/journal.pone.0108697)

**Widefield images of GP lines coronal sections**

Widefield microscopy images were taken using a slide scanner (Nanozoomer, Hamamatsu) with a x20 0.75 NA air objective (Olympus). Imaging conditions were kept constant across time, but note the different greyscale range used for presenting the different lines. Sections thickness was 50 μm; every second sections was mounted and used for imaging (see Methods section for details). Sections were mounted from anterior to posterior. For several lines (such as GP4.2 and GP5.18) only a subset of sections were mounted.


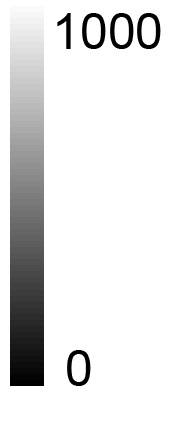

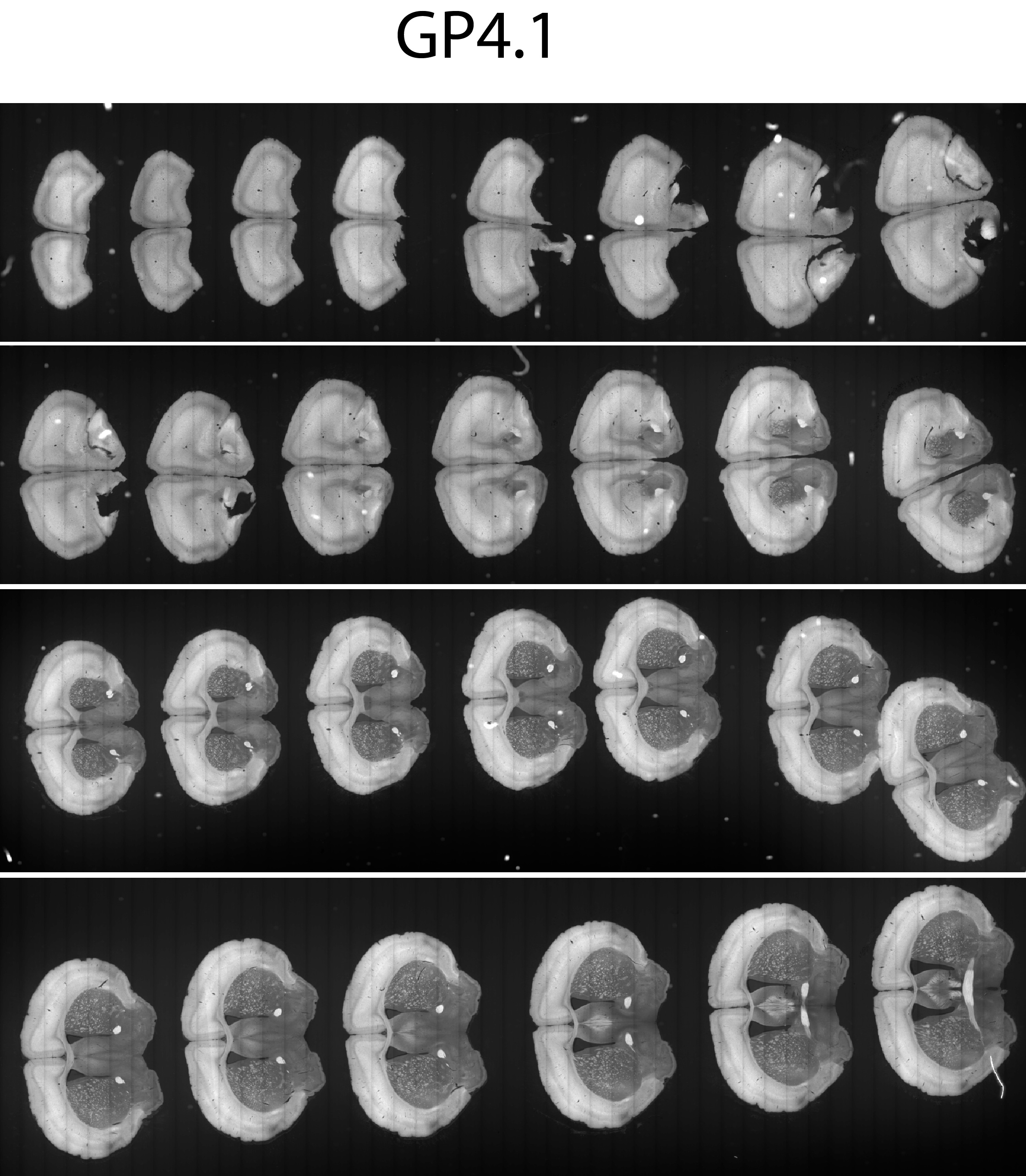


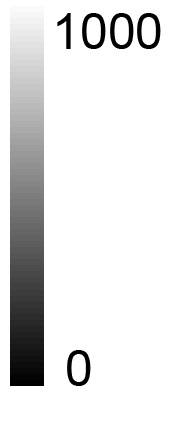

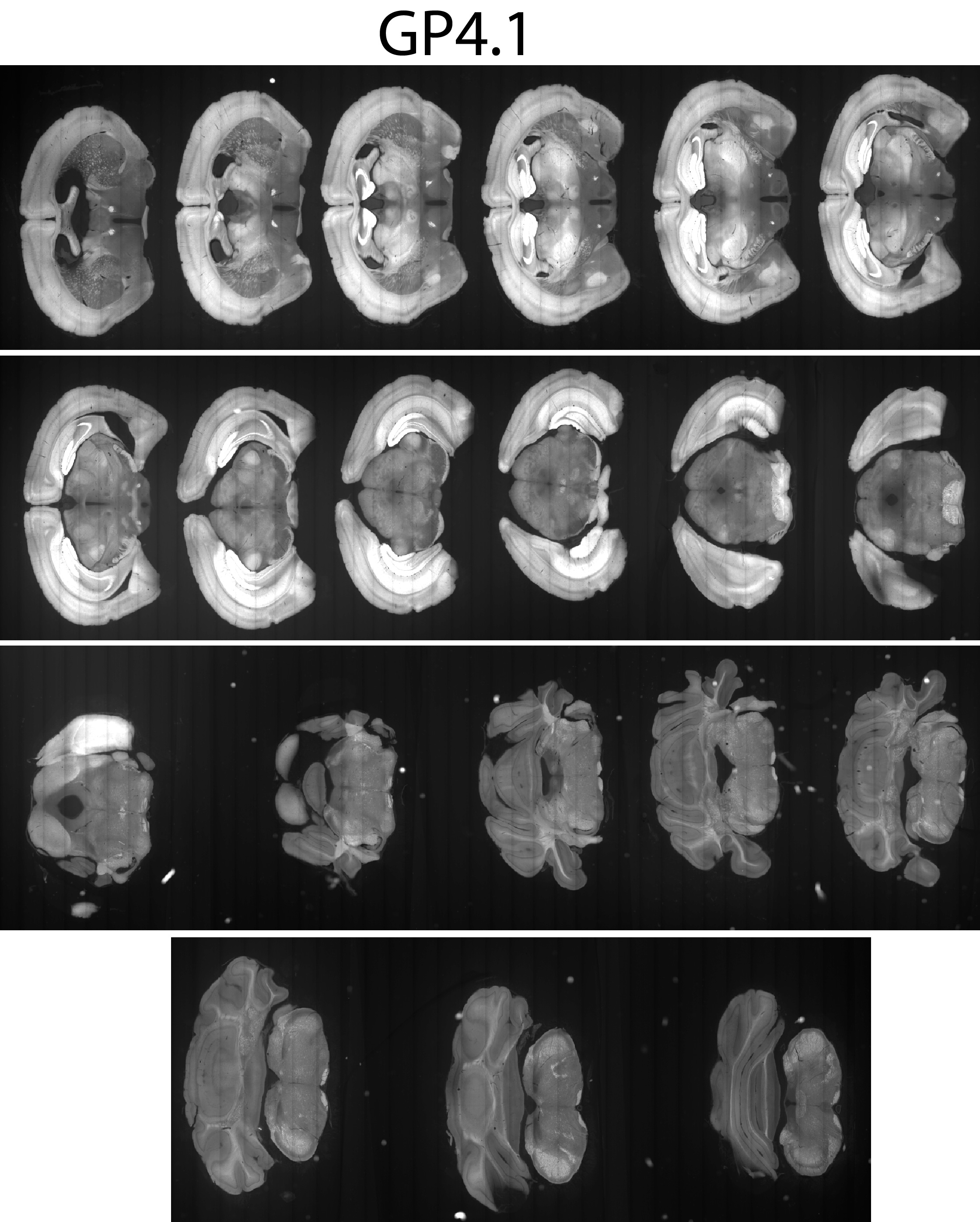


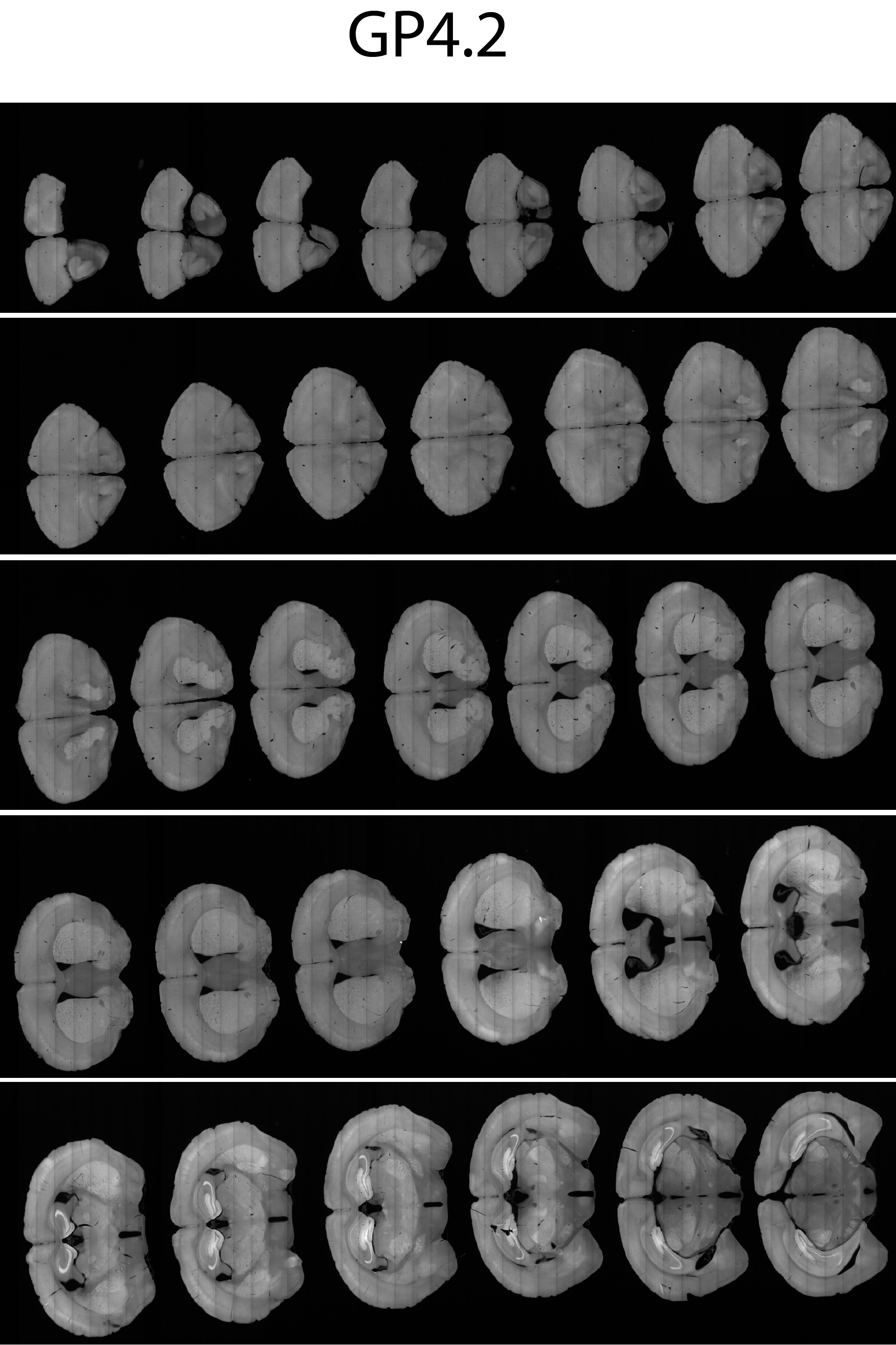

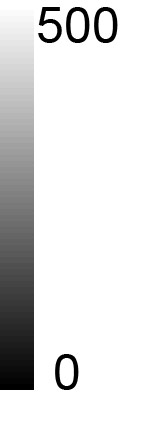


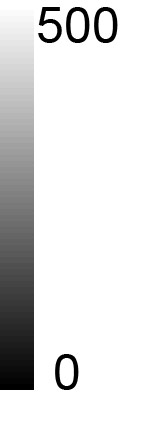

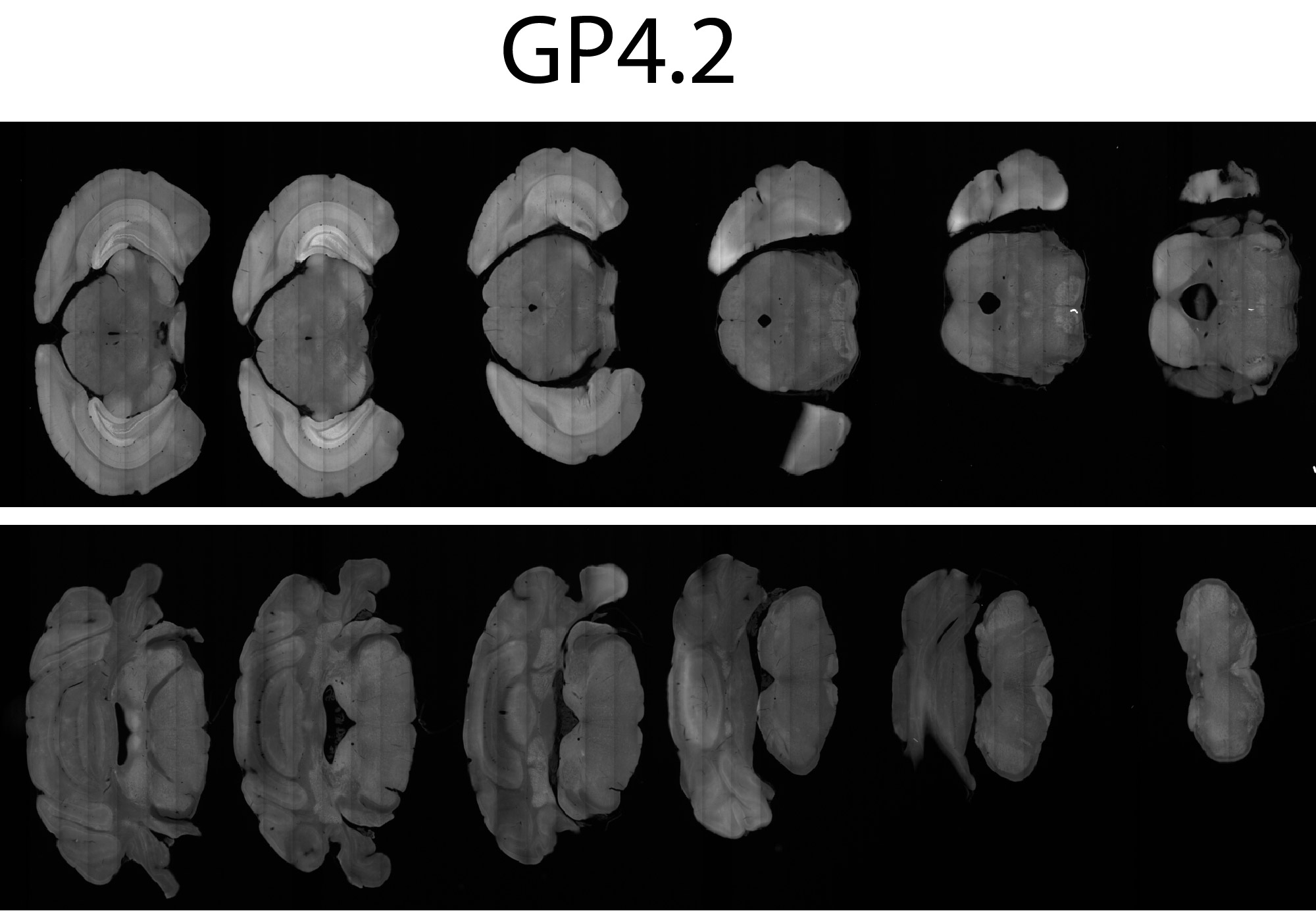


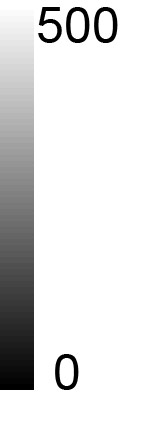

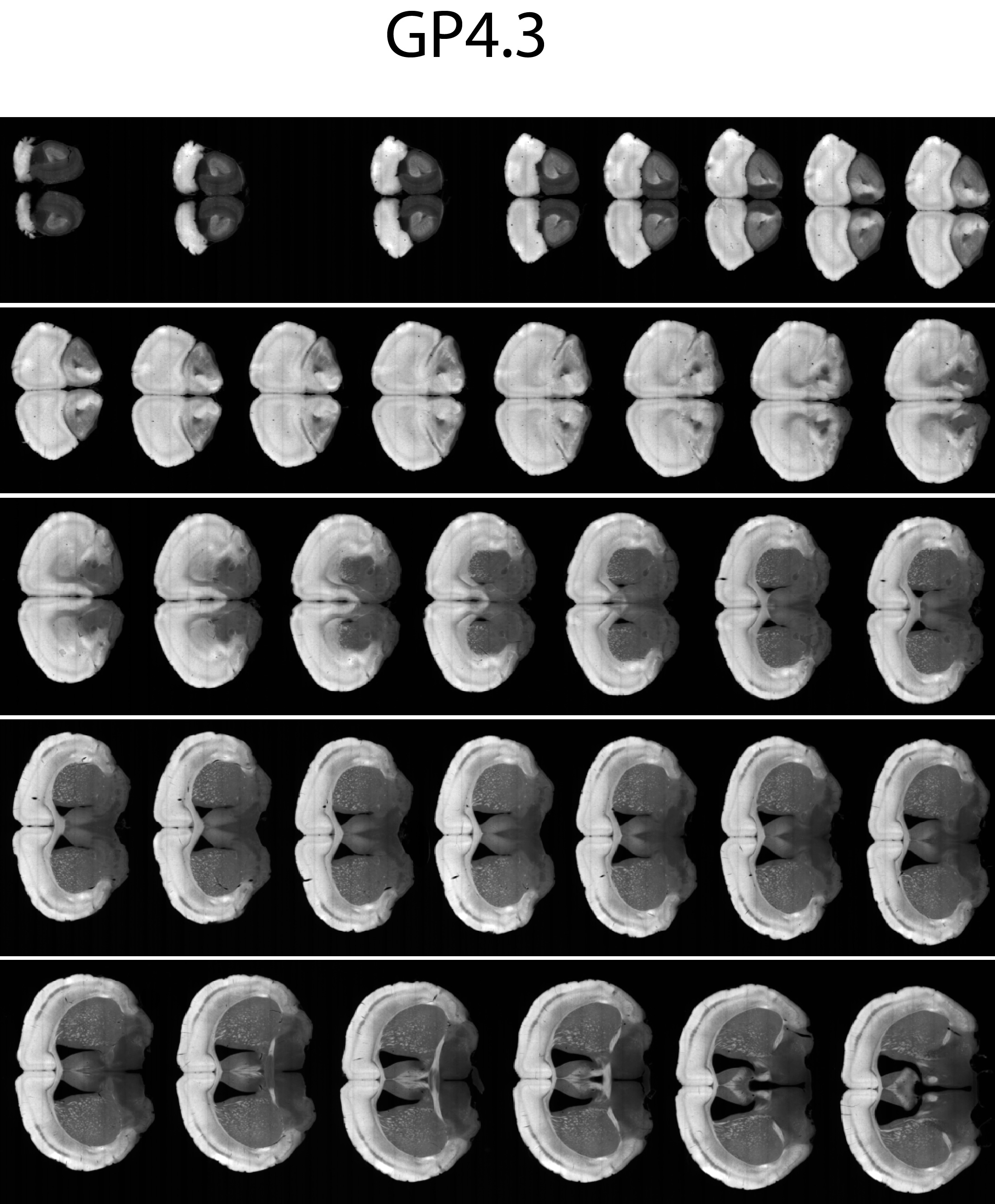


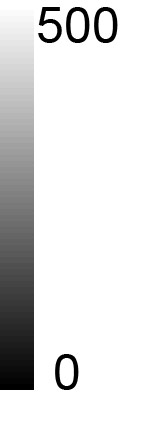

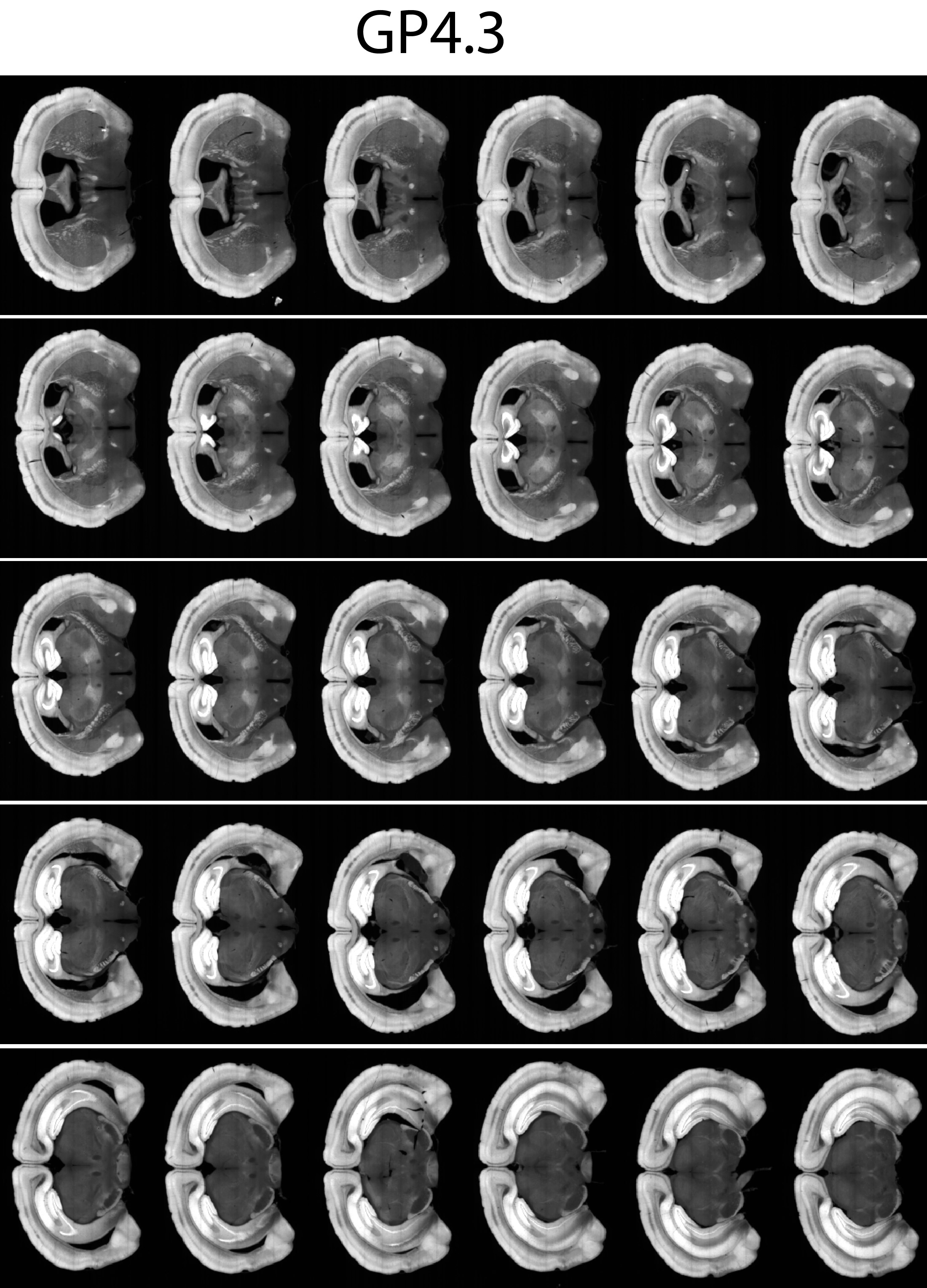


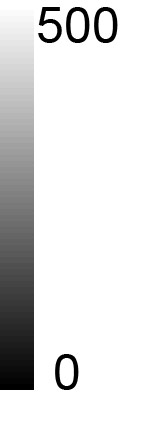

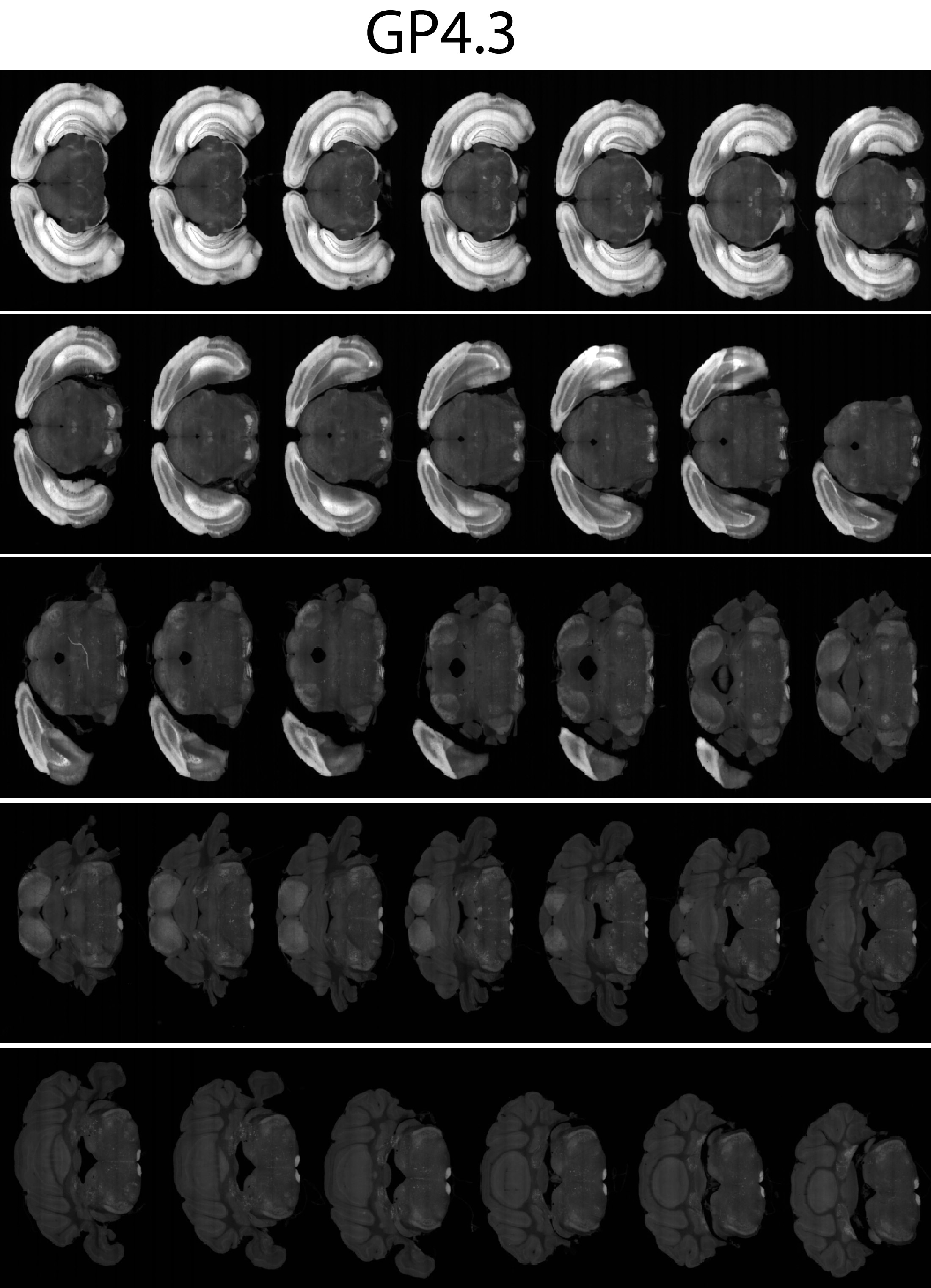


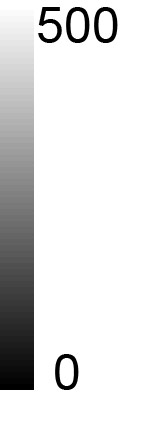

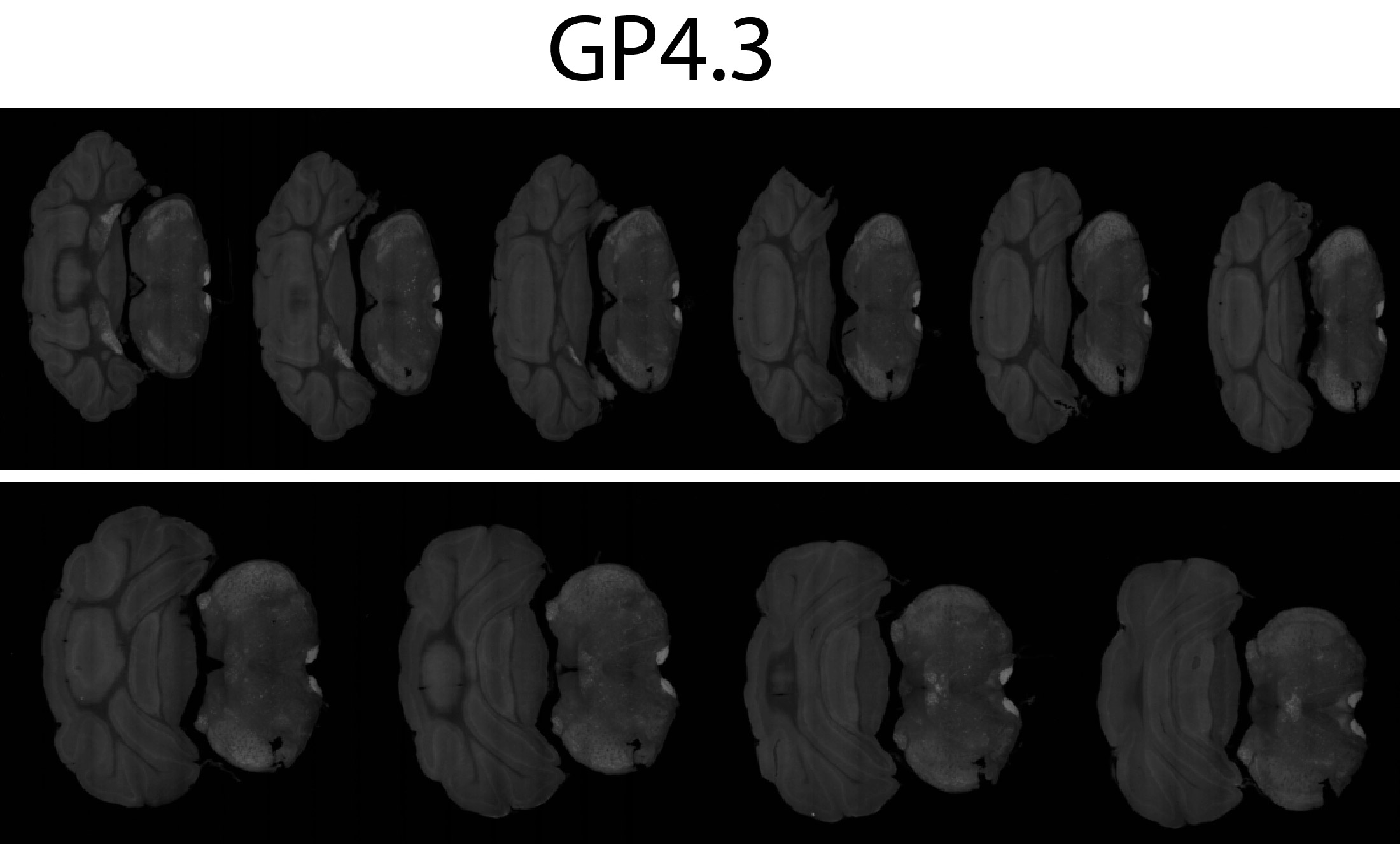


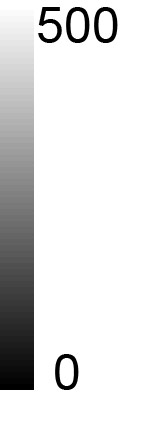

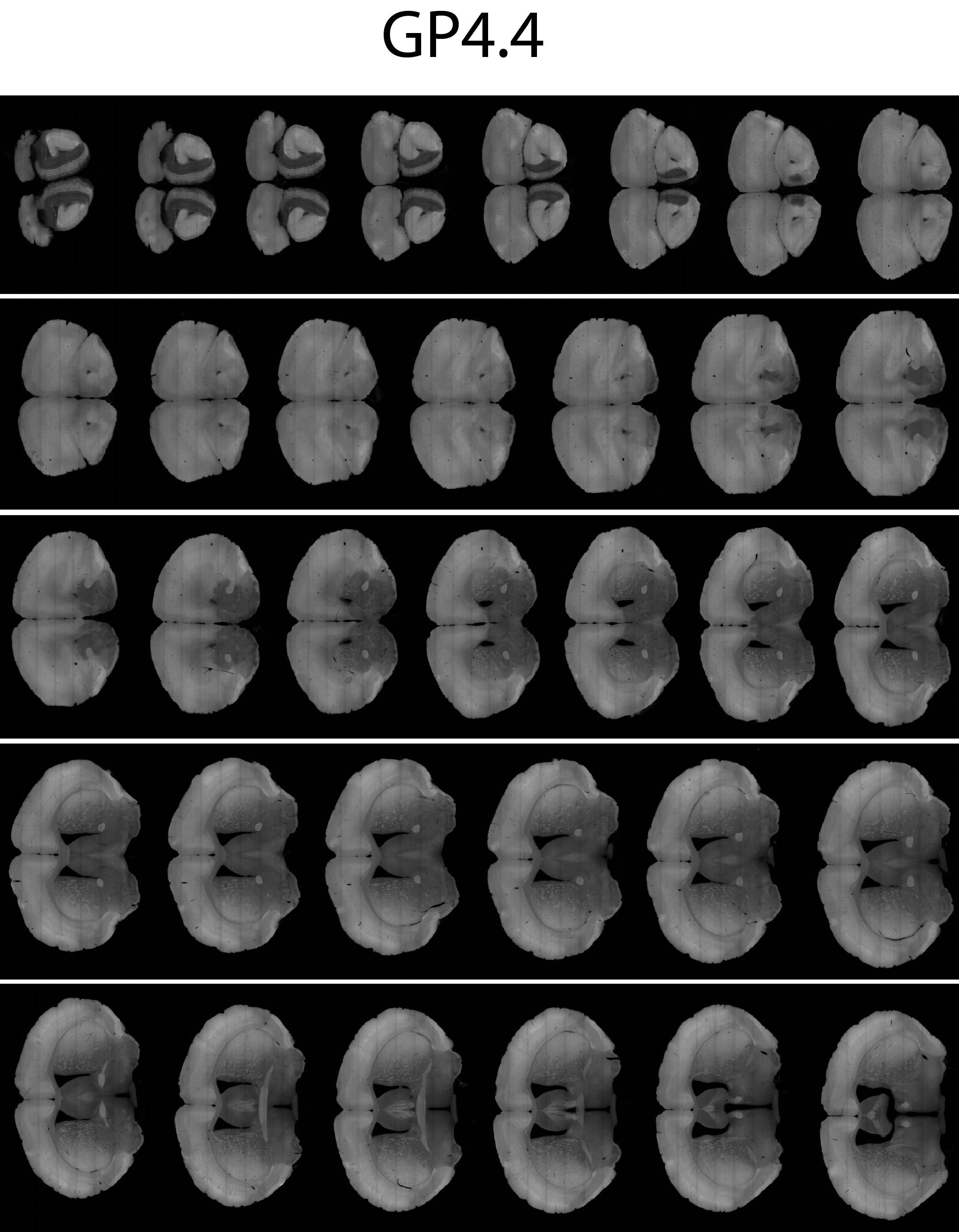


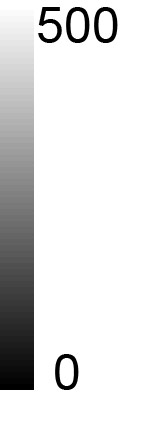

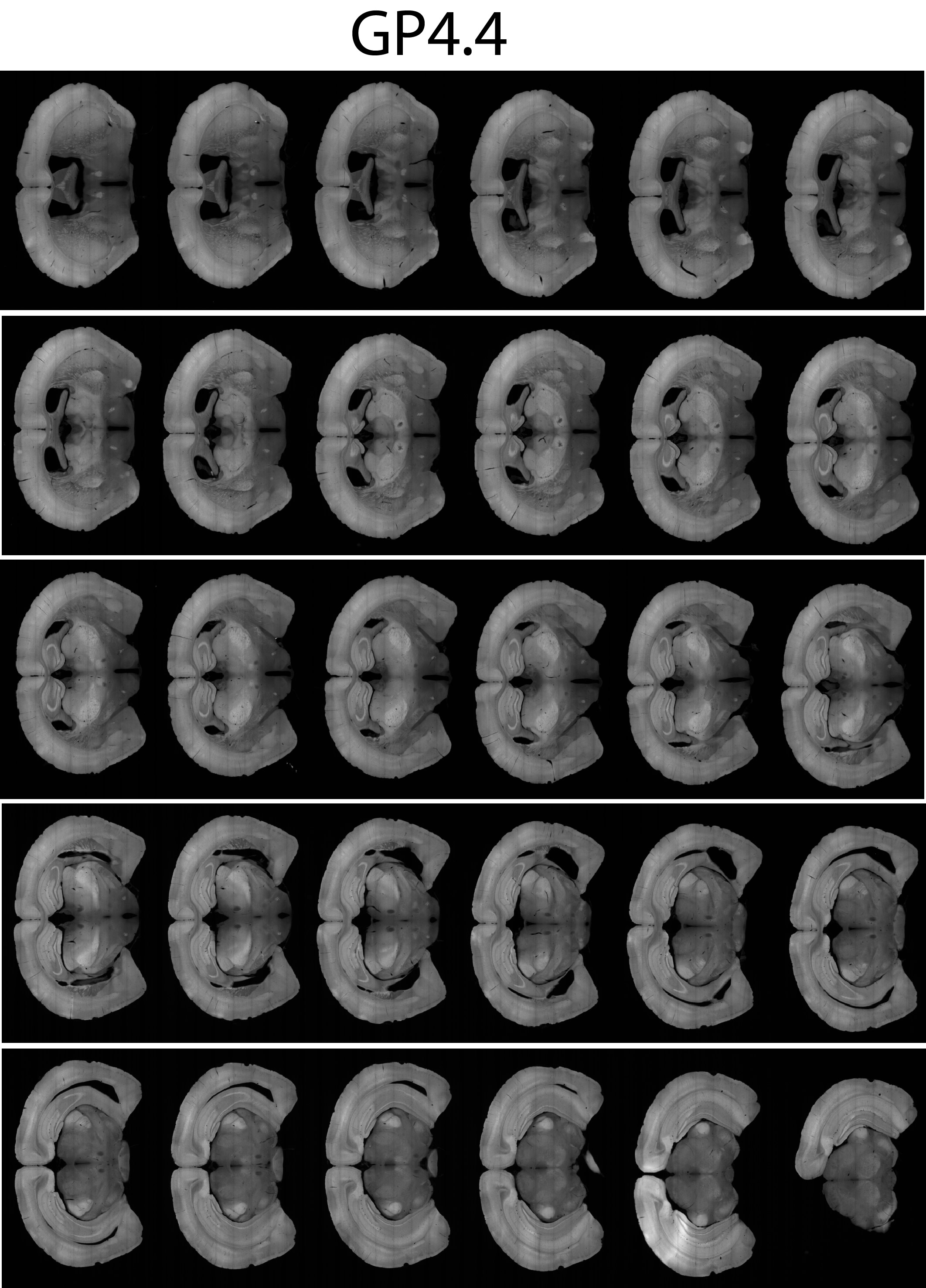


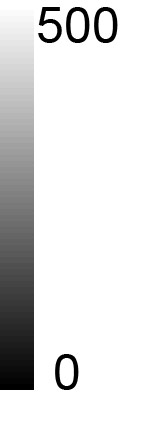

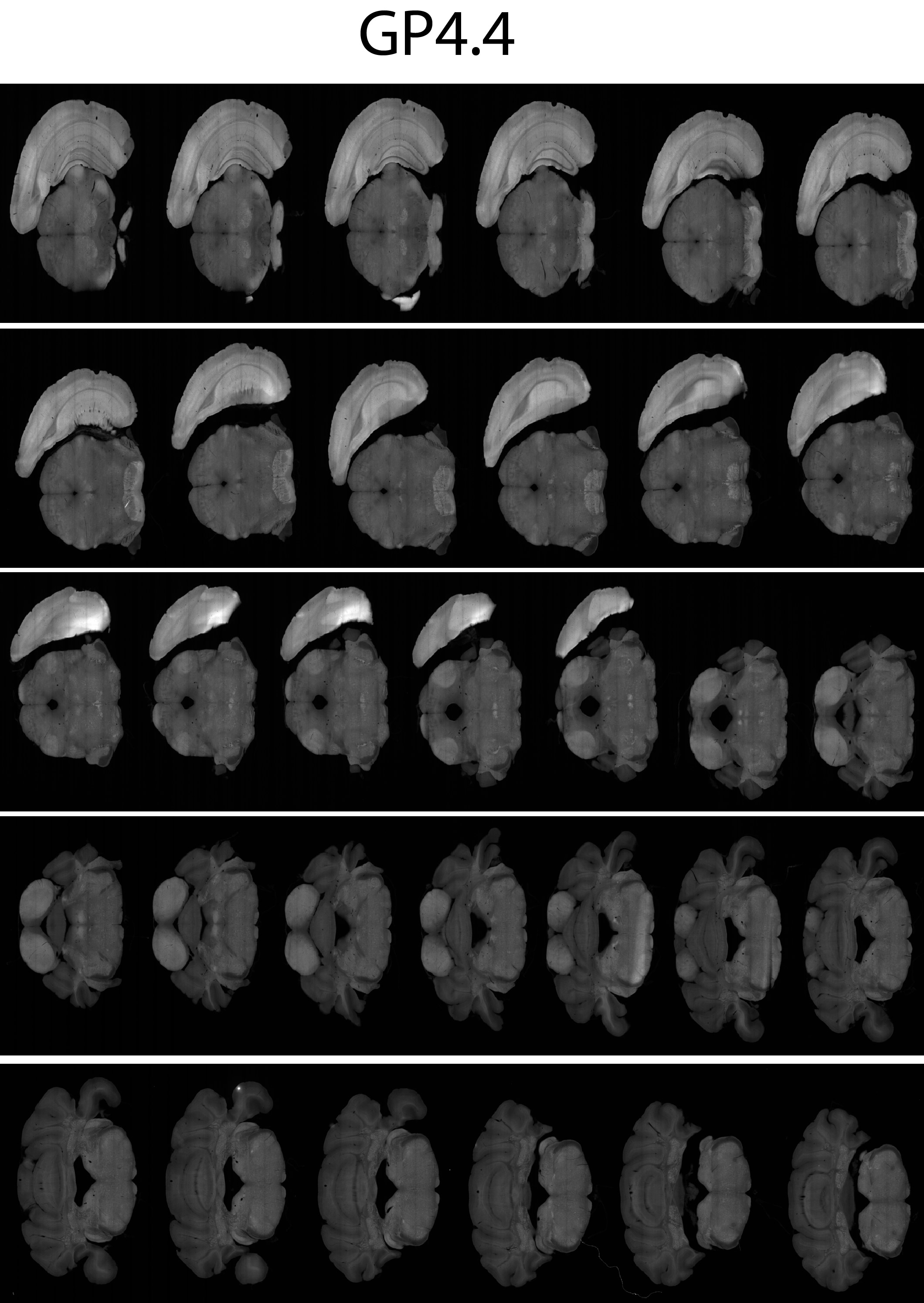


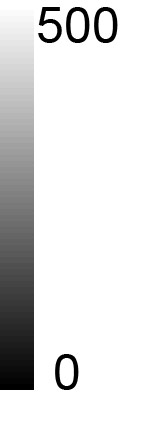

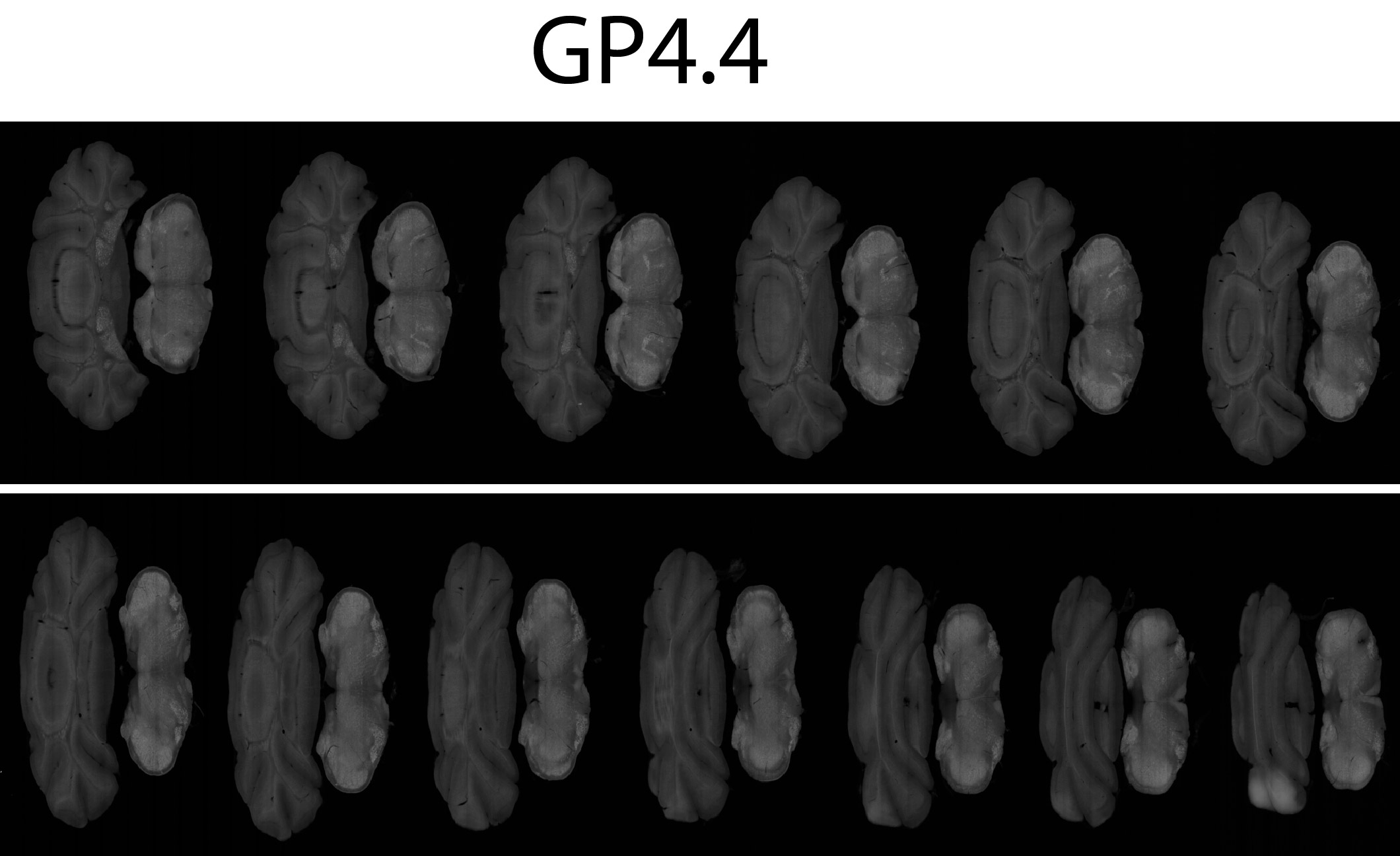


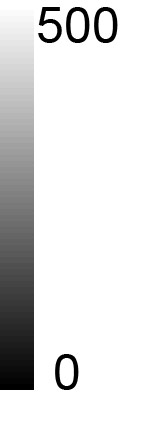

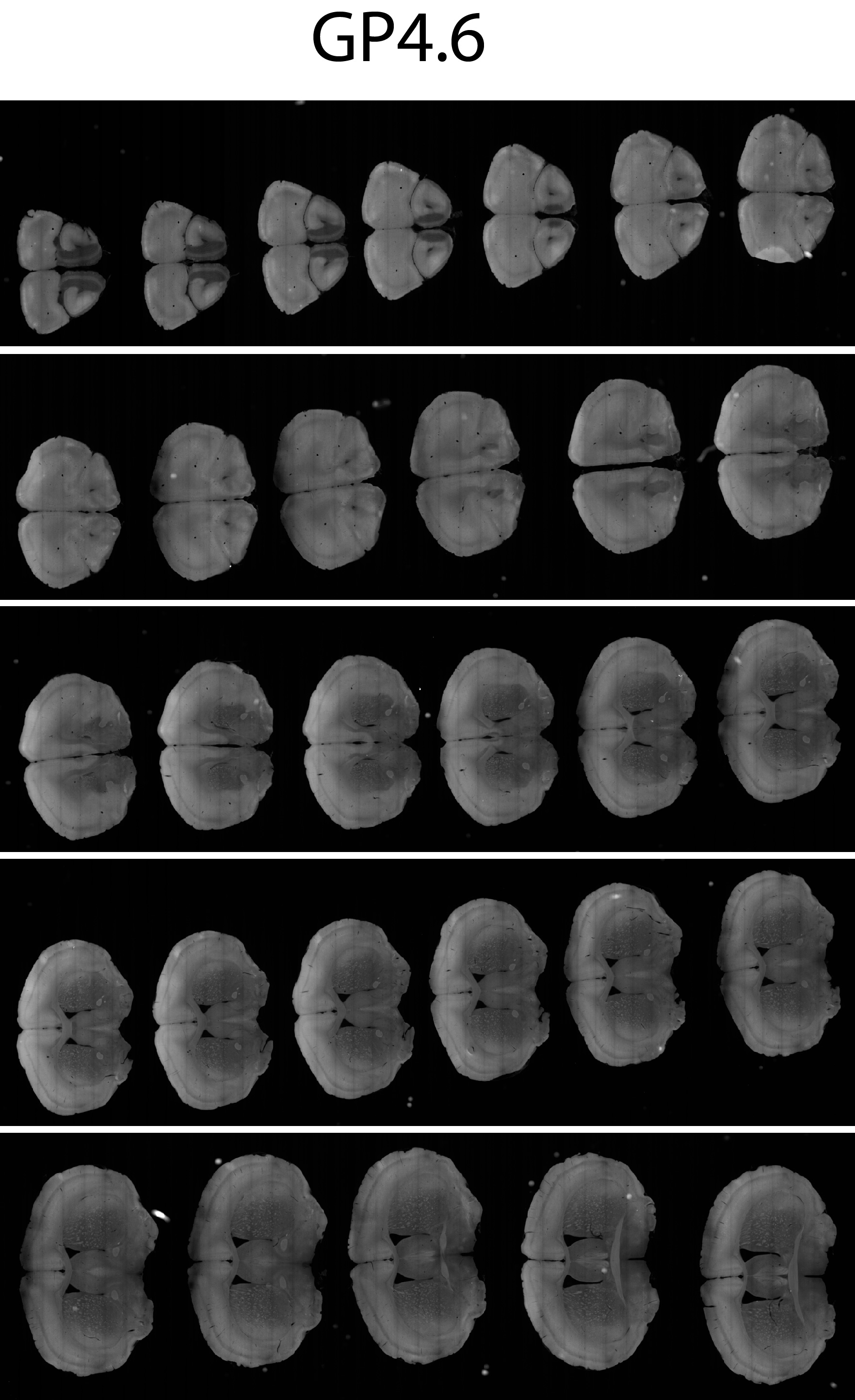


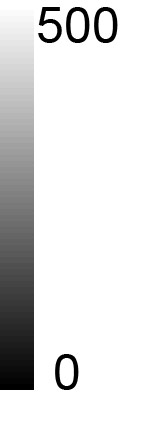

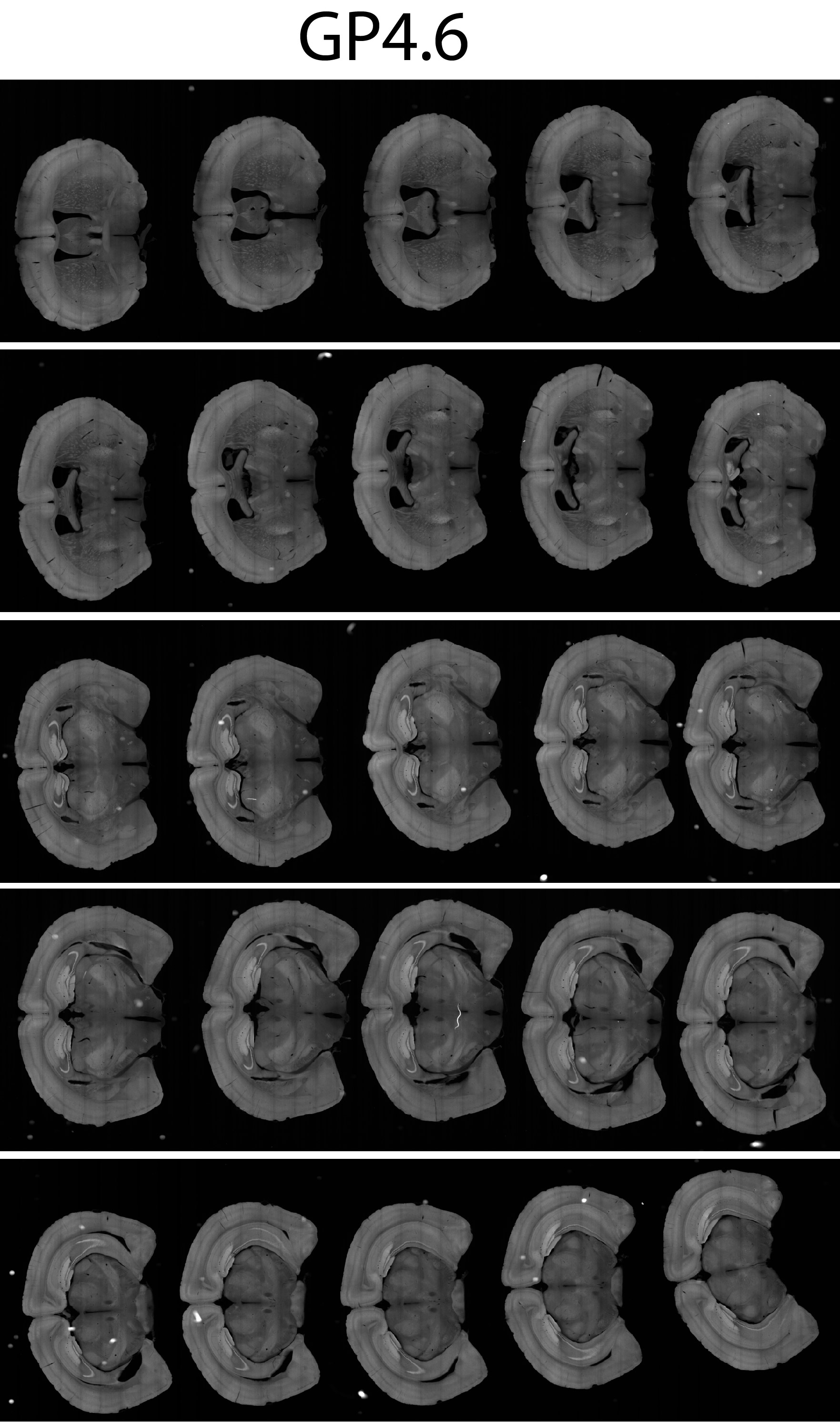


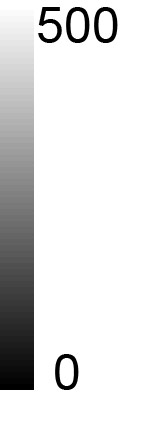

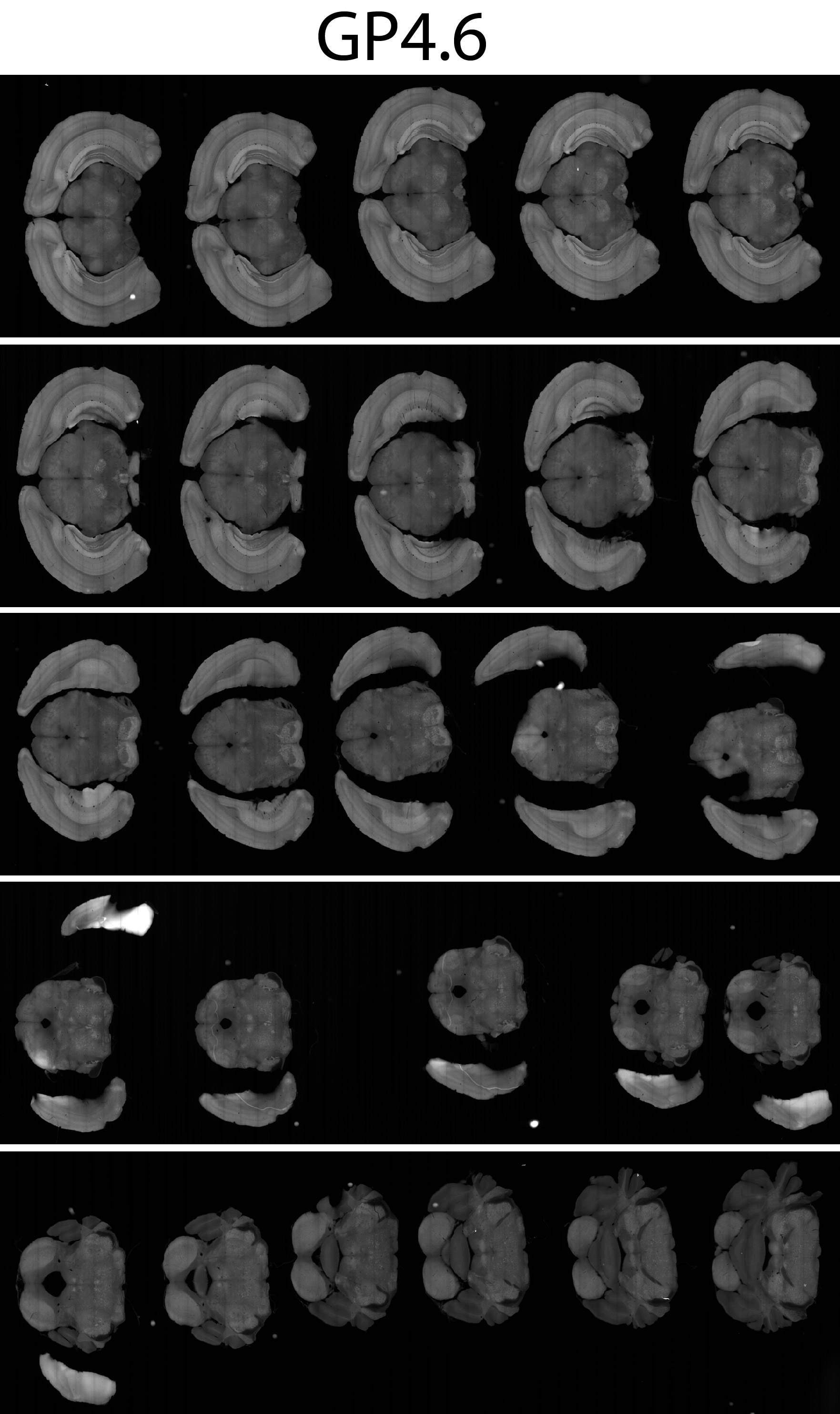


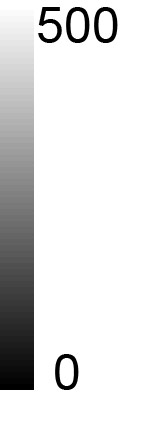

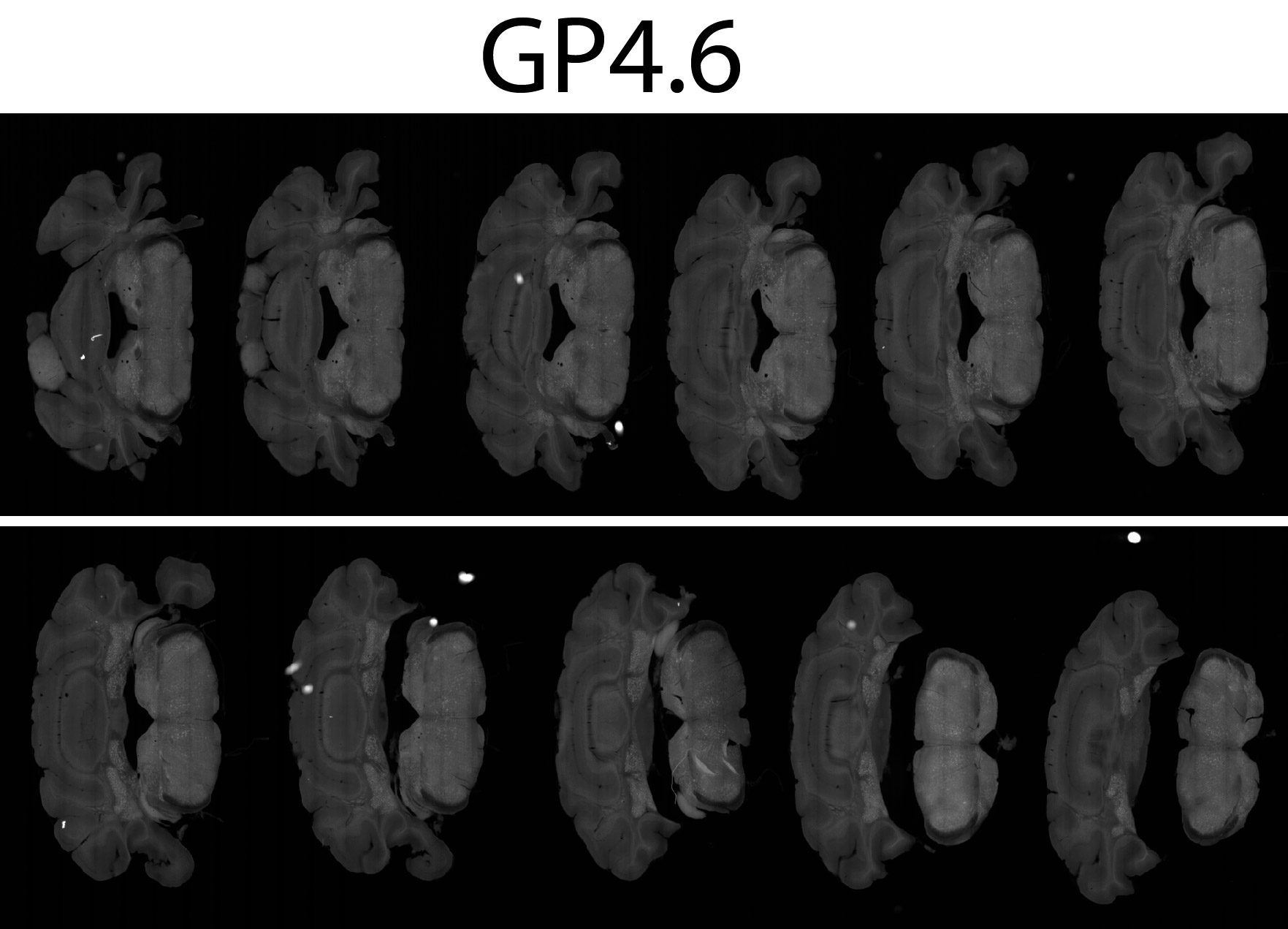


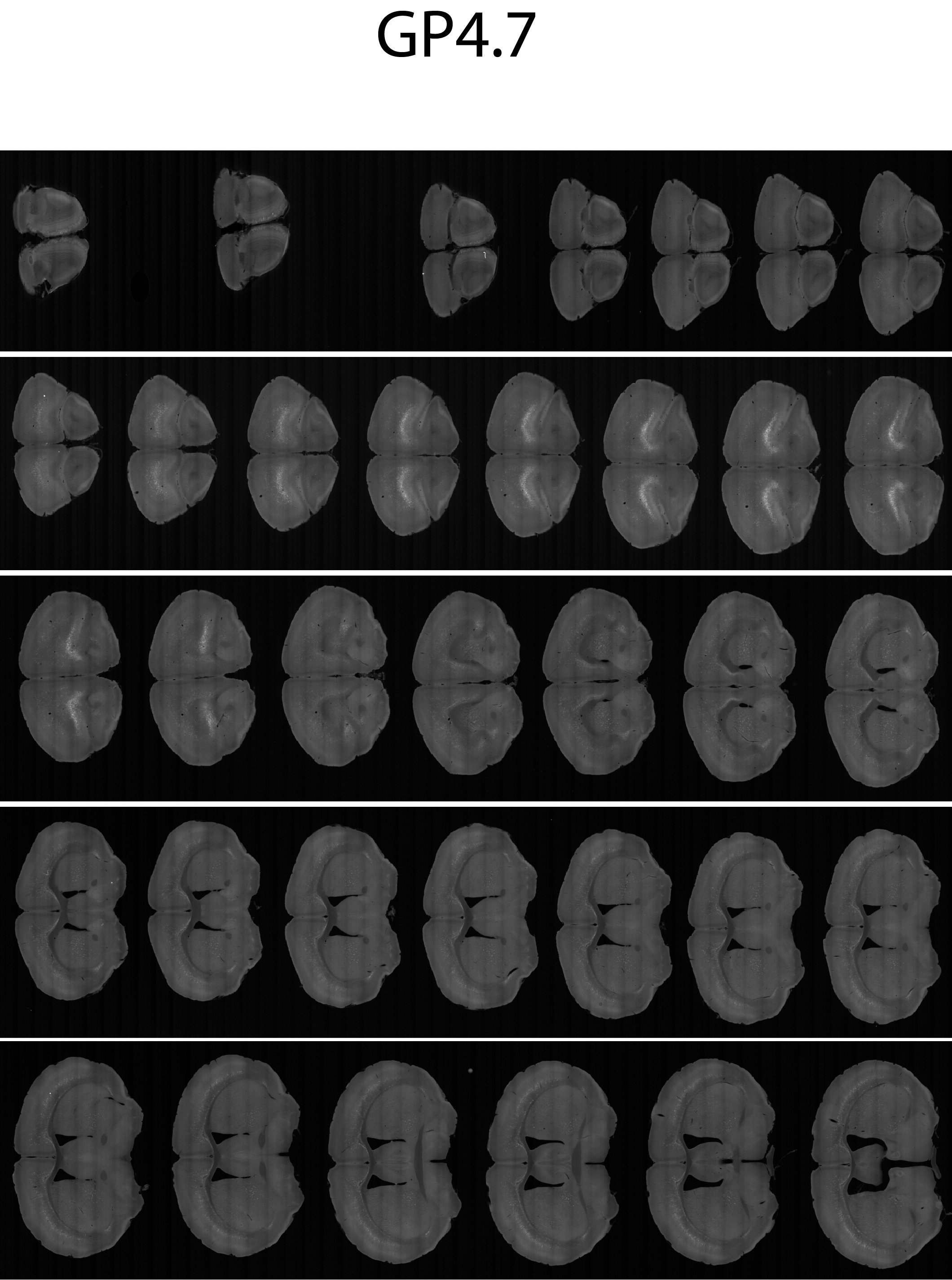

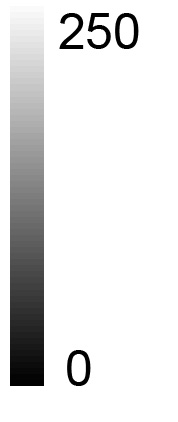


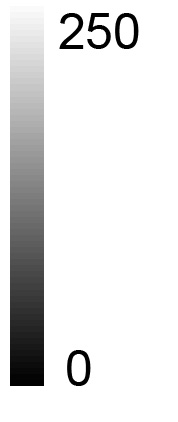

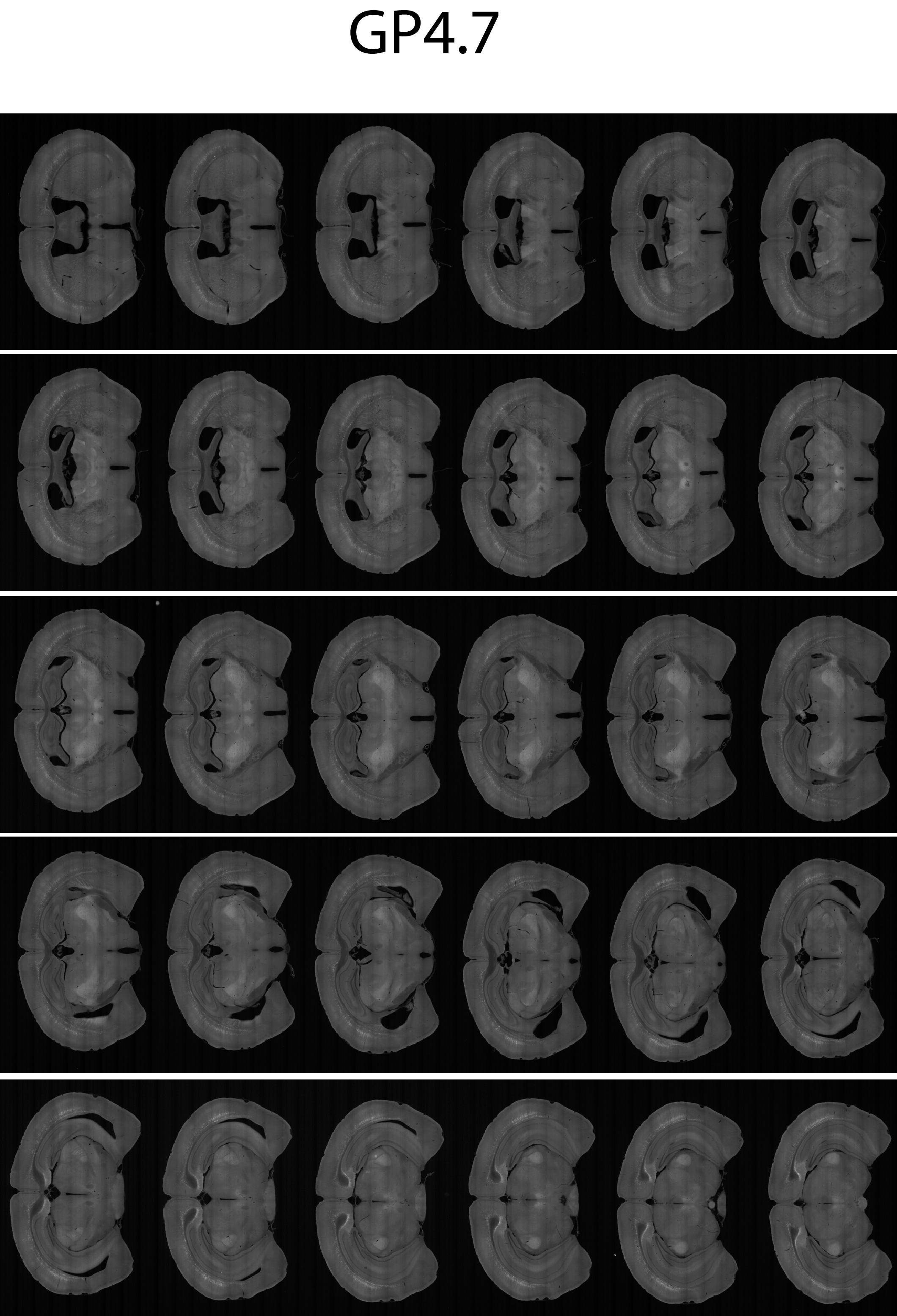


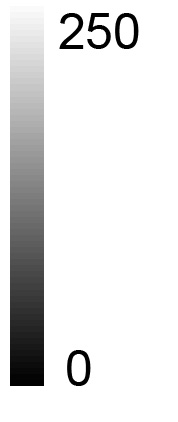

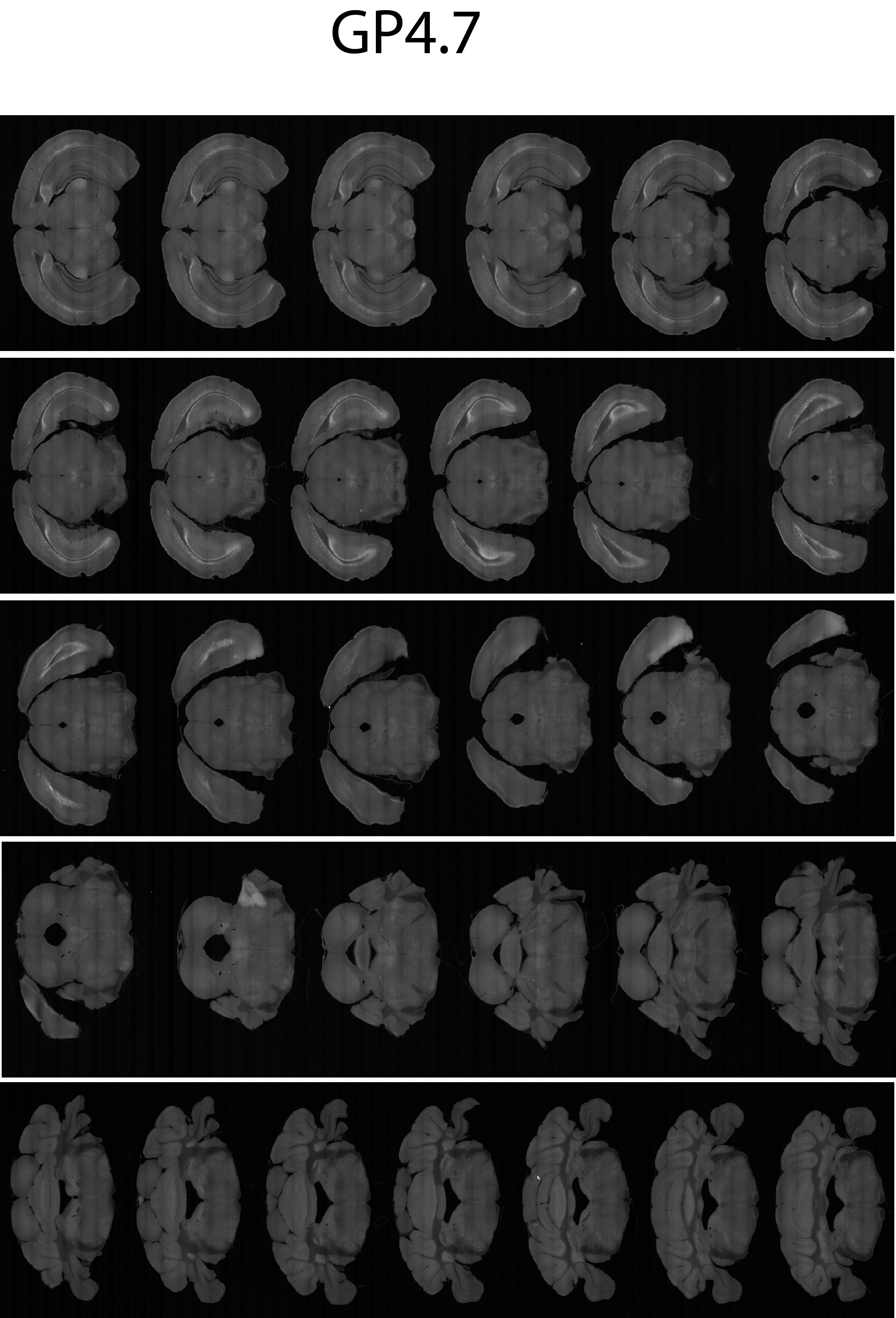


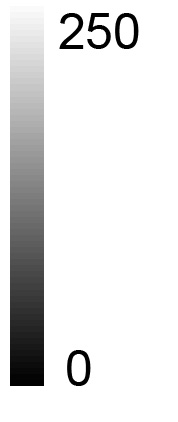

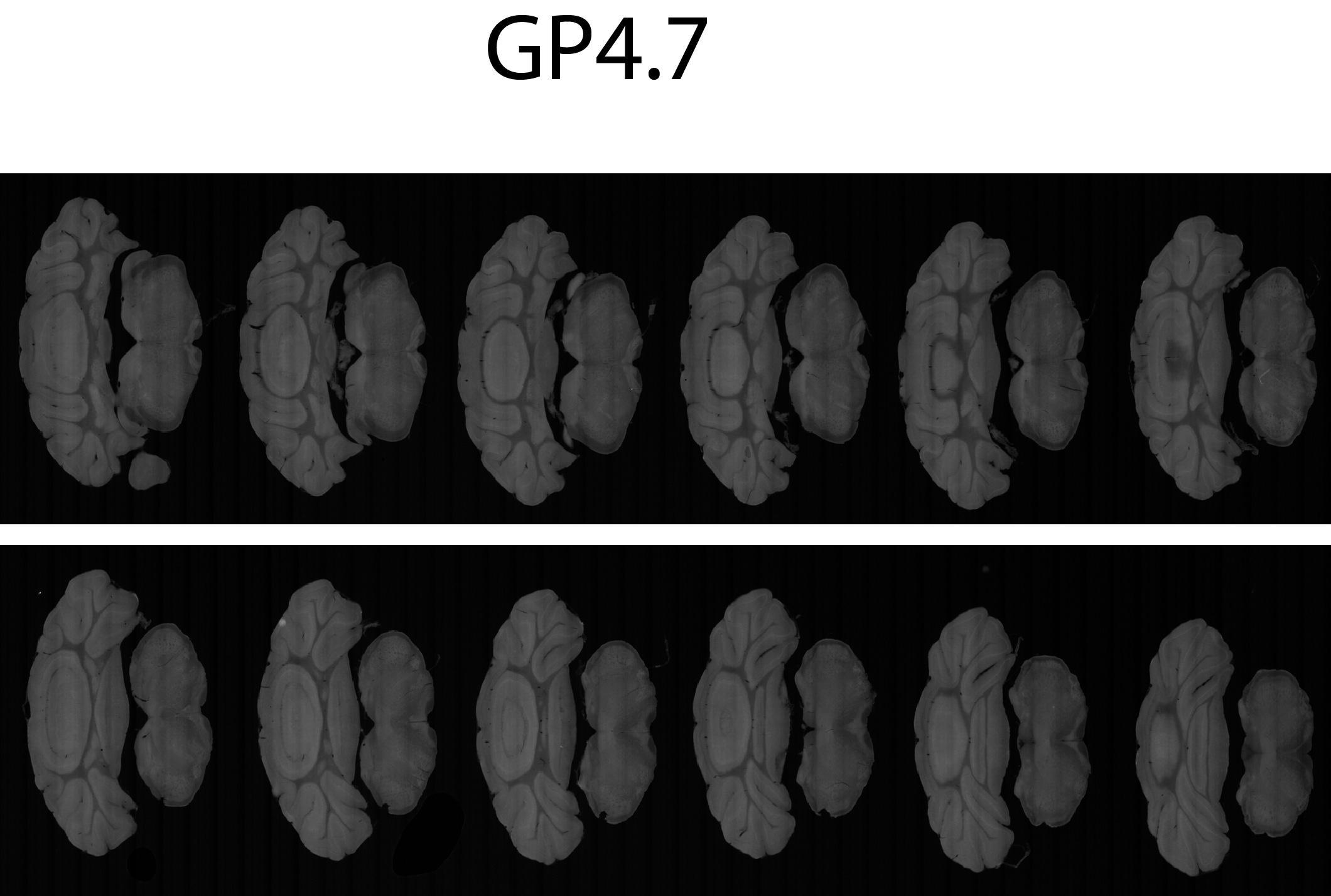


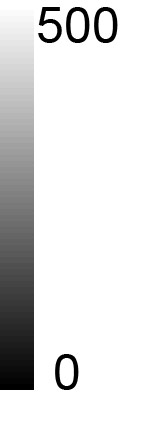

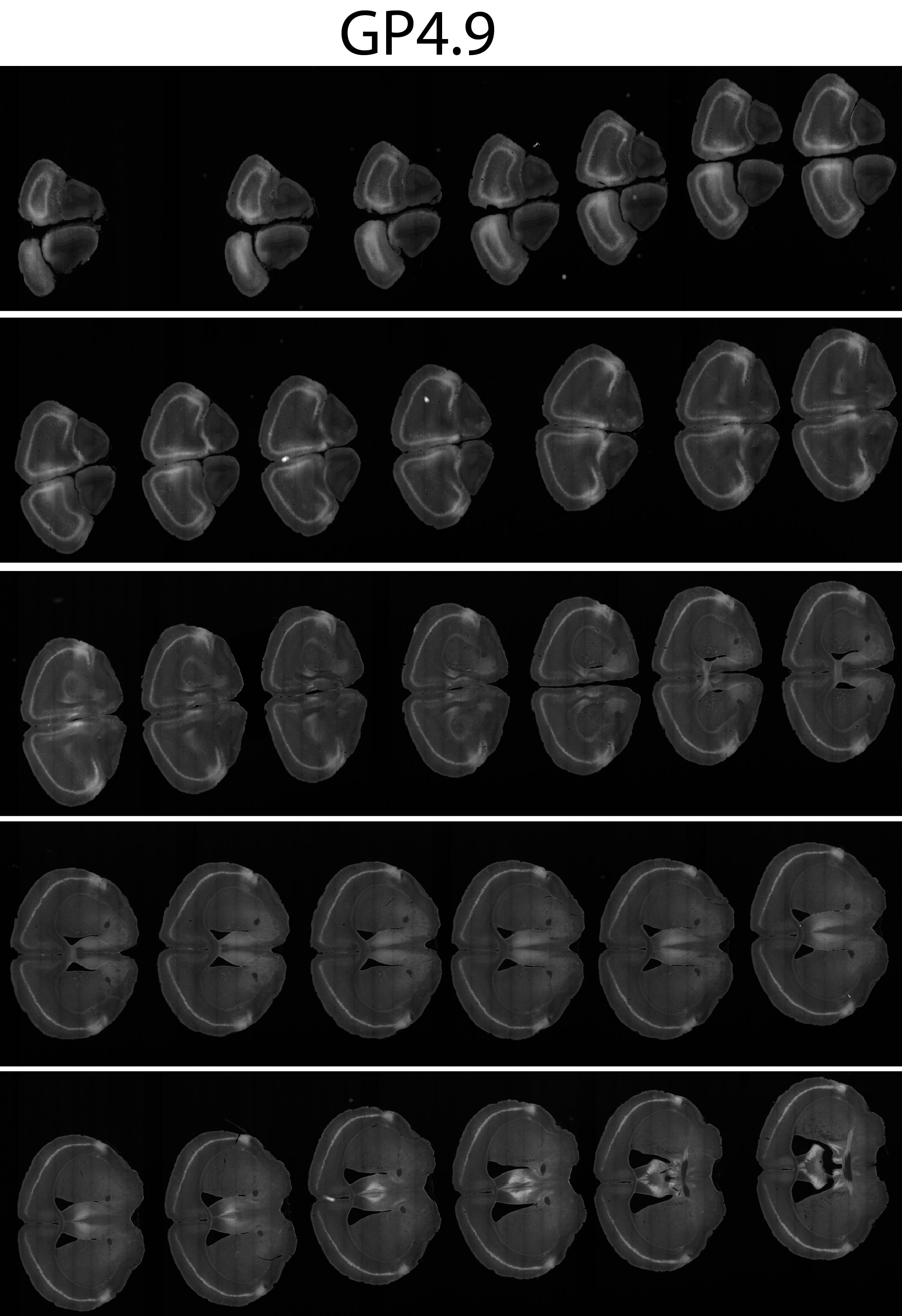


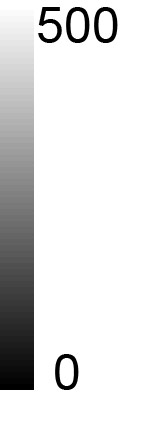

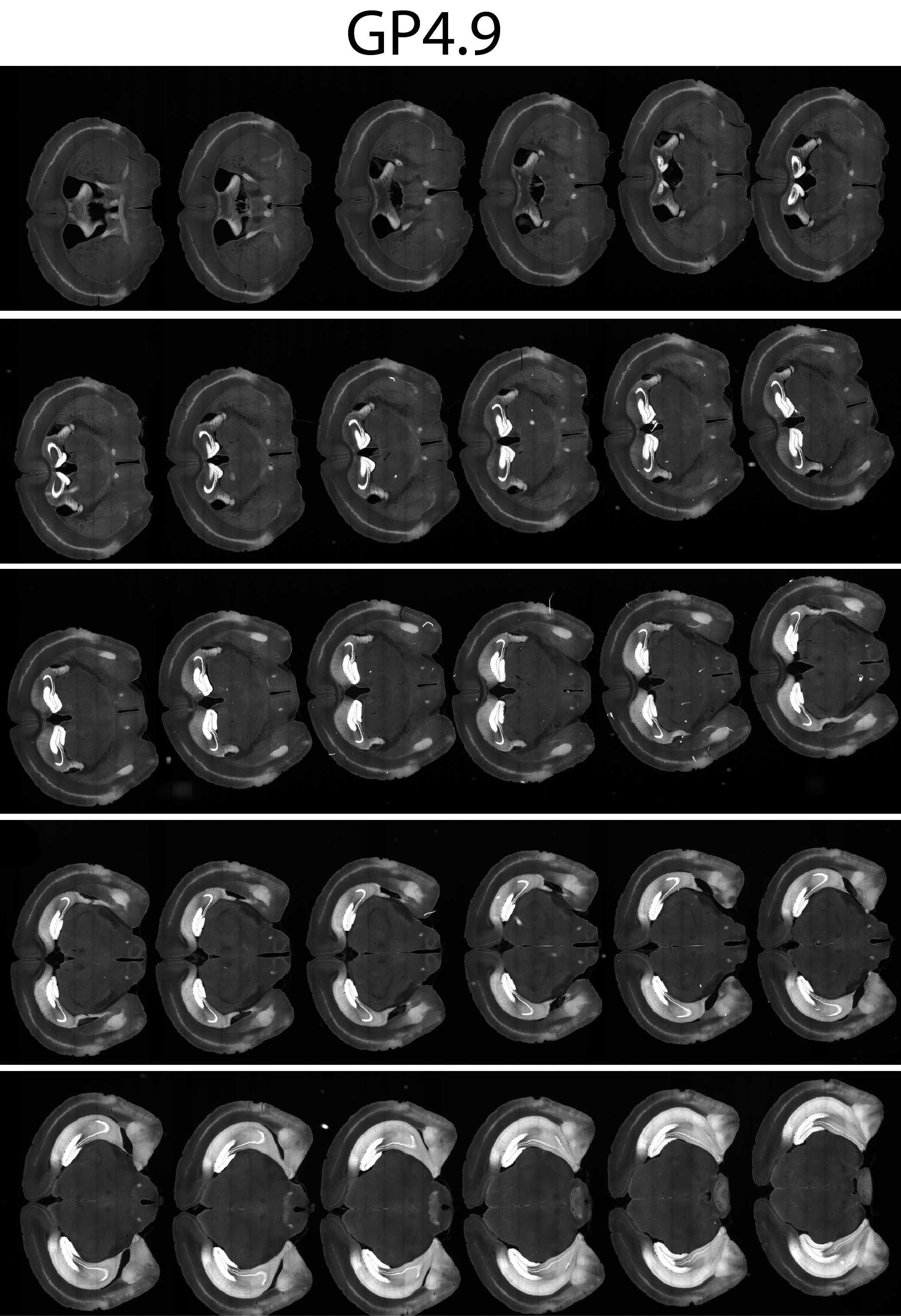


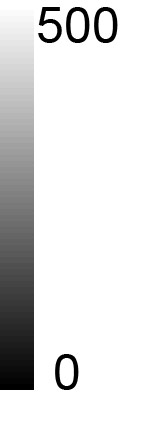

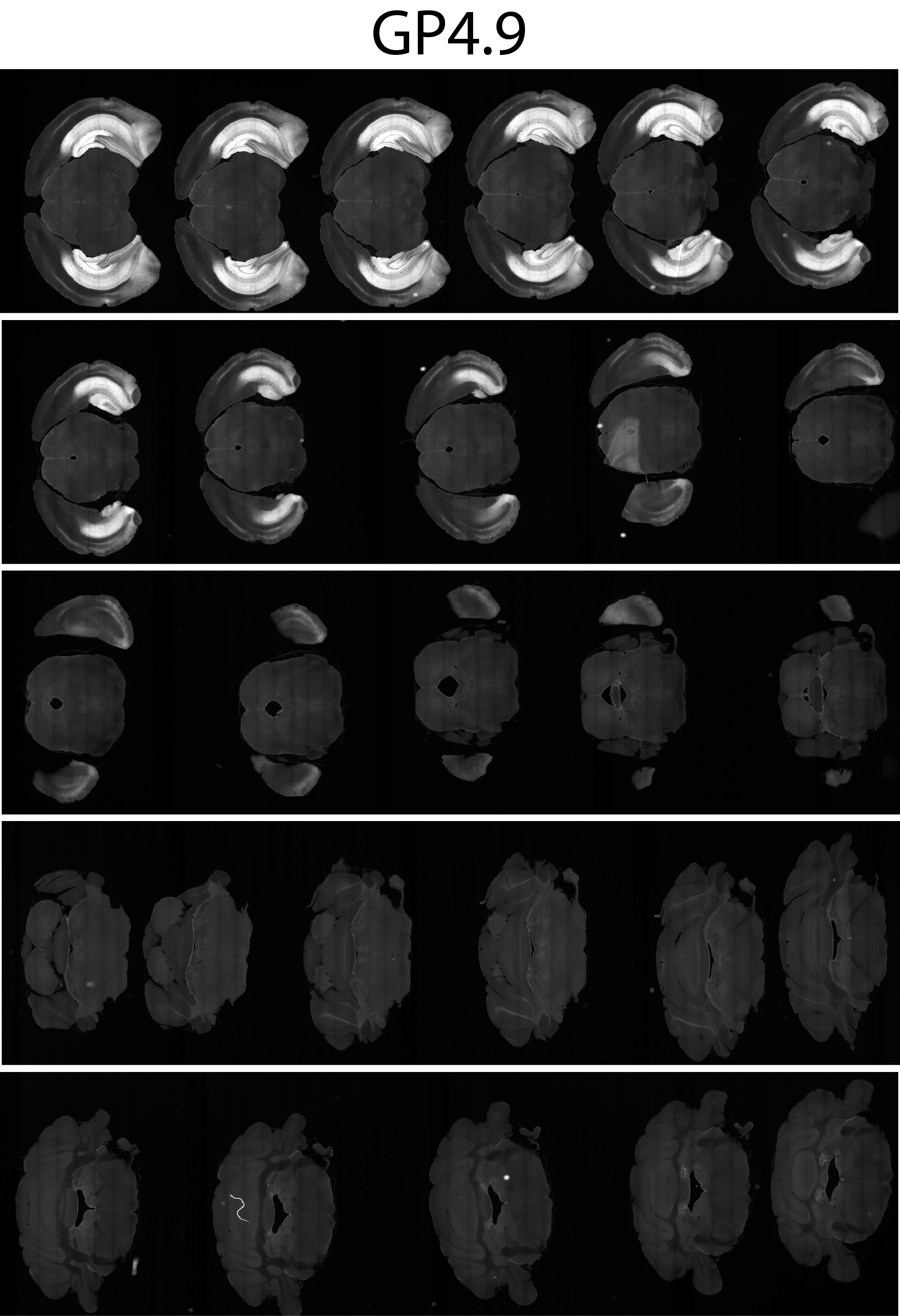


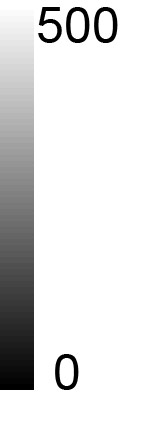

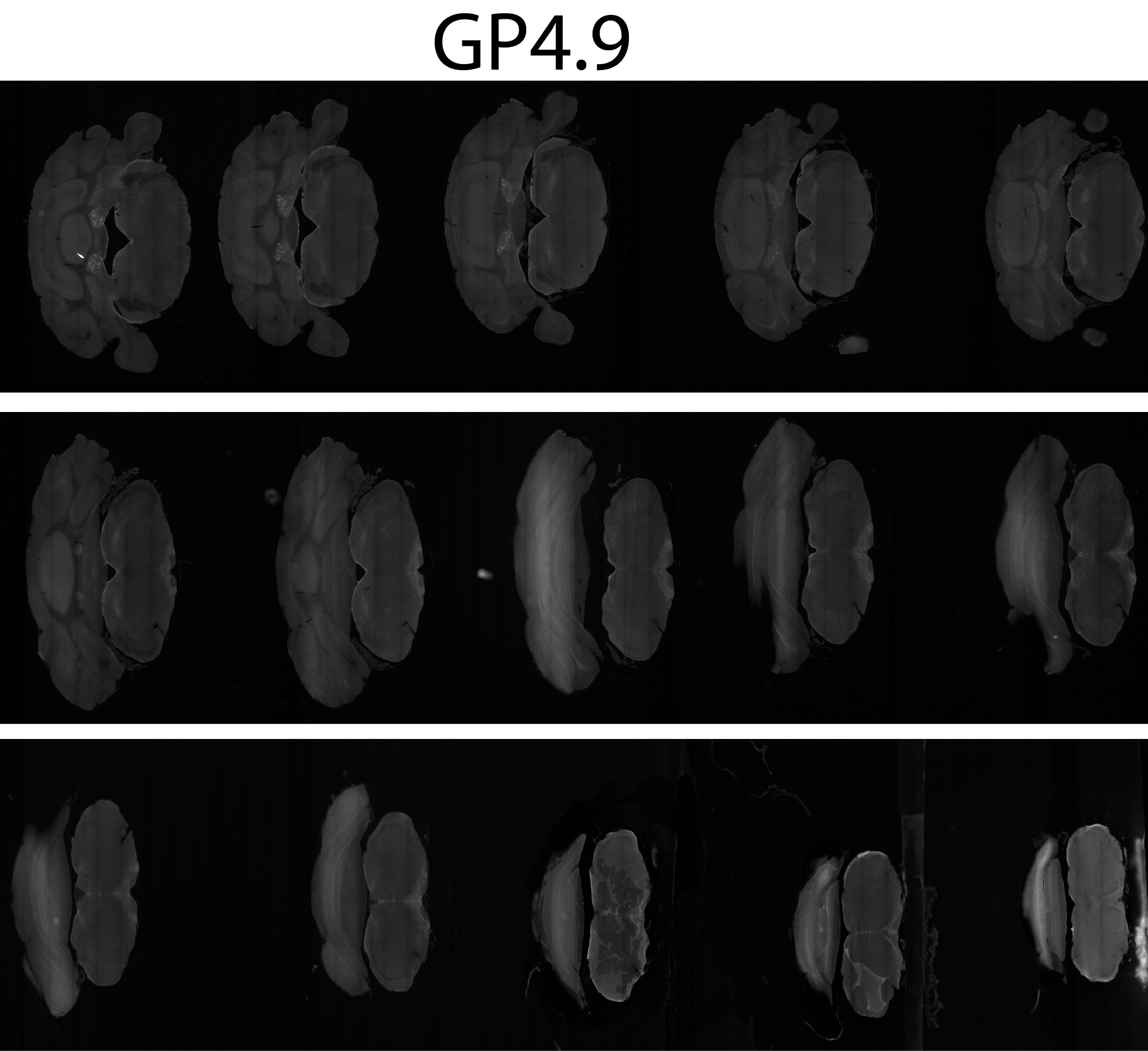
.


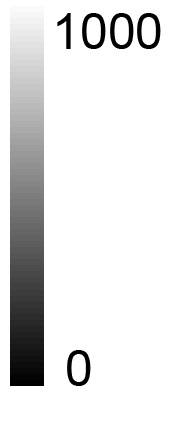

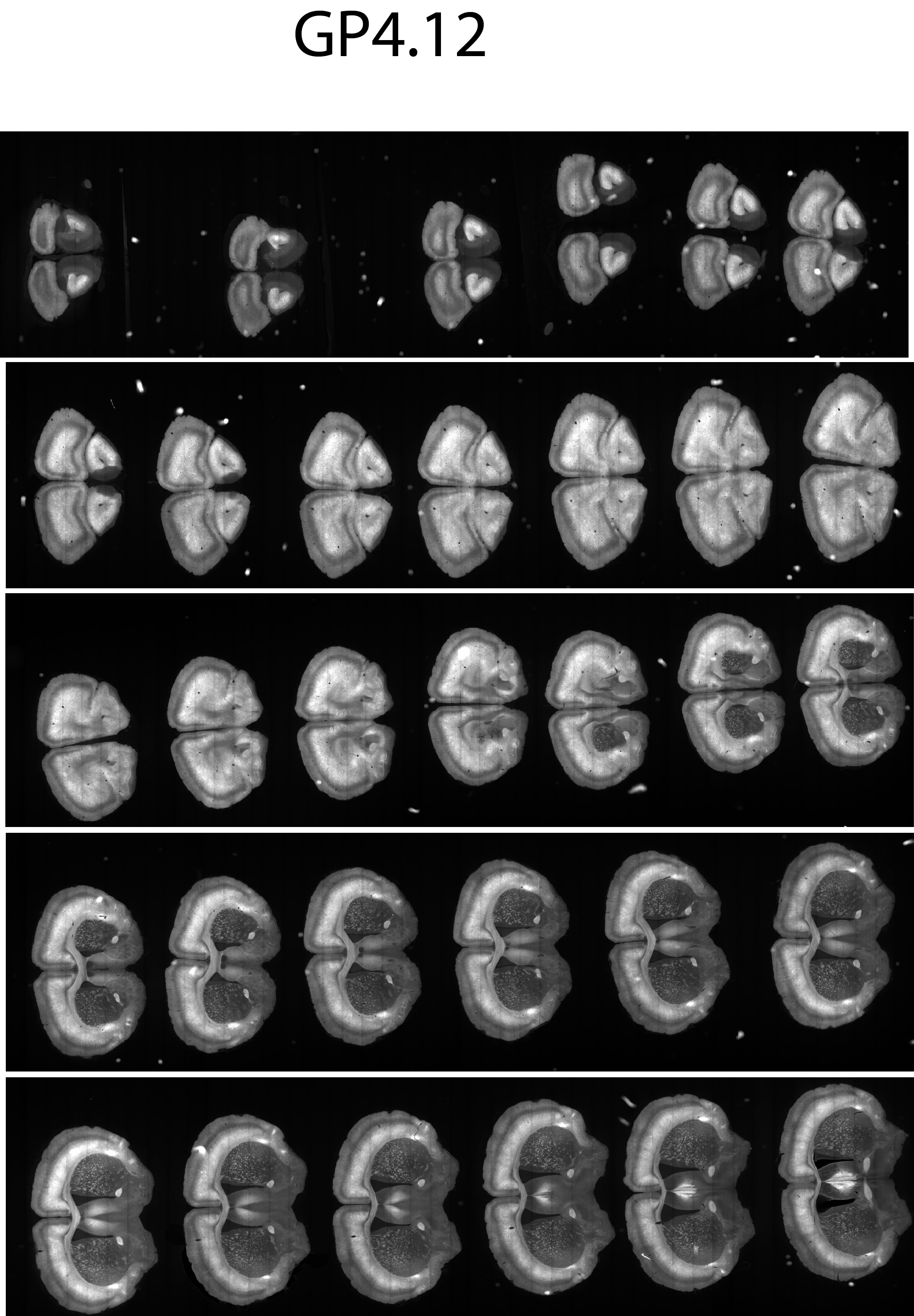


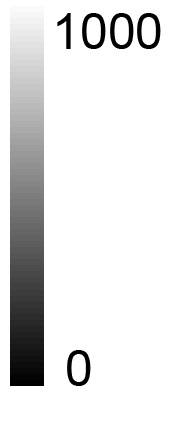

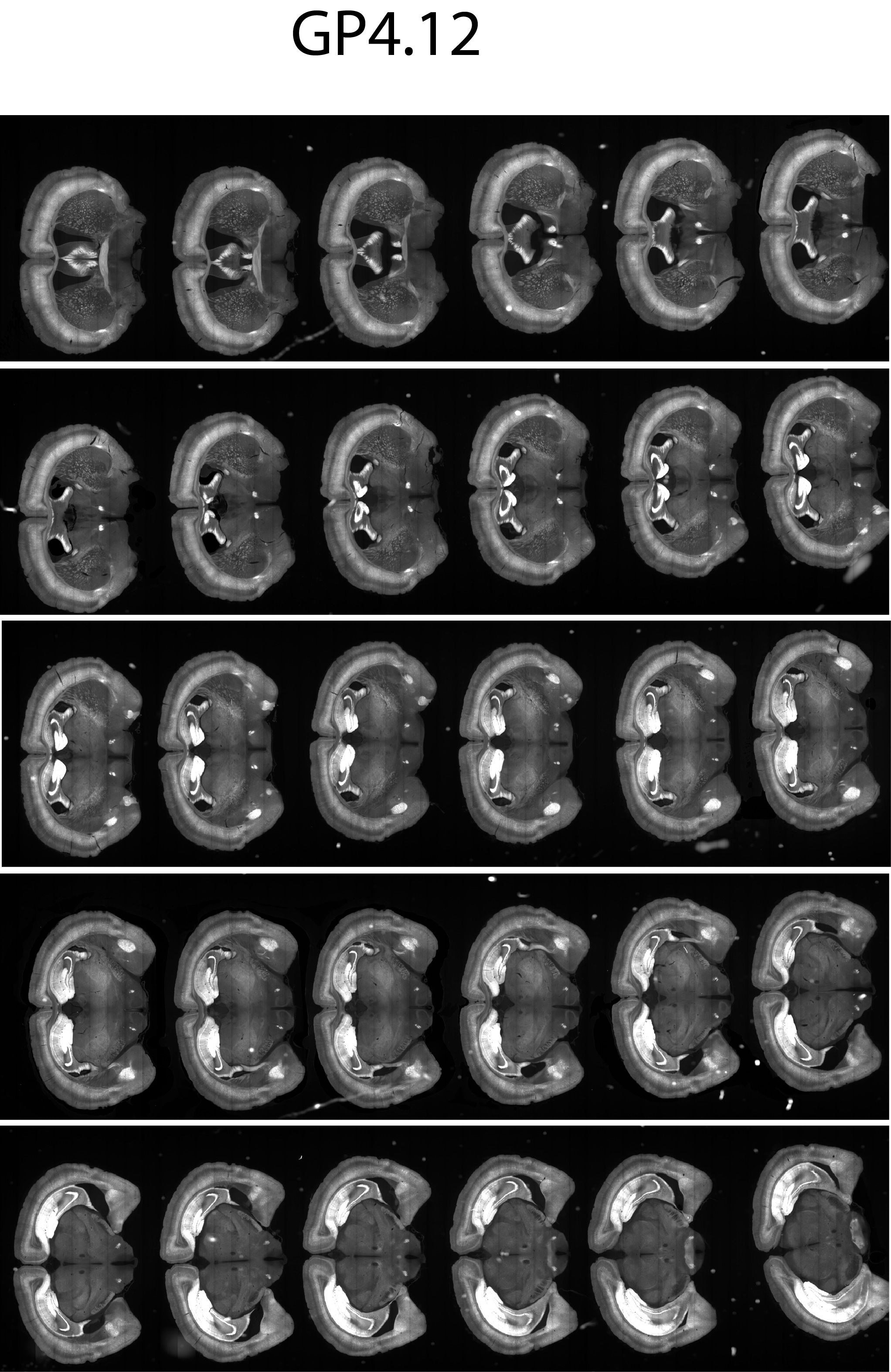


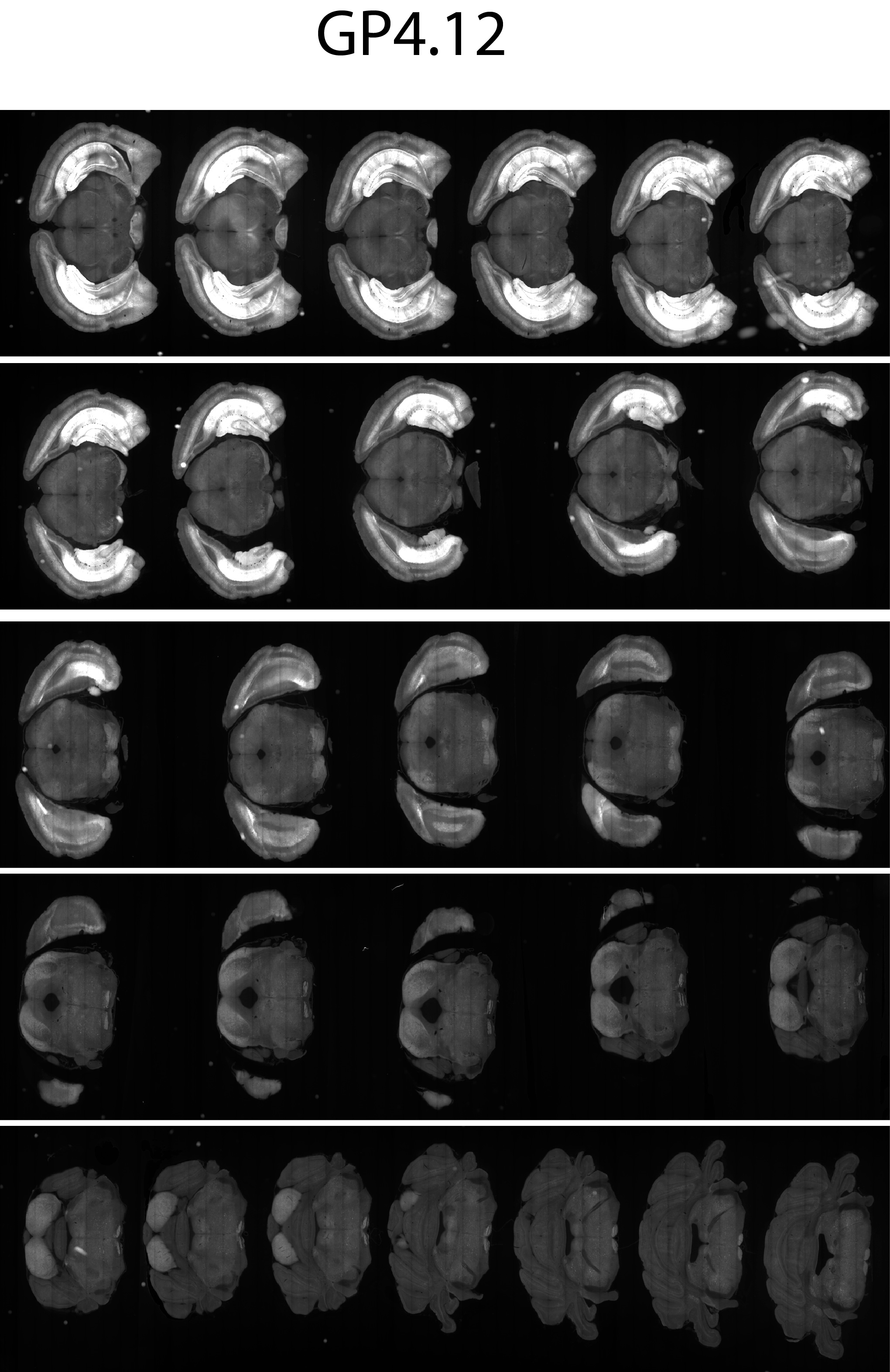

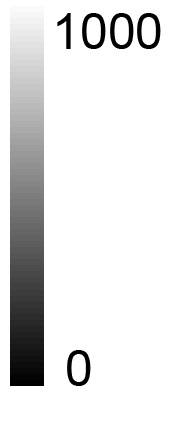


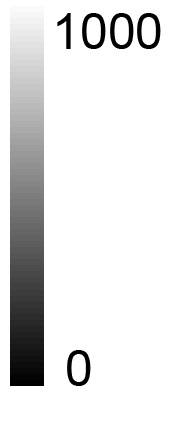
.
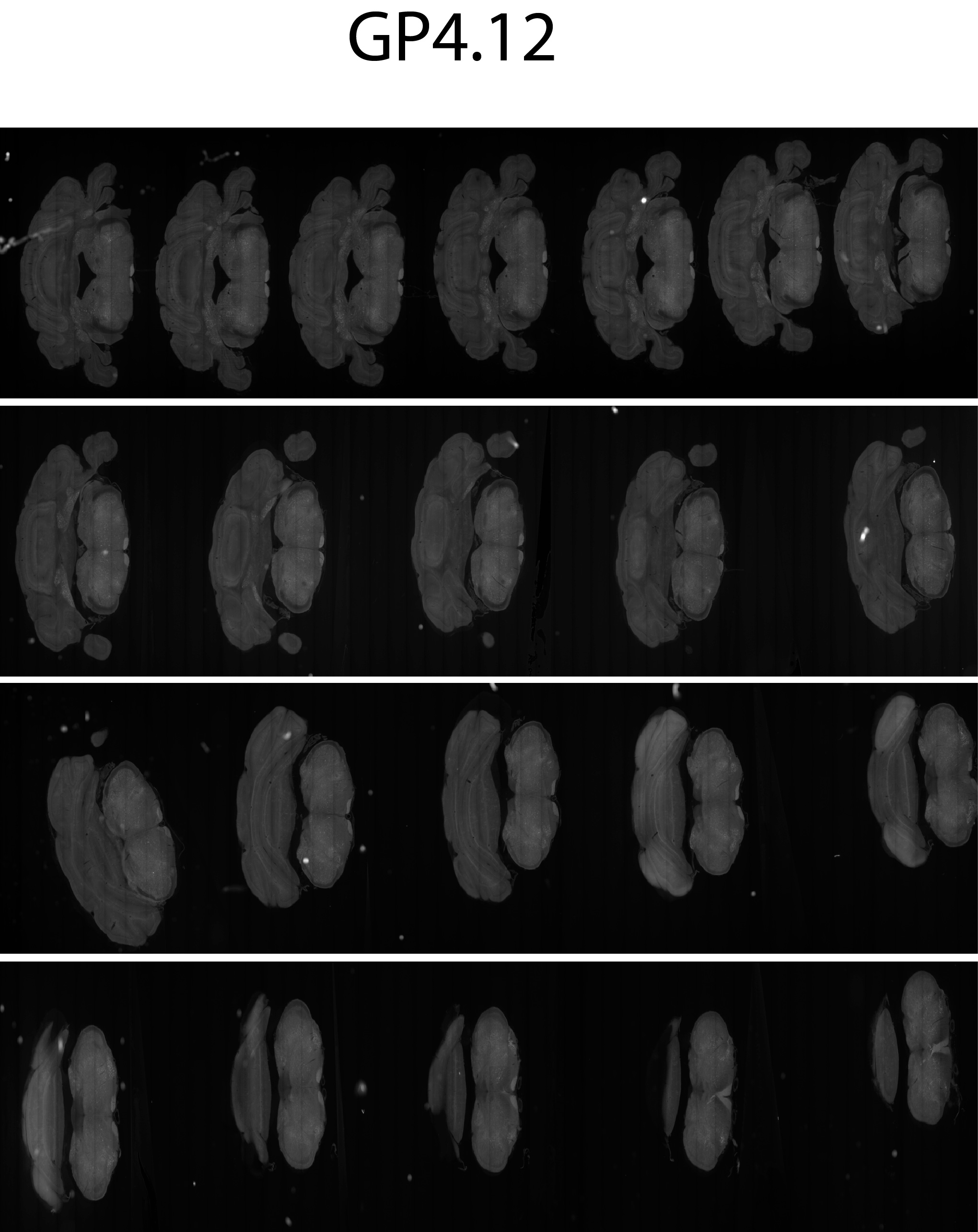


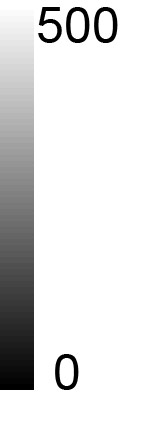

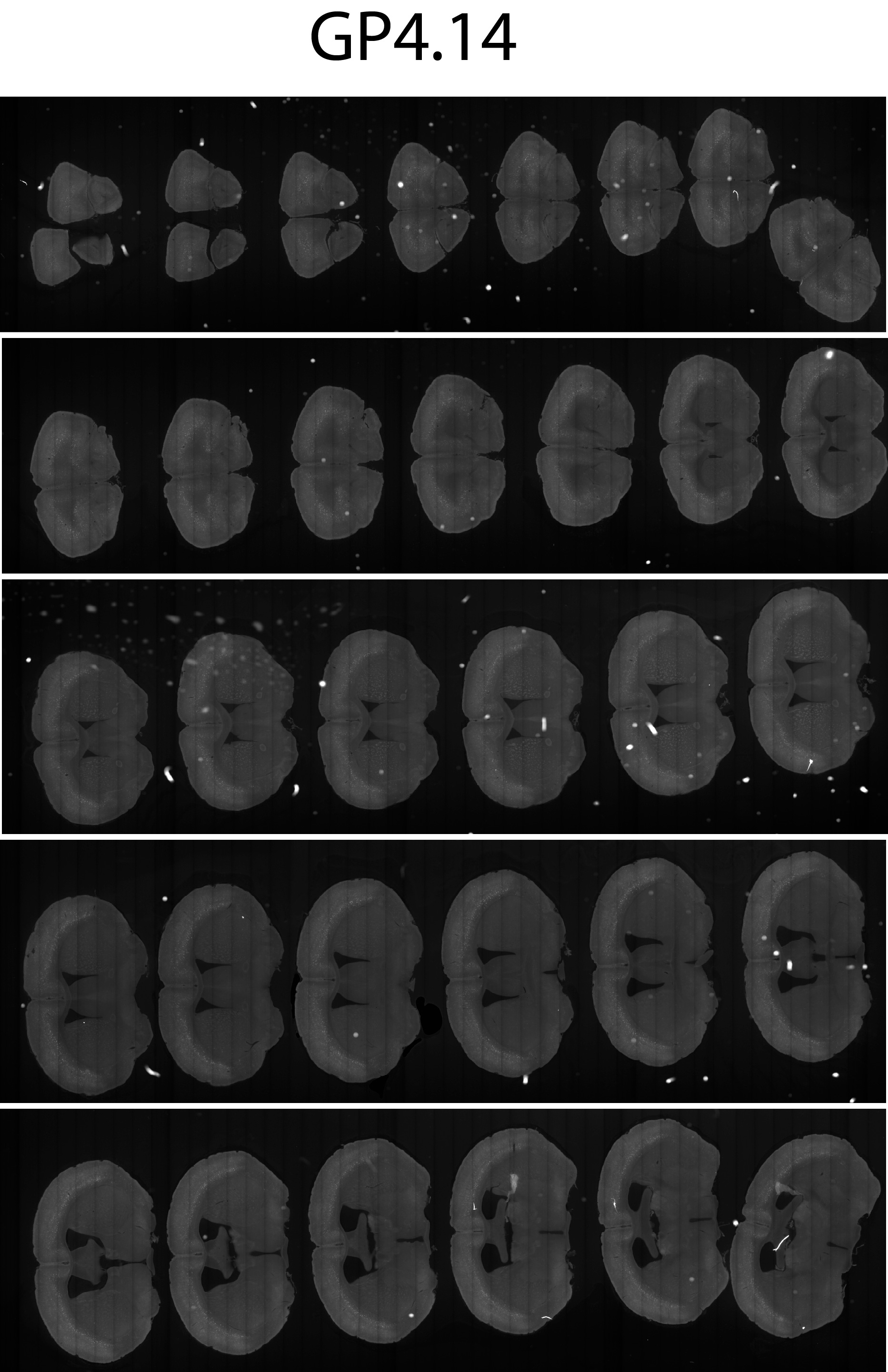


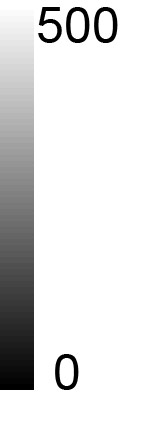

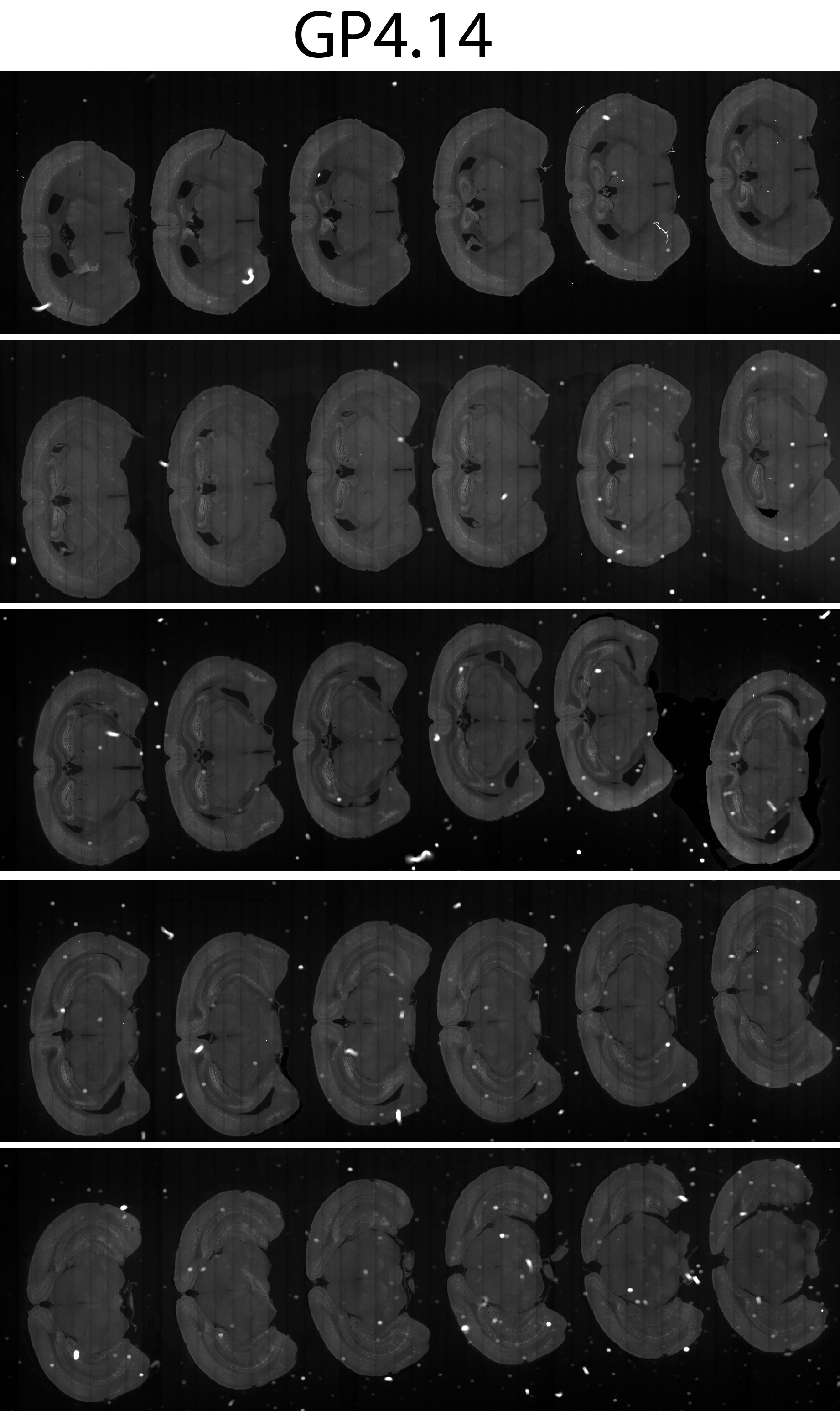


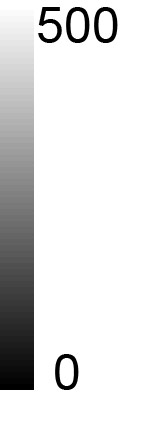


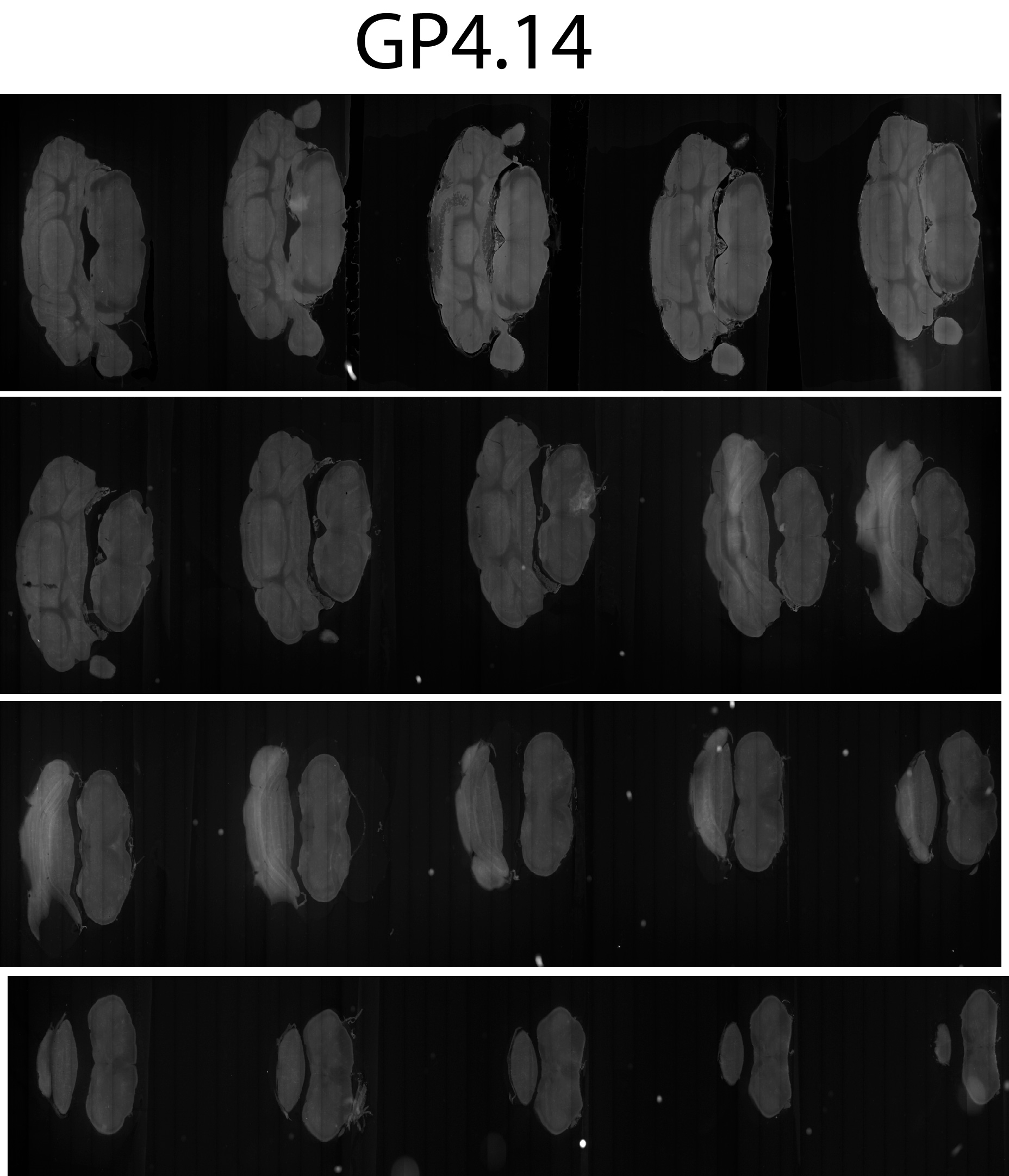


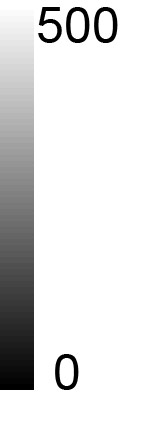

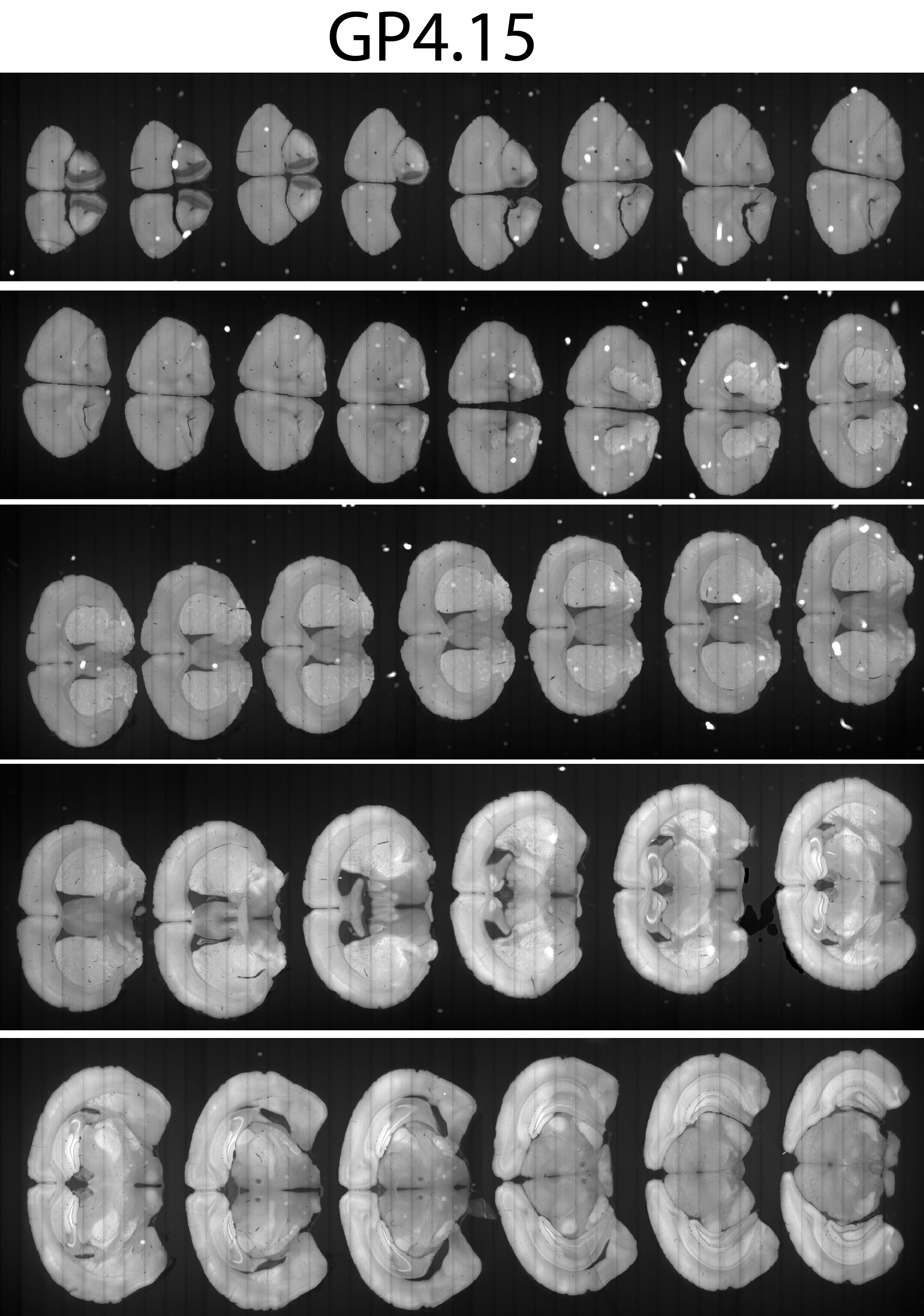


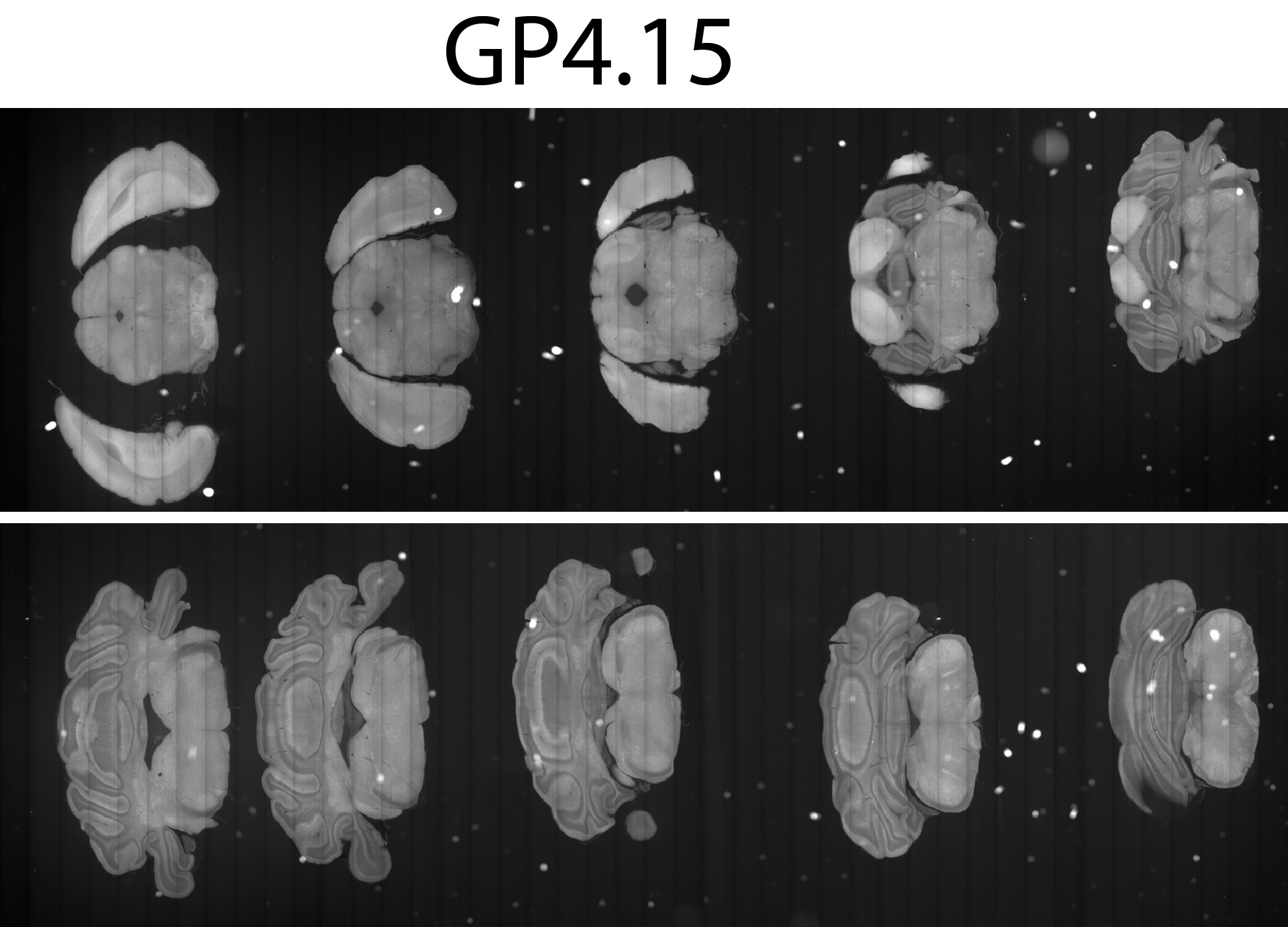

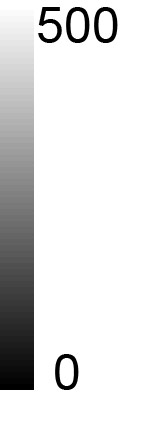


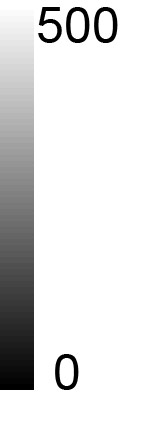

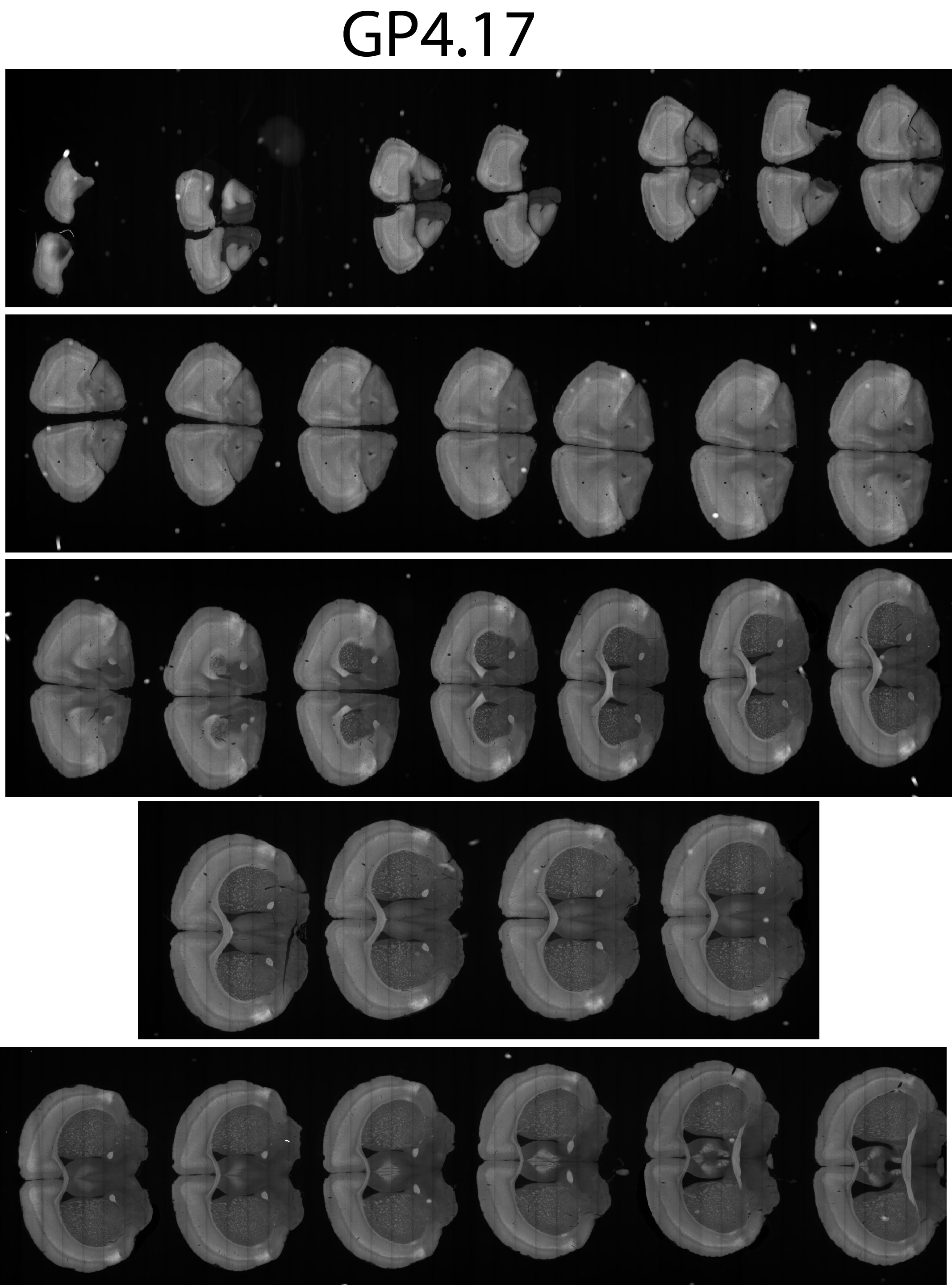


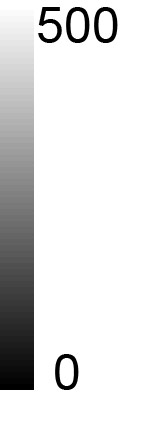

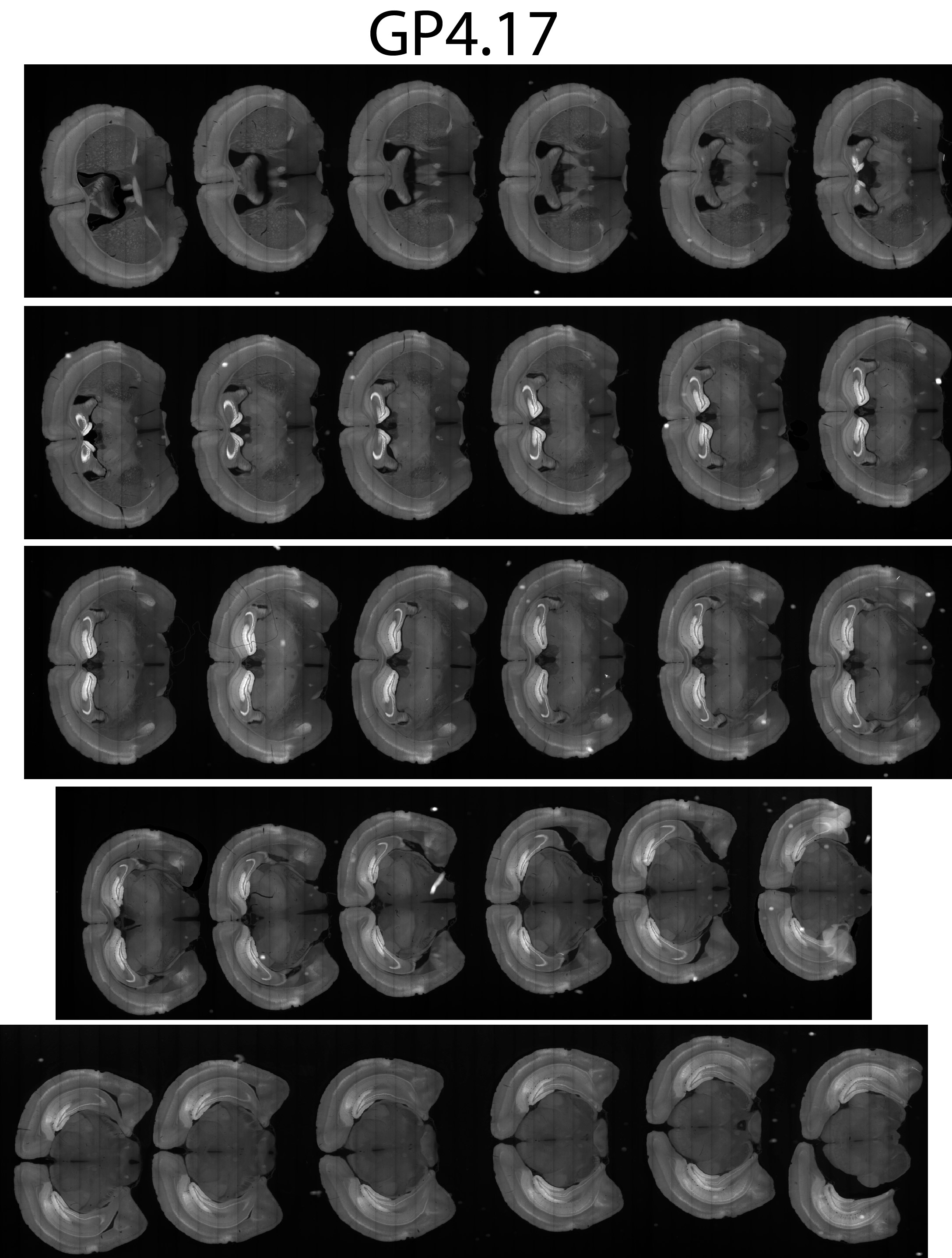


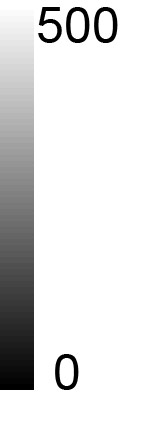

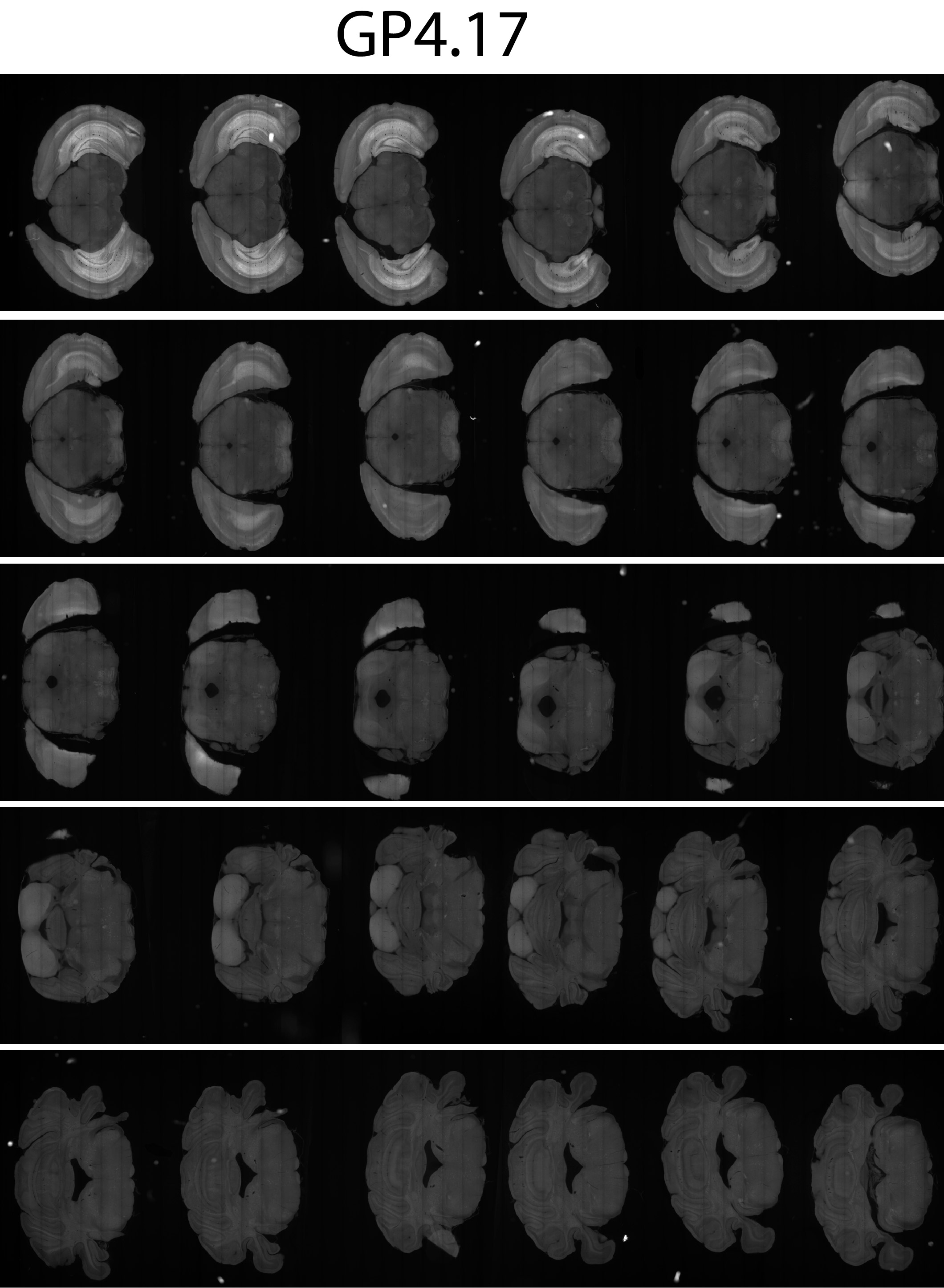


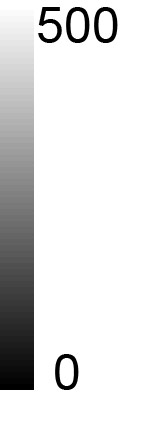

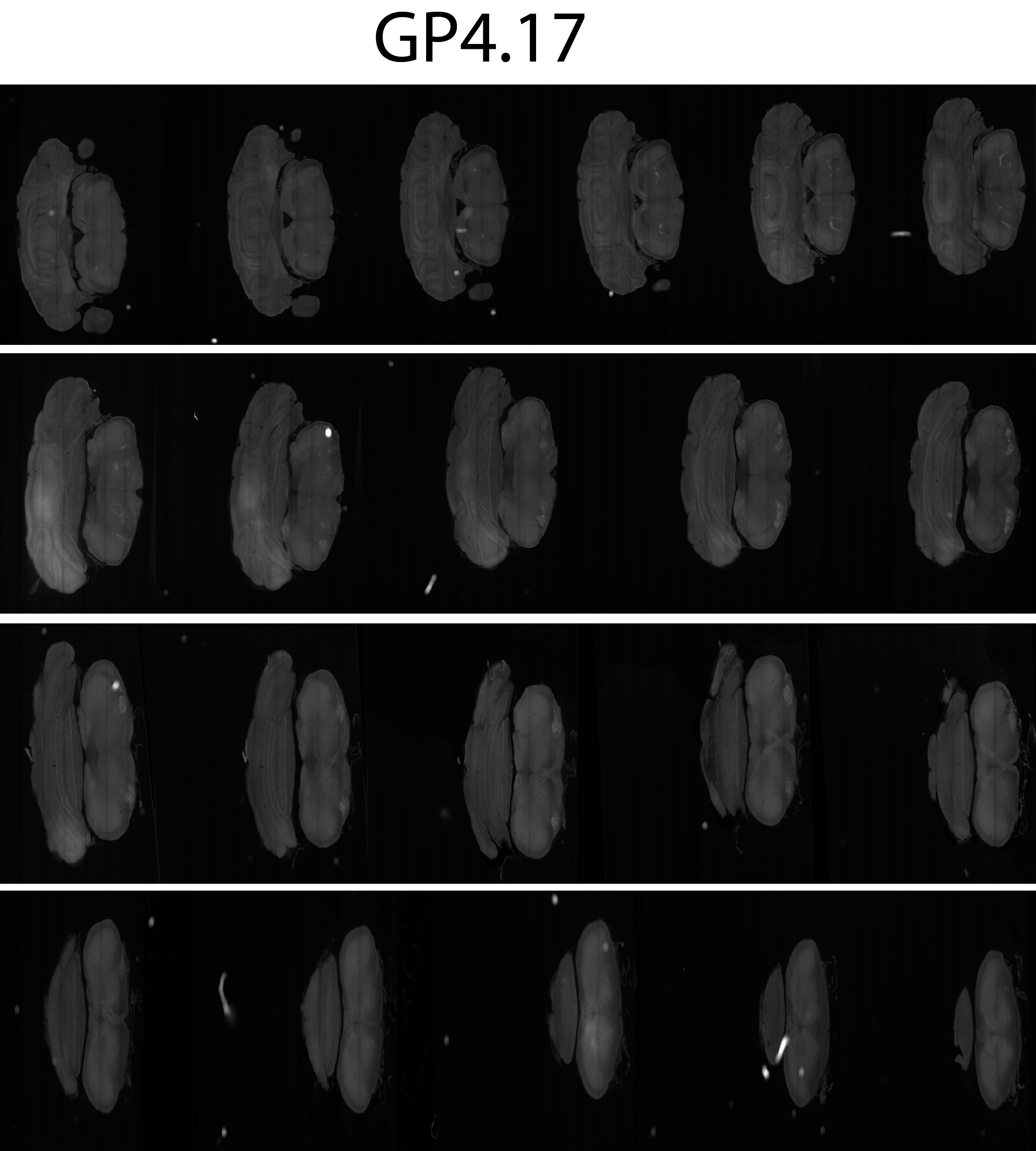


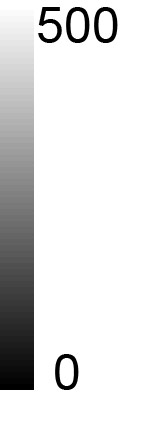

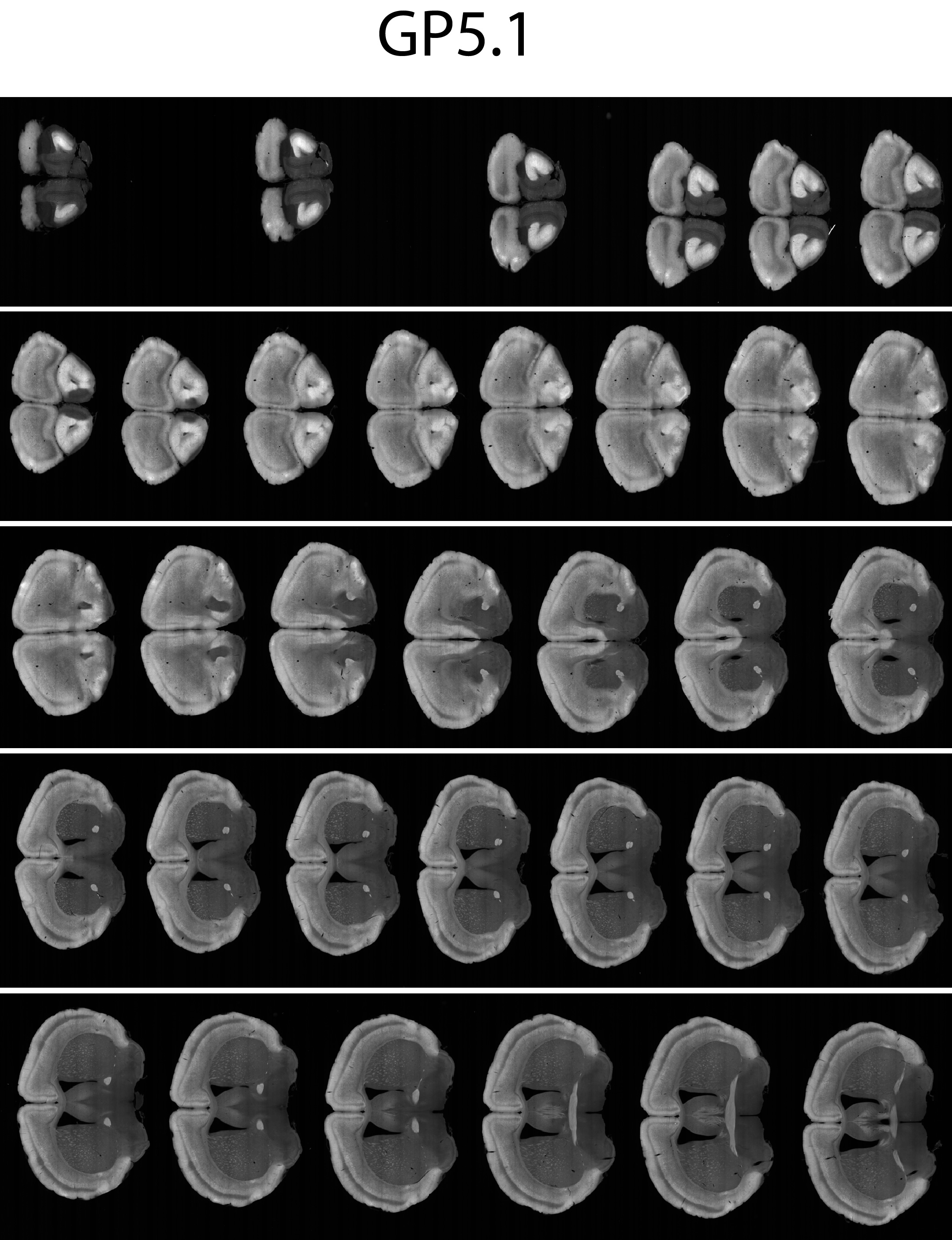


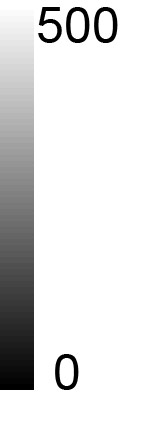

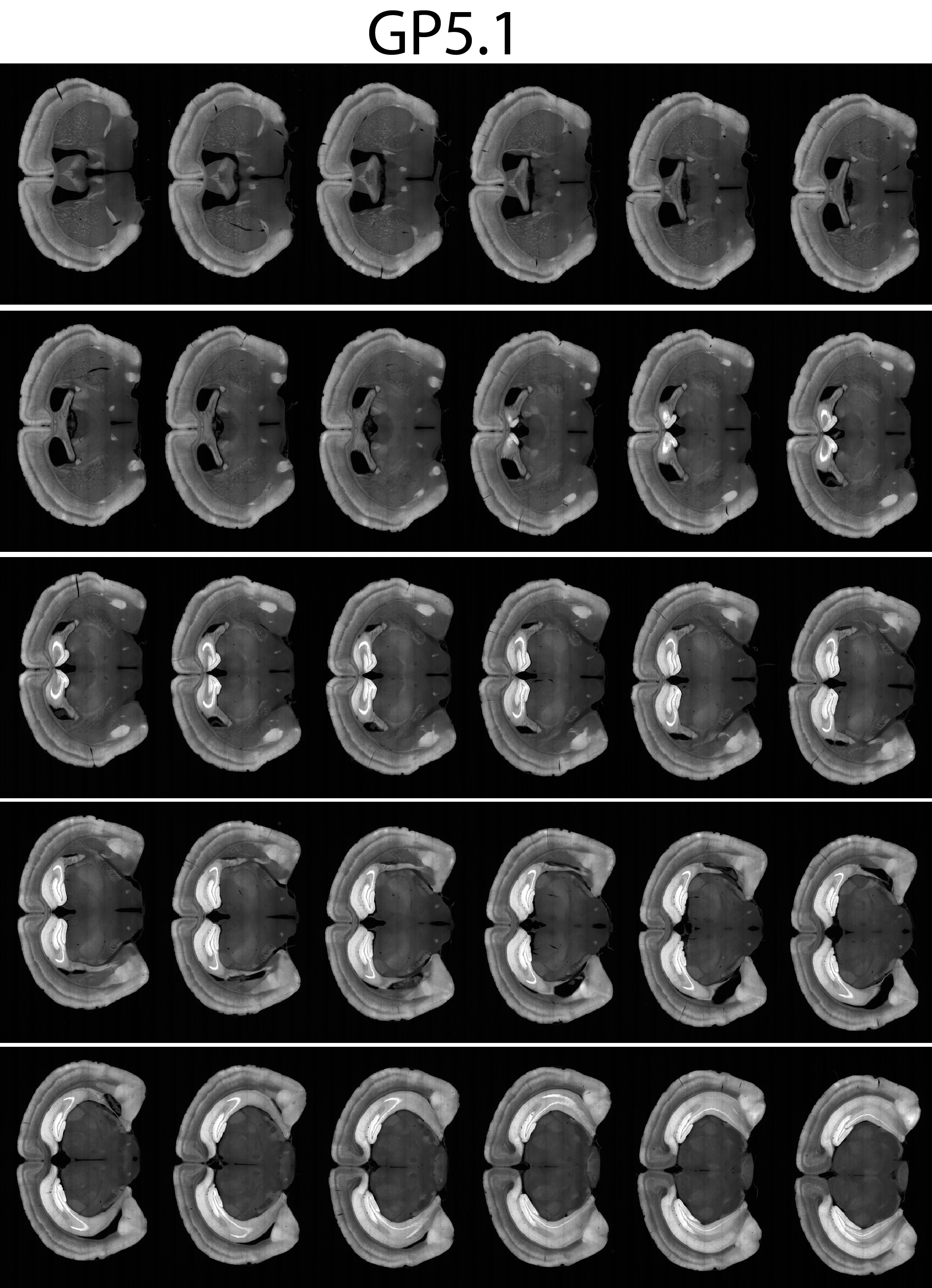


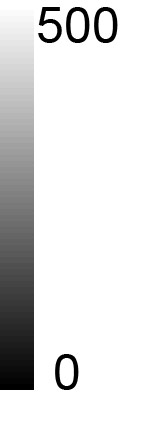

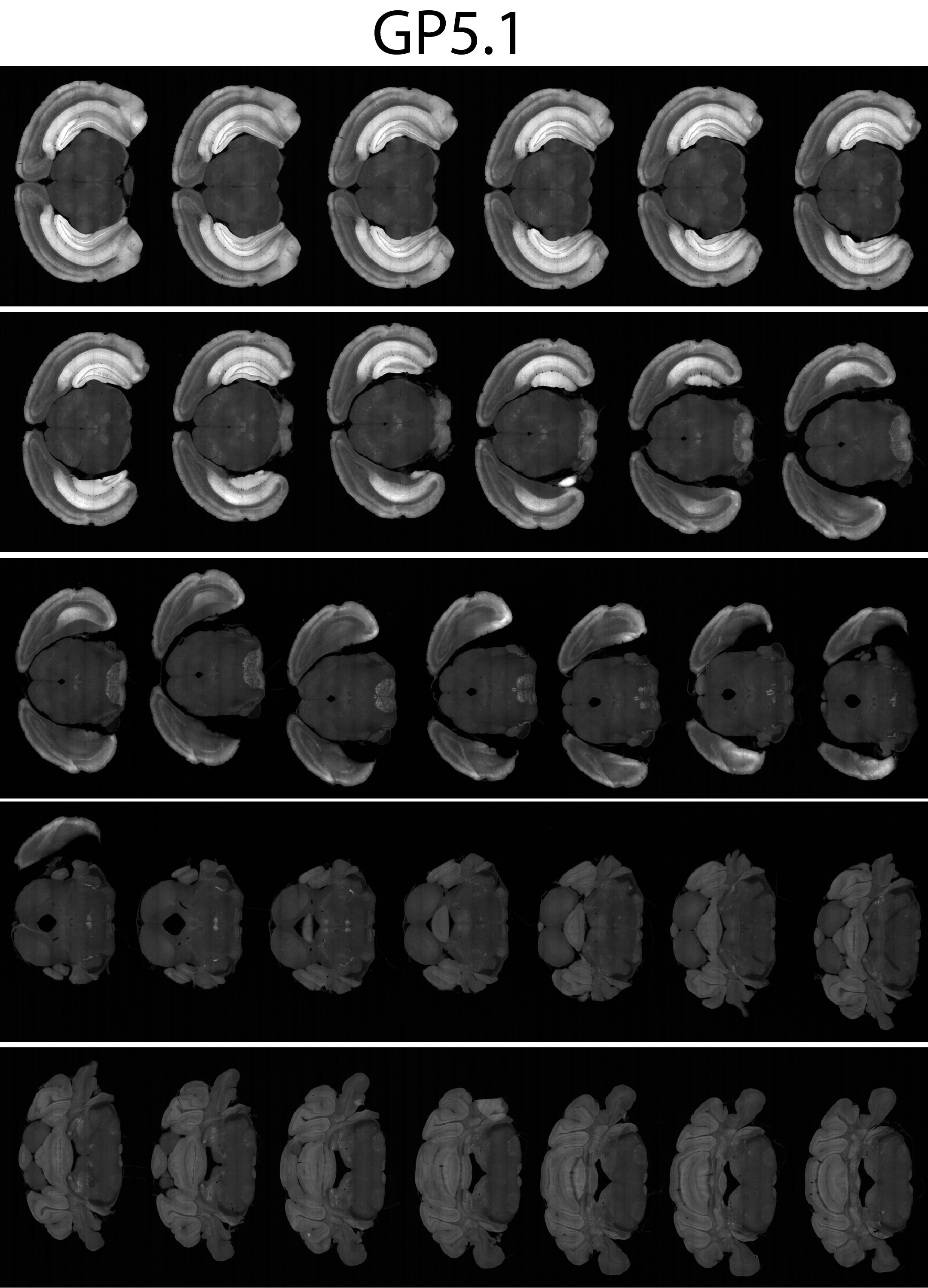


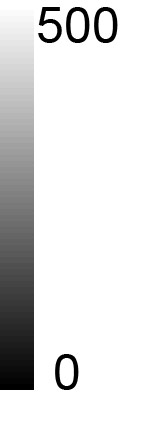

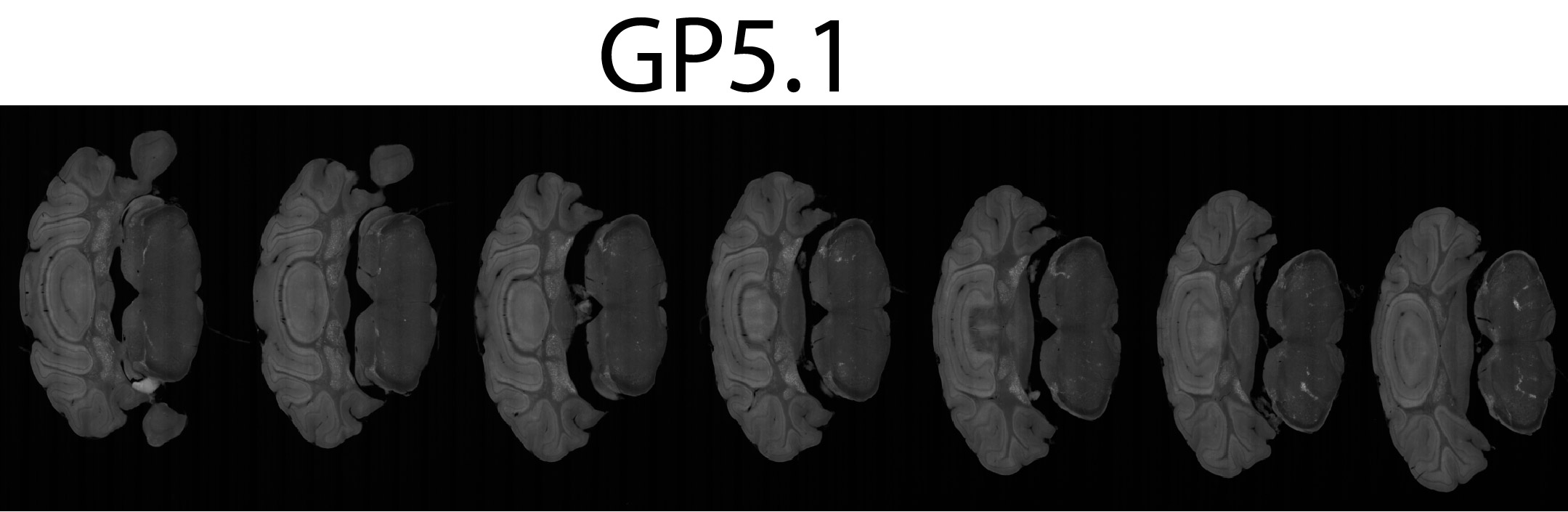


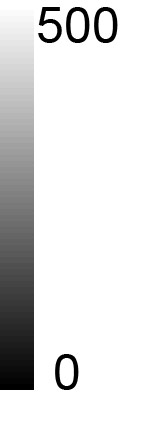

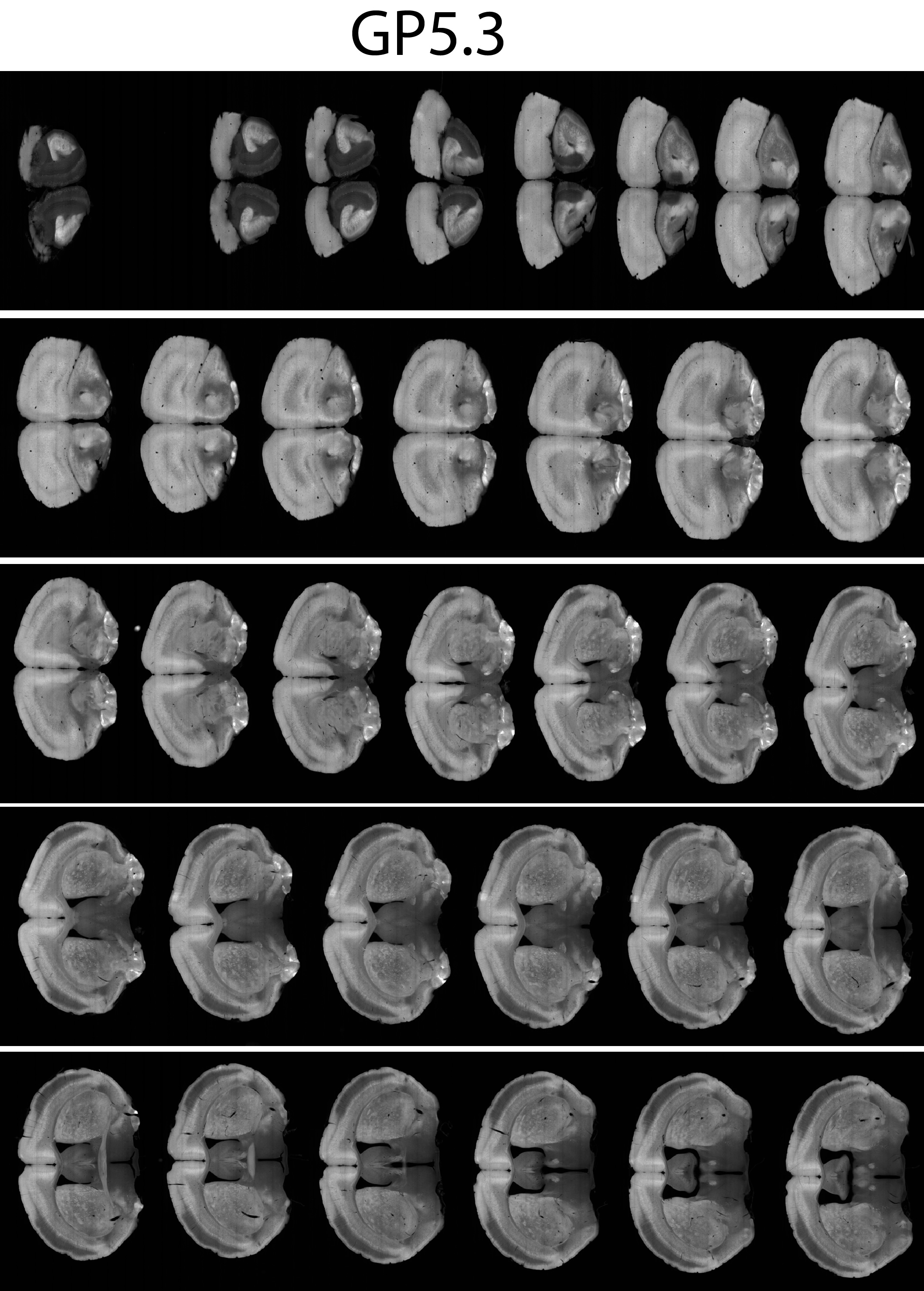


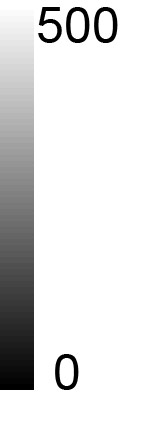

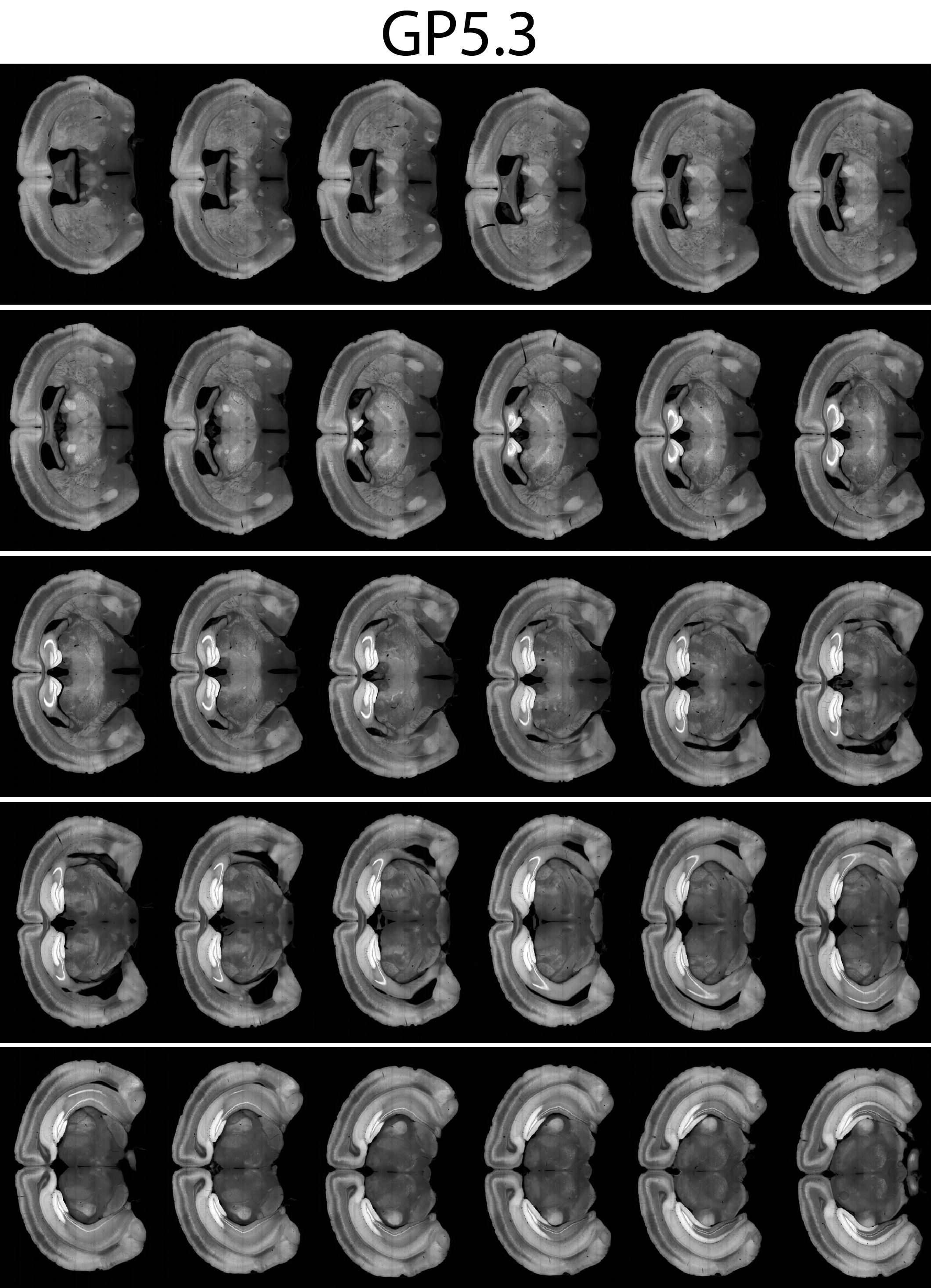


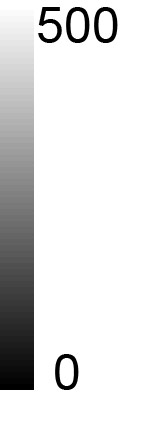

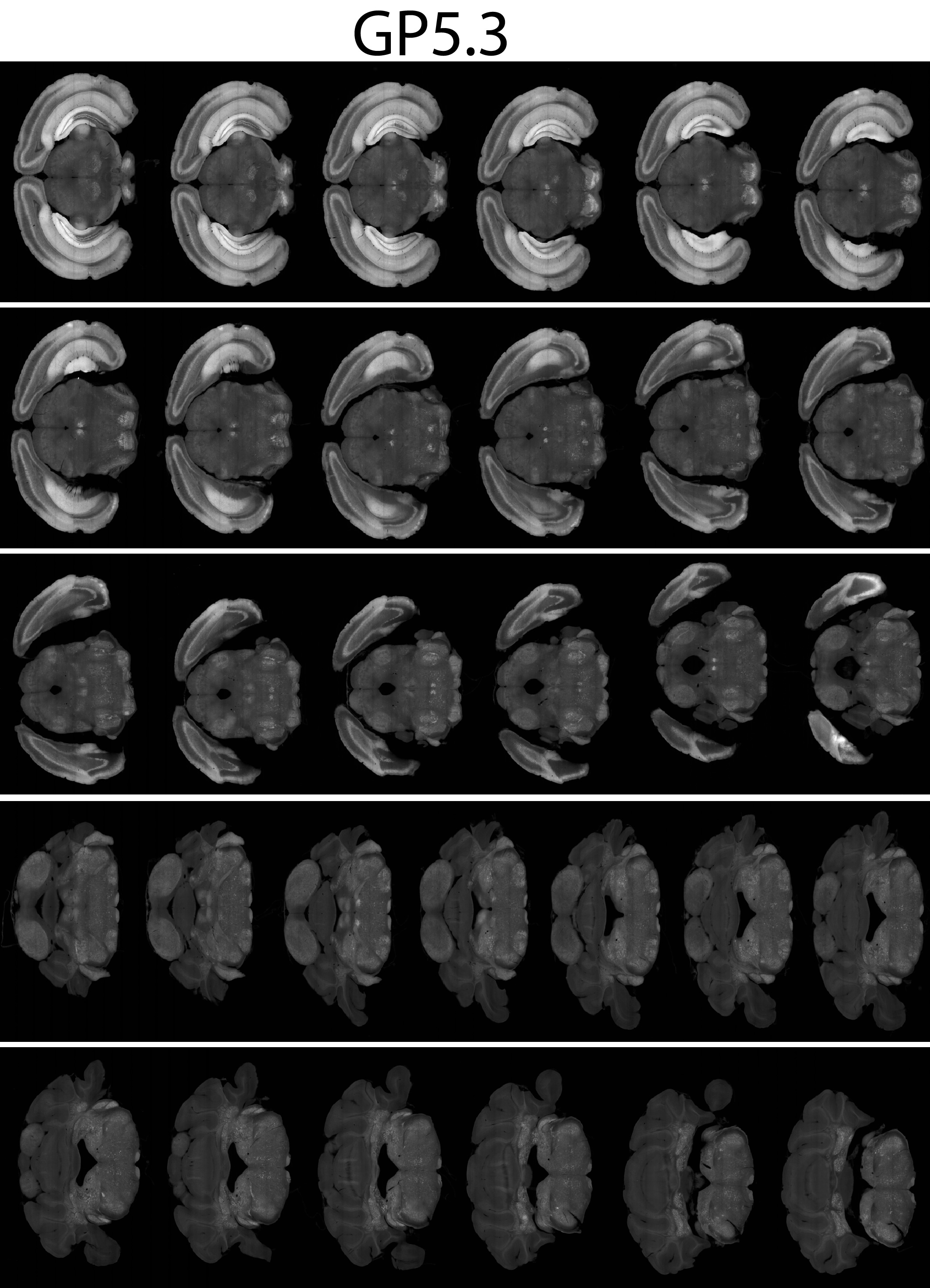


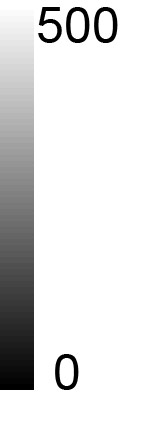

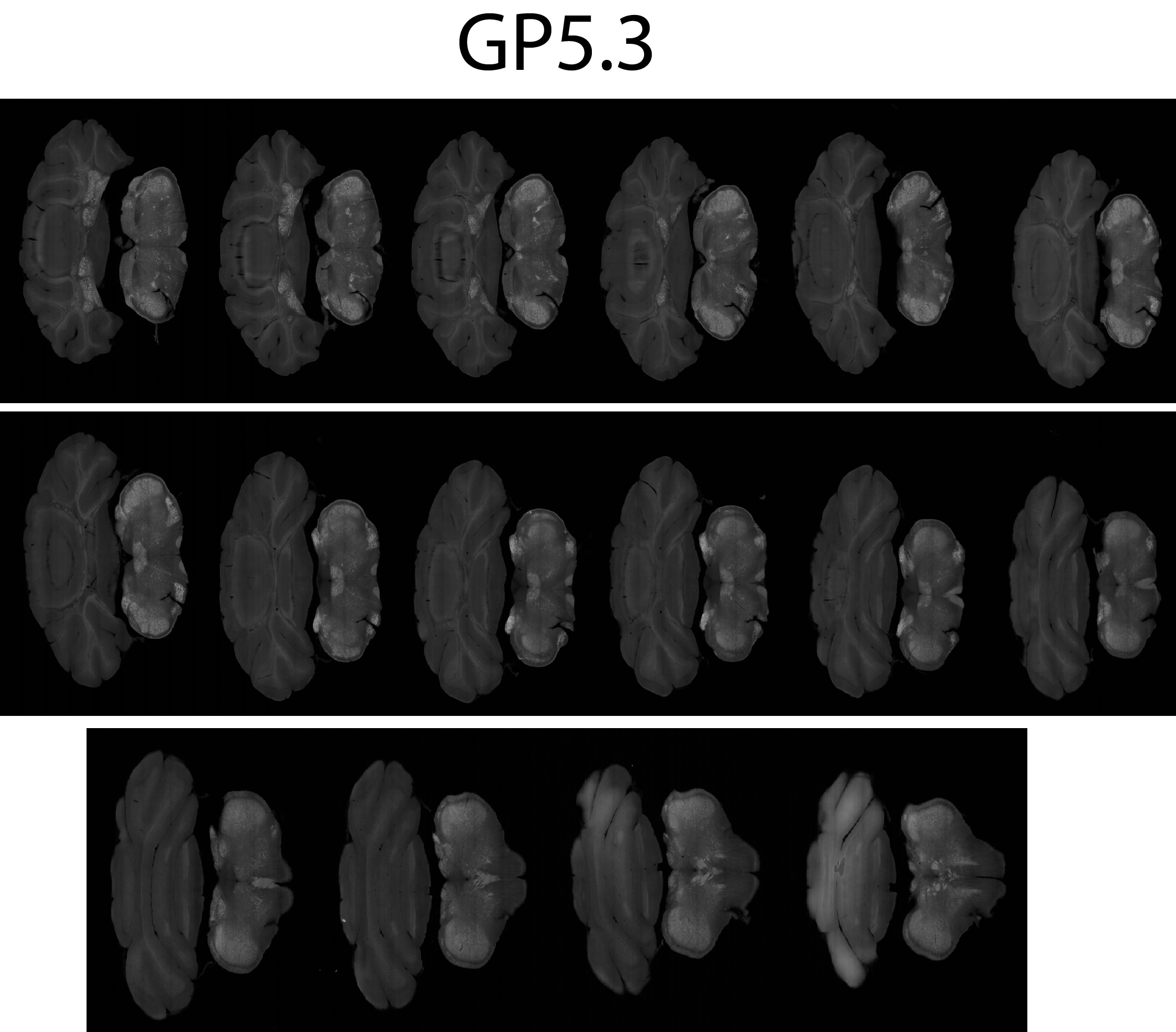


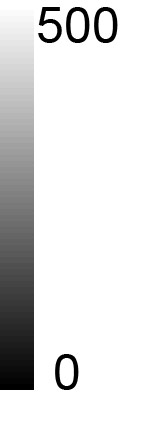

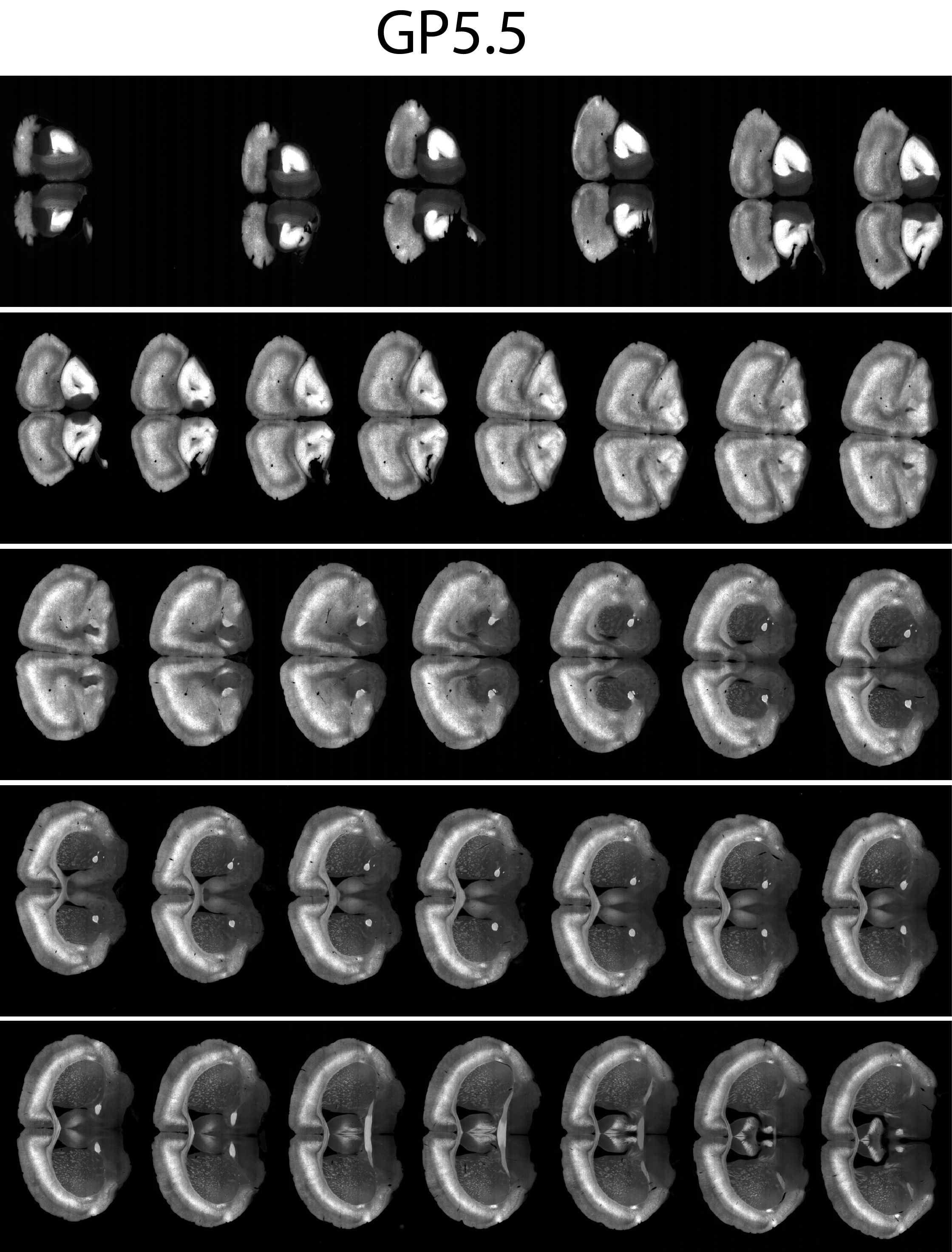


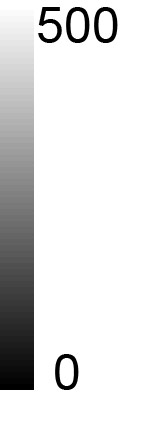

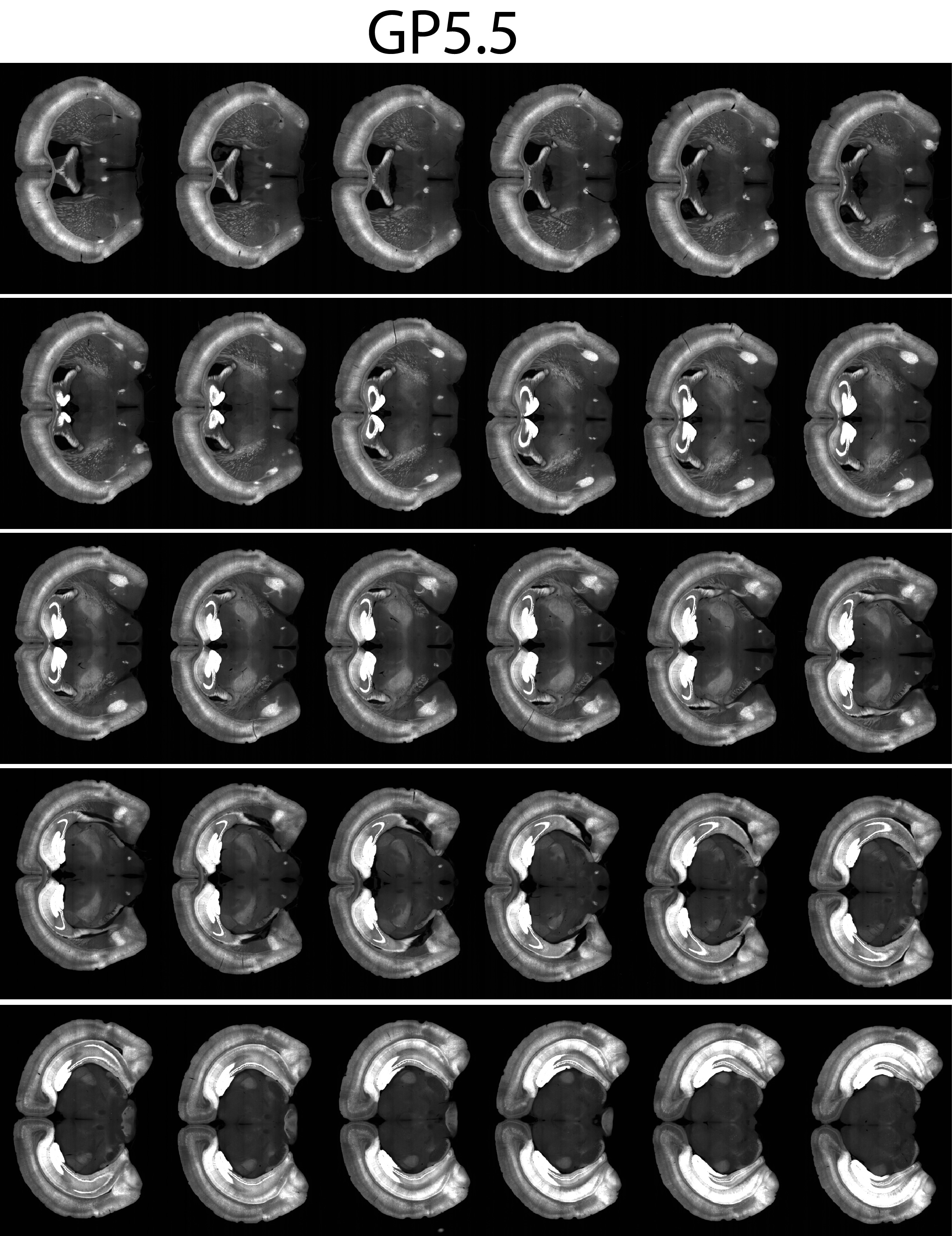


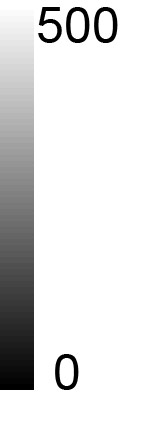

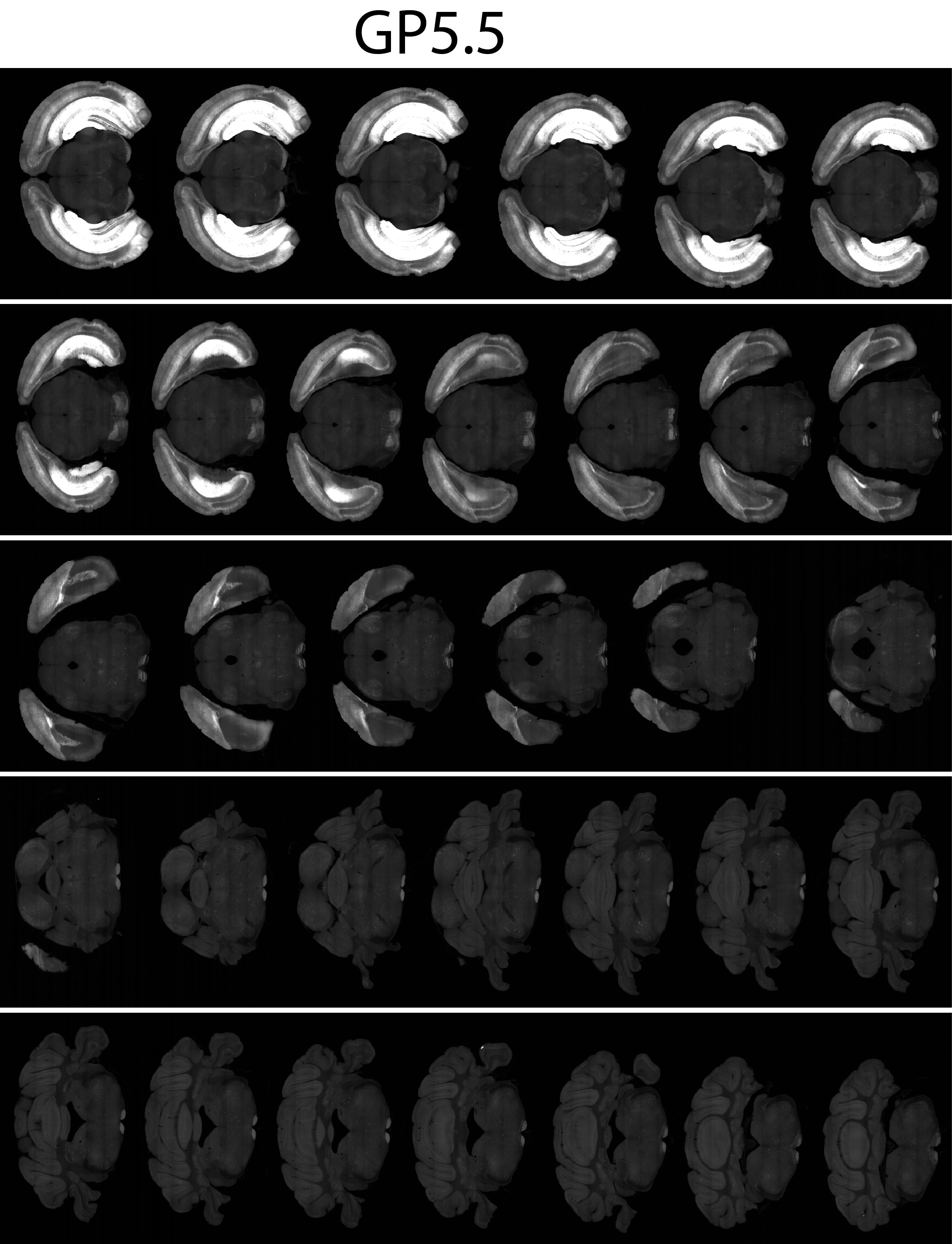


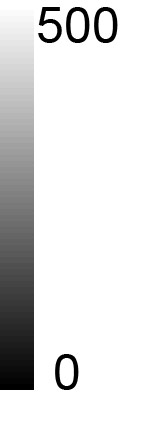

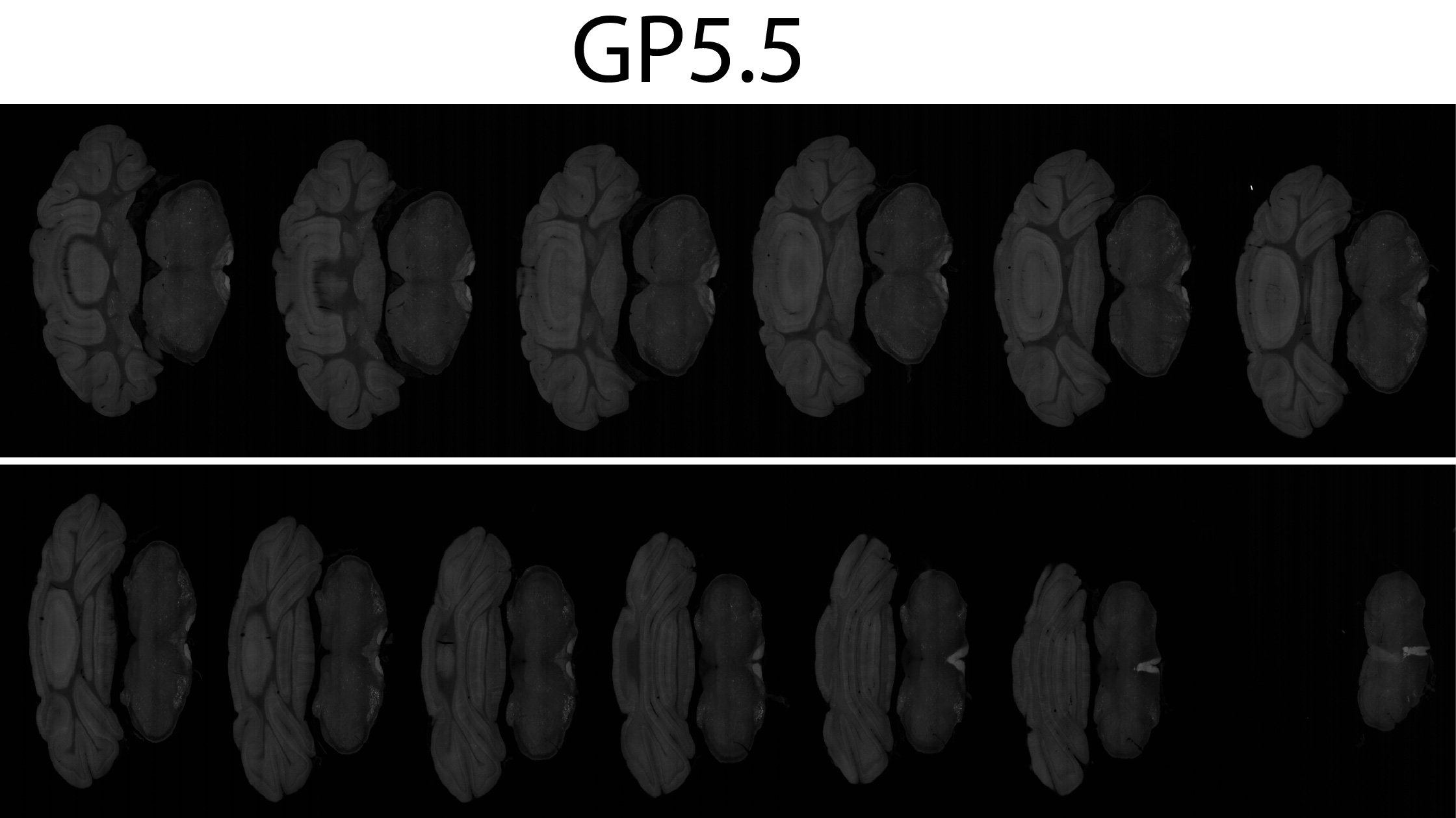


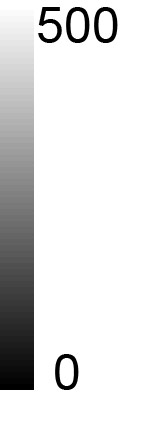

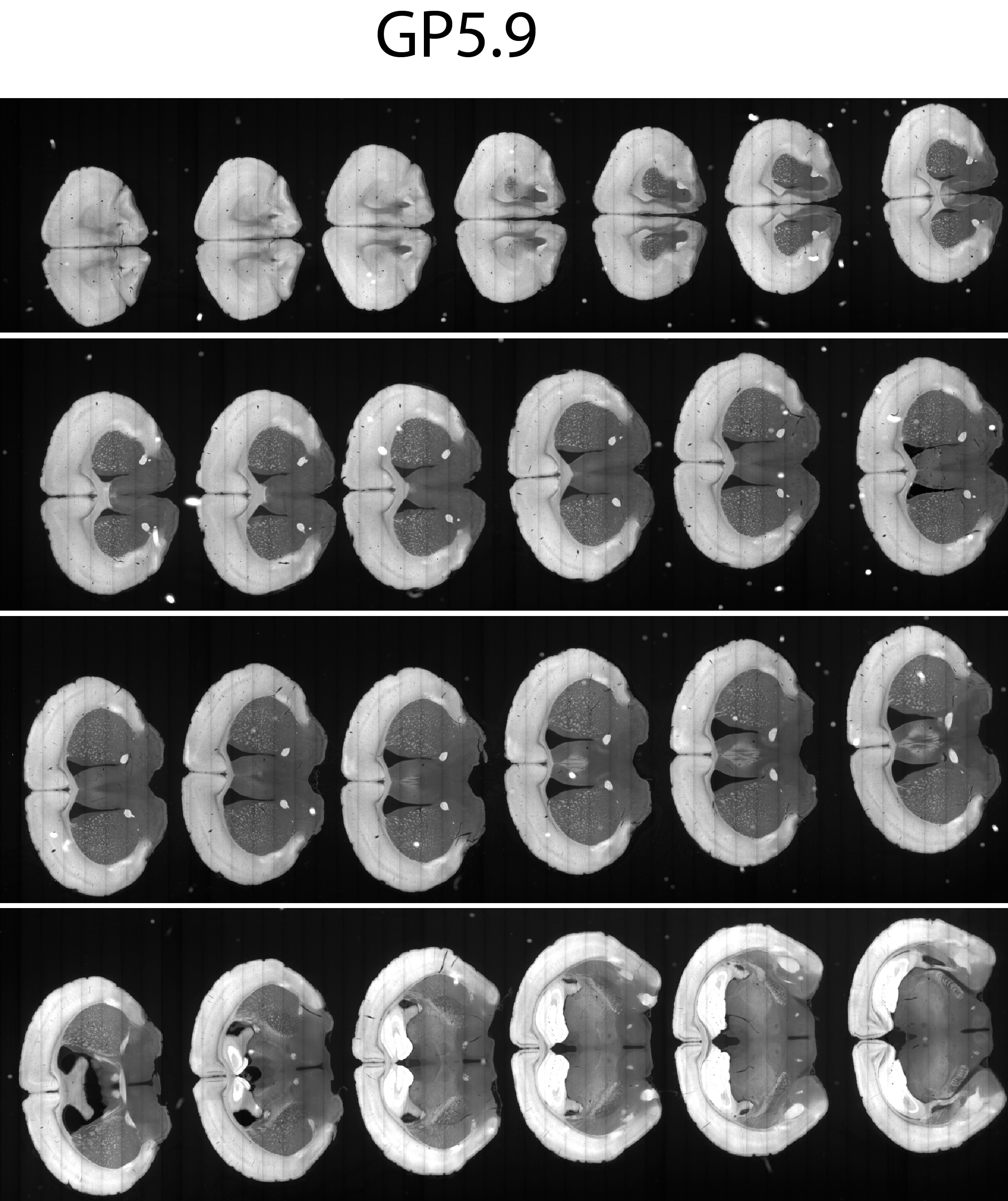

Supplement: Data S1 — Widefield images of GP lines coronal sections. Widefield microscopy images were taken using a slide scanner (Nanozoomer, Hamamatsu) with a ×20 0.75 NA air objective (Olympus). Imaging conditions were kept constant across time, but note the different greyscale range used for presenting the different lines. Sections thickness was 50 µm; every second sections was mounted and used for imaging (see Methods section for details). Sections were mounted from anterior to posterior. For several lines (such as GP4.2 and GP5.18) only a subset of sections were mounted. (DOCX) [file pone.0108697.s004.docx]
